# A Layered Organic Cathode for High-Energy, Fast-Charging, and Long-Lasting Li-Ion Batteries

**DOI:** 10.1021/acscentsci.3c01478

**Published:** 2024-01-18

**Authors:** Tianyang Chen, Harish Banda, Jiande Wang, Julius J. Oppenheim, Alessandro Franceschi, Mircea Dincǎ

**Affiliations:** † Department of Chemistry, Massachusetts Institute of Technology, Cambridge, Massachusetts 02139, United States; ‡ Department of Industrial Engineering, University of Bologna, Bologna 40136, Italy

## Abstract

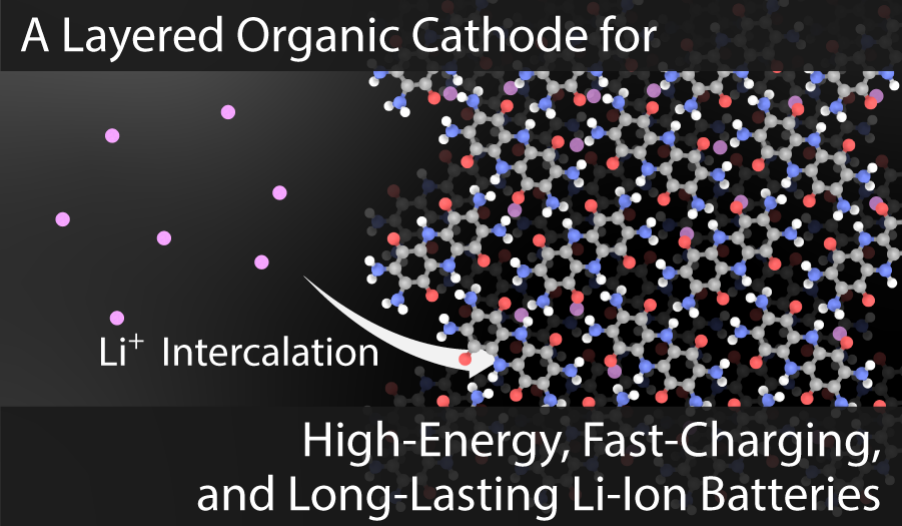

Eliminating the use of critical metals in cathode materials can accelerate global adoption of rechargeable lithium-ion batteries. Organic cathode materials, derived entirely from earth-abundant elements, are in principle ideal alternatives but have not yet challenged inorganic cathodes due to poor conductivity, low practical storage capacity, or poor cyclability. Here, we describe a layered organic electrode material whose high electrical conductivity, high storage capacity, and complete insolubility enable reversible intercalation of Li^+^ ions, allowing it to compete at the electrode level, in all relevant metrics, with inorganic-based lithium-ion battery cathodes. Our optimized cathode stores 306 mAh g^–1^_cathode_, delivers an energy density of 765 Wh kg^–1^_cathode_, higher than most cobalt-based cathodes, and can charge–discharge in as little as 6 min. These results demonstrate the operational competitiveness of sustainable organic electrode materials in practical batteries.

## Abstract 2

A metal-free layered organic cathode material for lithium-ion batteries intercalates Li^+^ and stores more energy with a shorter charging time than inorganic incumbents.

## Introduction

Lithium-ion batteries (LIBs) are dominant energy storage solutions for electrifying the transportation sector and are becoming increasingly important for decarbonizing the grid. Traditional cathodes for LIBs are made from inorganic oxides, especially those of Co, Ni, and Mn (e.g., LiCoO_2_ (LCO) and LiNi_1–*x*–*y*_Mn_*x*_Co_*y*_O_2_ (NMC)).^1^ Of these, cobalt poses severe limitations due to its scarcity and high social cost (e.g., child labor).^2,3^ Complete removal of cobalt has proven difficult, as oxide cathodes that are Co-free suffer from poor cyclability or capacity.^4,5^ Indeed, today, electric vehicles overwhelmingly use Co-based batteries. However, expansion of the global EV fleet is essentially impossible without the development of cobalt-free technologies.^6^ This has led to significant efforts in developing LIBs using more abundant and cost-effective lithium iron phosphate (LFP) as a cathode,^7,8^ despite LFP’s known inferior energy density relative to oxide-based cathodes and phosphate’s critical role in agriculture notwithstanding.^9−11^ Clearly, LIBs would benefit from the development of sustainable cathode technologies based on inexpensive, abundant precursors that can be sourced and scaled globally through more environmentally benign processes.

**Figure 1:**
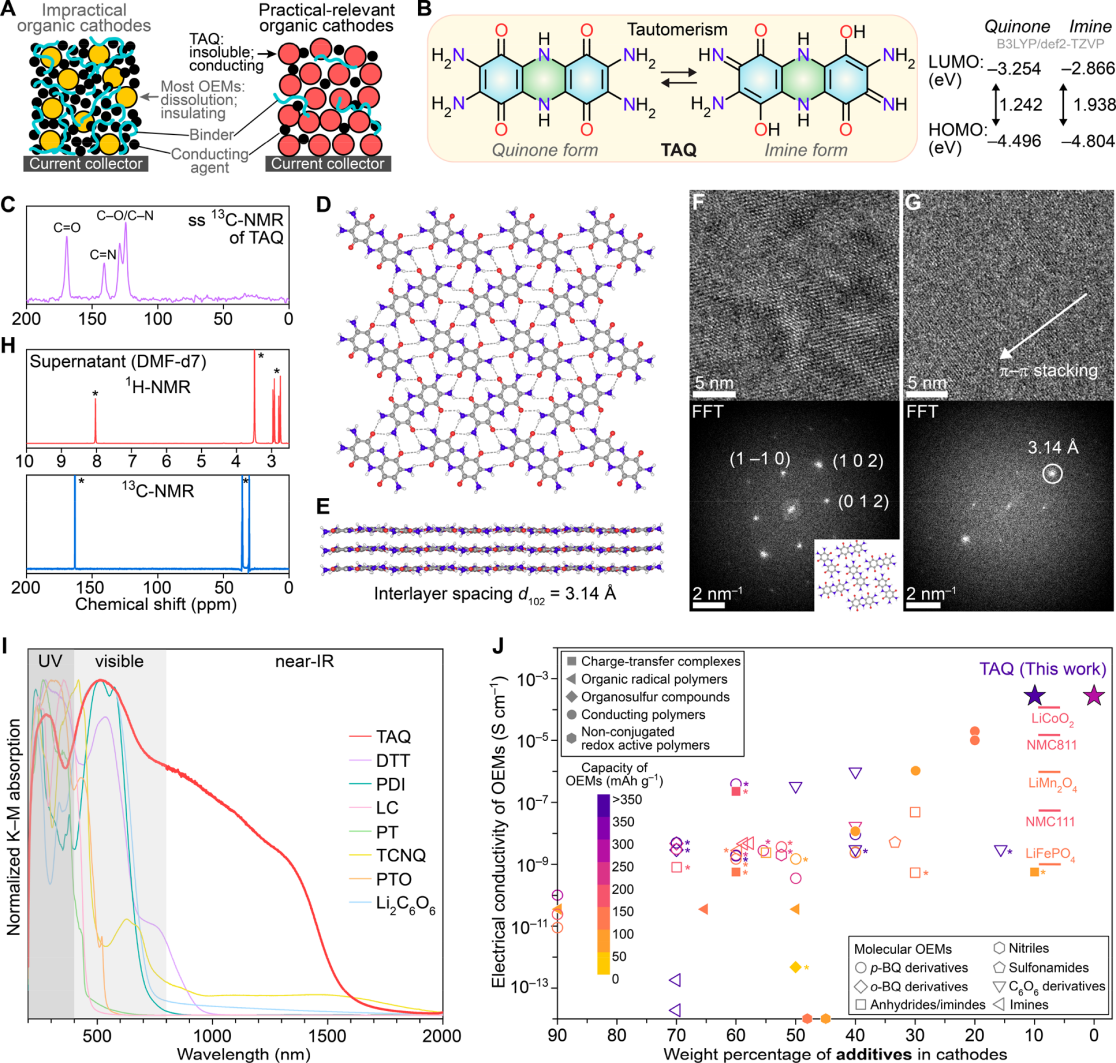
Characterization of TAQ. (A) Common organic cathodes with low active material content and TAQ-based cathodes with high and practical-relevant active material content. (B) The keto–enol tautomerism is represented by both quinone and imine forms with different energy levels. (C) Solid state ^13^C NMR spectrum confirms both quinone and imine forms. (D) A 2D layer of TAQ molecules formed by intermolecular hydrogen bonding (dashed lines). (E) π–π stacking of 2D layers with an interlayer spacing of 3.14 Å. (F, G) In-plane and out-of-plane molecular packing of TAQ observed in cryo-EM images. (H) ^1^H and ^13^C spectra of the supernatant obtained after heating TAQ in deuterated *N*,*N*-dimethylformamide (DMF-*d*_7_) at 120 °C for 12 h. Asterisks indicate solvent peaks. (I) DRUV–vis spectra of TAQ and other prototypical OEMs. (J) Electrical conductivities of different classes of OEMs, TAQ, and state-of-the-art inorganic electrode materials versus typical amounts of additives used for electrode fabrication. Poly(acetylene), poly(pyrrole), poly(thiophene), and poly(aniline) are excluded because they operate through anion insertion instead of Li^+^ insertion. Asterisks indicate rapid capacity decay during the first few cycles, mainly due to dissolution. The details of OEMs in I and J are summarized in Figures S7 and S9.

Redox-active organic materials, derived entirely from earth-abundant elements, offer just such an opportunity.^12,13^ They benefit from excellent compositional diversity and structural tunability while offering requisite synthetic control for targeted designs as cathode materials for not only LIBs but also other battery systems such as Na-ion or Zn-ion batteries. Although the merits of replacing inorganic cathodes with organic electrode materials (OEMs) have long been appreciated in the literature,^14−16^ material candidates in this class that deliver comprehensive performance along all metrics relevant for practical LIBs have remained elusive. From a design perspective, small organic molecules offer high specific capacities by virtue of a dense arrangement of redox sites and their low molar masses relative to those of redox-active polymers or framework materials. However, discrete molecules typically have low bulk conductivity and often dissolve in battery electrolytes, which lead to poor utilization of redox sites, low charge–discharge rates, and poor cycling stability.^17^ These issues are routinely managed by adding electrically conducting and/or stabilizing polymeric additives typically exceeding 50 wt %, which greatly reduce the effective capacity, rendering the electrodes impractical.^18−25^ Alternative strategies to polymerize redox-active OEM candidates or to immobilize them into host frameworks often require compromise in at least one of the critical practical metrics.^15,16^ As such, there continues to be a strong interest in designing intrinsically insoluble and electrically conducting OEMs that exhibit high specific capacity at appropriate cathodic voltages (>2.0 V) for LIBs (Figure 1A). To our knowledge, OEMs for LIBs that fulfill all these criteria so as to rival inorganic cathodes are not known.^14−17^

Here, we demonstrate that bis-tetraaminobenzoquinone (TAQ), a fused conjugated molecule with a layered solid-state structure, functions as a fast-charging, high-energy, and long-lasting OEM for LIB cathodes. As reported recently,^26^ TAQ is characterized by a dense arrangement of redox-active carbonyl (C=O) and imine (C=N) groups on a conjugated backbone. Two 2e^–^ redox couples give TAQ a high theoretical specific capacity of 356 mAh g^–1^. Strong intermolecular hydrogen bonding and donor–acceptor (D-A) π–π interactions in TAQ render it insoluble in common battery electrolytes and impart extended electronic delocalization that leads to high bulk electrical conductivity. These features allow optimized electrodes that comprise at least 90 wt % of TAQ to reversibly store charge and cycle safely for over 2000 cycles, strongly contrasting with the issues of electrode dissolution chronically experienced in known OEMs (Figure 1A). The two-dimensional (2D) layered arrangement of TAQ molecules enables facile insertion/extraction of Li^+^ between the layers and delivers excellent rate capabilities even at full charging in as little as 3 min. Optimized electrodes deliver excellent performance even at high areal mass loadings up to 16 mg cm^–2^ with an areal capacity up to 3.52 mAh cm^–2^, which is on par with commercial lithium-ion batteries,^27^ comprehensively demonstrating the viability of TAQ in practical LIBs.

## Results and Discussion

### Crystal Structure, Electronic Structure, and Electrical Conductivity of TAQ

TAQ is obtained in gram quantities by Michael condensation of tetraamino-*p*-benzoquinone (see Supporting Information, Materials and Methods) as highly crystalline microrods, whose identity was verified by wide-angle X-ray scattering (WAXS), scanning electron microscopy (SEM), and cryogenic electron microscopy (Cryo-EM) (Figure S1A–C). TAQ exhibits significant keto–enol tautomerization (Figure 1B) through the conjugation of carbonyl and amino groups within the two diaminobenzoquinone moieties that are connected by a dihydropyrazine core (Table S1), leading to both quinone and imine forms. The contribution of the imine tautomer is evidenced by the C=N signal at 140.8 ppm in its ^13^C solid-state NMR (ssNMR) spectrum (Figures 1C, S2) and from the partial double bond character of the two C–NH_2_ bonds evidenced in the single crystal structure (Figure S1D). The dihydropyrazine linkage is crucial for enabling significant electronic delocalization between the two neighboring diaminobenzoquinone moieties. This distinguishes it from the related molecule tetraamino-phenazine-1,4,6,9-tetrone (Figure S3),^28^ whose calculated HOMO–LUMO gap, 2.212 eV, is nearly 1 eV higher than that of TAQ, 1.242 eV (Figure 1B, Table S2).

Planar TAQ molecules are surrounded by six neighbors and closely pack into two-dimensional layers through pervasive intermolecular hydrogen bonding between carbonyl and amine/imine functional groups (Figure 1D). These layers stack through strong donor–acceptor π–π interactions with a short interlayer distance of 3.14 Å (Figure 1E). High-resolution cryo-EM images of TAQ (Figures 1F,G, S4–S6) and the corresponding fast Fourier transform (FFT) further confirmed its dense molecular packing and the out-of-plane close stacking of 2D layers. Owing to its compact solid-state packing, TAQ exhibits very low solubility in common organic solvents and battery electrolytes (Figure S1E). Notably, heating TAQ in deuterated *N*,*N*-dimethylformamide at 120 °C overnight leads to little dissolution, as verified by the absence of TAQ signals in the ^1^H and ^13^C spectra of the supernatant (Figure 1H). The unusually low solubility of TAQ, even at an elevated temperature, stands in stark contrast to other OEMs reported for LIBs (Figure S7) and is key for a long cycling lifetime.

Owing to a combination of intramolecular extended conjugation, intermolecular hydrogen bonding, and interlayer π–π stacking, TAQ also exhibits broadband electronic absorption from 200 to nearly 1600 nm (Figure 1G), indicating significant electronic delocalization. This again contrasts with any other molecular OEMs, and some prototypical charge-transfer complexes,^29^ conjugated polymers,^30^ and organic radical polymers,^31^ which show absorption only below 800 nm (Figures 1I, S8) and thus have poor electronic delocalization. TAQ also exhibits an optical gap of ∼0.8 eV, which is comparable with doped poly(pyrrole).^32^ The electron paramagnetic resonance (EPR) spectrum of TAQ (Figure S9A) revealed the presence of delocalized organic spins, as verified by the Dysonian line shape of the signal and the corresponding line shape asymmetry indicator (i.e., the ratio of the positive to the negative part of the EPR signal), 1.32, which is similar to some single-walled carbon nanotubes.^33^ The organic spins, which likely originate from the partial oxidation of the dihydropyrazine moiety, have a Curie spin density of 0.032 per TAQ molecule, as indicated by the variable temperature direct current susceptibility measurement of TAQ (Figure S9B), and delocalize through extended conjugation. Because of these features, TAQ exhibits semiconducting behavior with a charge transport activation energy of 319 meV and room-temperature electrical conductivity of up to 2.1 × 10^–4^ S cm^–1^ (Figure S9C,D). This is substantially higher than most molecular OEMs, which are either poor conductors or insulators (Figure S10, Table S3), and is also higher than or comparable with charge-transfer complexes, organosulfur compounds, conjugated polymers, and organic radical polymers.^29−31^ Remarkably, the electrical conductivity of TAQ is on par with that of LCO and state-of-the-art NMC,^34,35^ and is approximately 5 orders of magnitude higher than that of LFP.^36^ These favorable properties of TAQ allow fabrication of battery electrodes with little to no additives. In contrast, most, if not all, OEMs routinely need at least 40 wt % additives (Figures 1J, S9E).^16^

### Neat TAQ Cathodes

**Figure 2:**
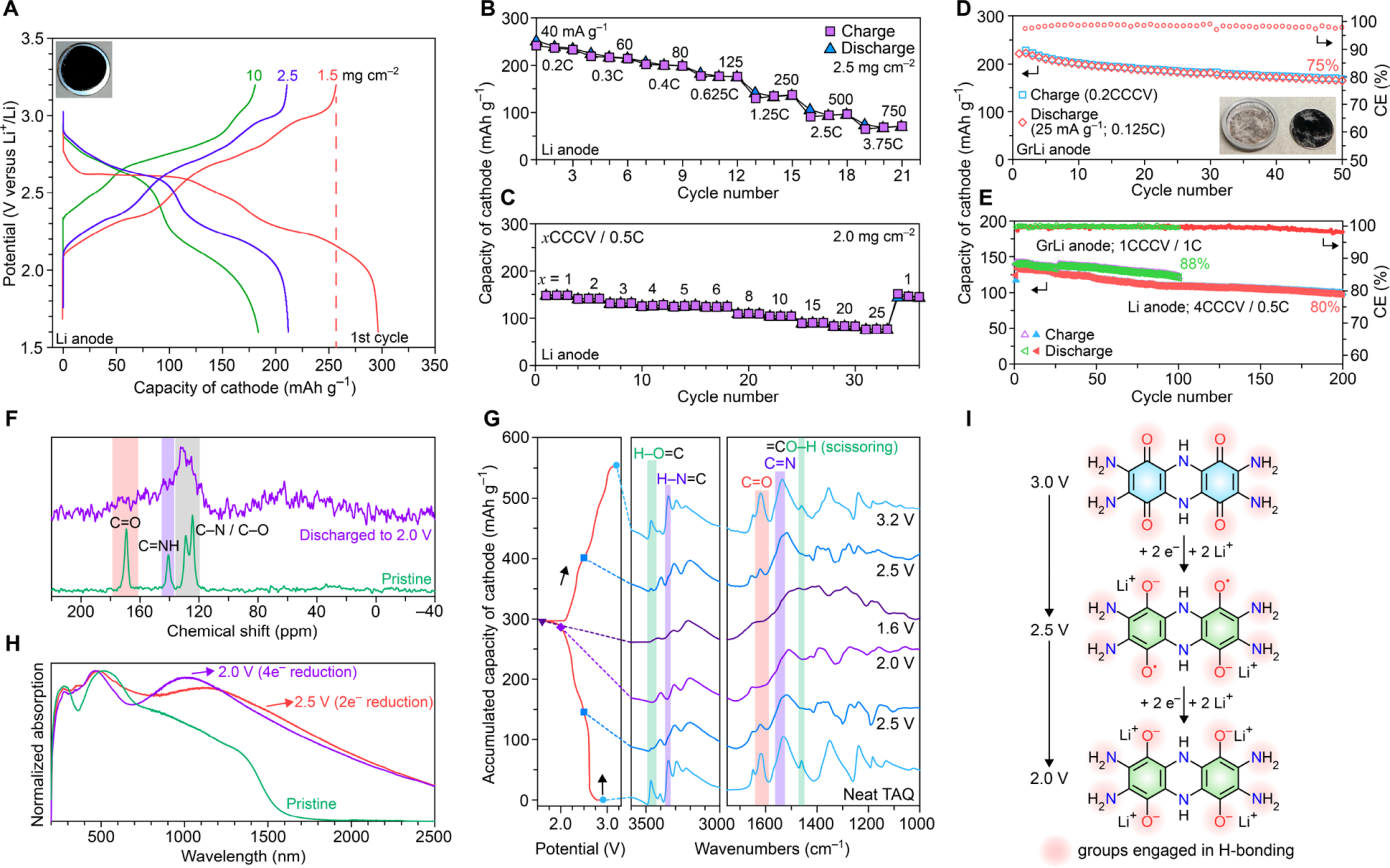
Characterization of neat TAQ electrodes. (A) GCD voltage profiles of three neat TAQ||Li cells at 25 mA g^–1^. Increasing the electrode mass loadings from 1.5 to 10 mg cm^–2^ leads to 70% retention of reversible capacity. (B) Power capability of neat TAQ electrodes recorded from 40 mA g^–1^ (0.2 C) to 750 mA g^–1^ (3.75 C). (C) Power capability recorded at various CCCV charging rates and a discharge rate of 0.5 C. (D) Slow cycling of a TAQ/GrLi full-cell. Inset shows the photo of a disassembled coin cell after cycling. (E) Cycling studies of a neat TAQ||Li half cell and a neat TAQ||GrLi full cell at higher rates: 1CCCV/1C and 4CCCV/0.5C. (F) Ex-situ ^13^C ssNMR spectrum of neat TAQ electrode discharged to 2.0 V shows the disappearance of both C=N and C=O signals. (G) Ex-situ FTIR spectra of neat TAQ electrodes at various stages of a discharge–charge cycle show reversible changes in chemical signatures. (H) Ex-situ DRUV–vis spectra of TAQ at various states of discharge. (I) Schematic representation of the redox mechanism of TAQ.

Due to its high conductivity and poor solubility, neat TAQ (without conductive and binder additives) can be directly used as a cathode (see Supporting Information, Methods) in Li-ion half cells using lithium anodes and commercial LP30 electrolyte (1.0 M LiPF_6_ in 1:1 ethylene carbonate (EC)/dimethyl carbonate (DMC)). Galvanostatic charge–discharge (GCD) voltage profiles (Figure 2A) recorded at 25 mA g^–1^ (0.125 C) between 1.6 and 3.2 V (all potentials are referenced to the Li^+^/Li couple unless otherwise noted) exhibit initial discharge and charge capacities of 297 mAh g^–1^ and 258 mAh g^–1^ based on the cathode mass, respectively. The initial Coulombic efficiency (ICE) is 87%, in line with the 80%–86% ICEs observed in commercial NMC cathodes.^37^ d*Q*/d*V* curves (Figure S11A) exhibit four plateaus centered around 2.3, 2.7, 2.8, and 3.0 V during charging, where the middle two plateaus are hardly distinguishable in the voltage profile (Figure 2A). In comparison, two distinct plateaus between 2.9 V–2.5 and 2.3 V–2.0 V were observed during discharge, which are centered at 2.2 and 2.6 V, respectively. Both of these discharge plateaus store nearly equal amounts of charge, ∼130 mAh g^–1^, resulting in a nominal discharge potential of 2.5 V. Replacing lithium anodes with prelithiated graphite anodes (GrLi, see Supporting Information, Methods and Figure S11B) gives similar voltage profiles and capacity. Increasing the areal mass loading of neat TAQ to 10 mg cm^–2^, a loading rarely reached with organic cathodes even when these are mixed with 50 wt % of carbon, maintained a capacity as high as 181 mAh g^–1^ (i.e., 1.8 mAh cm^–2^), compared to 210 mAh g^–1^ at a loading of 2.5 mg cm^–2^ (Figure 2A). Faster charging rates of 0.4 C and 2.5 C delivered discharge capacities of 207 and 106 mAh g^–1^ (Figure 2B), respectively. Furthermore, 10 and 6 min constant-current constant-voltage (CCCV) charging delivered discharge capacities of 125 and 105 mAh g^–1^, respectively (Figure 2C). Diffusion coefficients of Li^+^ in neat TAQ electrodes obtained using galvanostatic intermittent titration techniques (GITT) revealed values of ∼10^–10^ cm^2^ s^–1^ throughout the discharging/charging processes (Figure S11C,D). These coefficients are similar to those of state-of-the-art optimized inorganic cathodes^35^ and highlight facile Li^+^ diffusion within TAQ crystals and in bulk neat TAQ.

Cycling studies at low charge–discharge rates are generally employed to evaluate the ability of OEMs to withstand dissolution into the electrolyte under operating conditions. Neat TAQ electrodes are stable to at least 50 cycles at 0.2 CCCV/0.125 C, at 100% depth of discharge (DOD), with a capacity retention of 75% and an average CE greater than 98% (Figures 2D, S11E). No electrode dissolution was observed after cycling, but TAQ rods did show fracturing into flakes, as verified by ex-situ SEM images (Figure S12). Cycling at higher rates of 1 CCCV/1 C and 4 CCCV/0.5 C maintains near 100% CE with a capacity retention of 88% over 100 cycles and 80% over 200 cycles (Figure 2E). This cycling performance of *neat* OEM electrodes is unprecedented and serves as a testament to the facile ion diffusion and charge transport ability of TAQ, as well as its virtual insolubility in common battery electrolytes.

Employing neat TAQ as the cathode also enabled direct spectroscopic analysis of the redox processes without interference from the electrode additives. An ex-situ ^13^C ssNMR spectrum of TAQ discharged to 2.0 V (Figure 2F) revealed the disappearance of both C=N (140.8 ppm) and C=O (169.2 ppm) signals, indicating that both functional groups are reduced during discharge despite the overall spectrum broadening. An EPR spectrum of discharged TAQ (Figure S13A) revealed significantly increased radical content, corresponding to approximately 0.85 free radical per TAQ, close to the theoretical value of 1 radical per TAQ for a sample with a discharge capacity of 268.3 mAh g^–1^ (∼75% of the theoretical capacity). A fit of the EPR spectrum attributed the signal to a TAQ biradical (Figure S13B). Ex-situ FTIR spectra of TAQ measured at different potentials (Figure 2G) exhibit a gradual decrease and recovery of the C=O and C=N stretching bands at 1618 and 1531 cm^–1^, respectively, reflecting the discharge and recharge processes. Interestingly, the O–H (3464 cm^–1^) stretching band, imine N–H (3346 cm^–1^) stretching band, and the O–H scissoring band coupled to dihydropyrazine ring modes (1460 cm^–1^), all of which stem from the imine tautomer (Figure S14), almost completely disappear when TAQ is discharged to the first plateau at ∼2.5 V. Given that the quinone form has a lower LUMO energy (Figure 1B), which in principle translates to a higher reduction potential relative to the imine form, is more likely to accept electrons initially during discharge, which simultaneously shifts the tautomerization equilibrium from imine to quinone. This is supported by ex-situ XPS data of TAQ discharged to 2.5 V (Figure S15), which confirm the disappearance of imine N and C–OH from the imine tautomer of TAQ, and the reduction of quinoid (C=O) to benzenoid (C–O^–^). TAQ discharged to 2.5 V is proposed to contain diradicals, which is supported by its DRUV–vis spectrum, revealing a significant polaronic band centered around 1200 nm (Figure 2H). Subsequent two-electron reduction gives the fully reduced TAQ, corresponding to the second plateau centered around 2.2 V. Ex-situ DRUV–vis spectra of TAQ discharged to both 2.5 and 2.0 V (Figure S13C) also reveal less intramolecular electronic delocalization relative to charged TAQ due to the lack of tautomerization (Figure 2I), as verified by the blue-shifted absorption at 2.67 eV (2.0 V) and 2.56 eV (2.5 V) relative to 2.41 eV for charged TAQ. Surprisingly, discharge promotes intermolecular electronic delocalization, indicated by the significantly enhanced polaronic absorption in the near-IR (Figure 2H). The result is that the electrical conductivity of TAQ discharged to 2.0 V remains essentially unchanged compared to that of its charged state (Figure S13D). The redox behavior of TAQ upon prolonged cycling does not affect its molecular structure: FTIR spectra and XPS analysis of electrodes cycled for over 100 cycles at a low rate are nearly indistinguishable from those of pristine electrodes (Figures S16, S17). Overall, these features establish the redox cycling of TAQ as a two-step, four-electron process, corresponding to a high theoretical capacity of 356 mAh g^–1^.

### Optimized TAQ cathodes

Mixing TAQ with as little as 10 wt % additives further enhances its performance to reach near theoretical capacity. Specifically, carboxymethyl cellulose (CMC) and/or styrene butadiene rubber (SBR) allow the formulation of TAQ slurries in water (see Supporting Information, Methods), more environmentally friendly than *N*-methylpyrrolidone,^38^ and improve performance without sacrificing electrode-level metrics.

**Figure 3:**
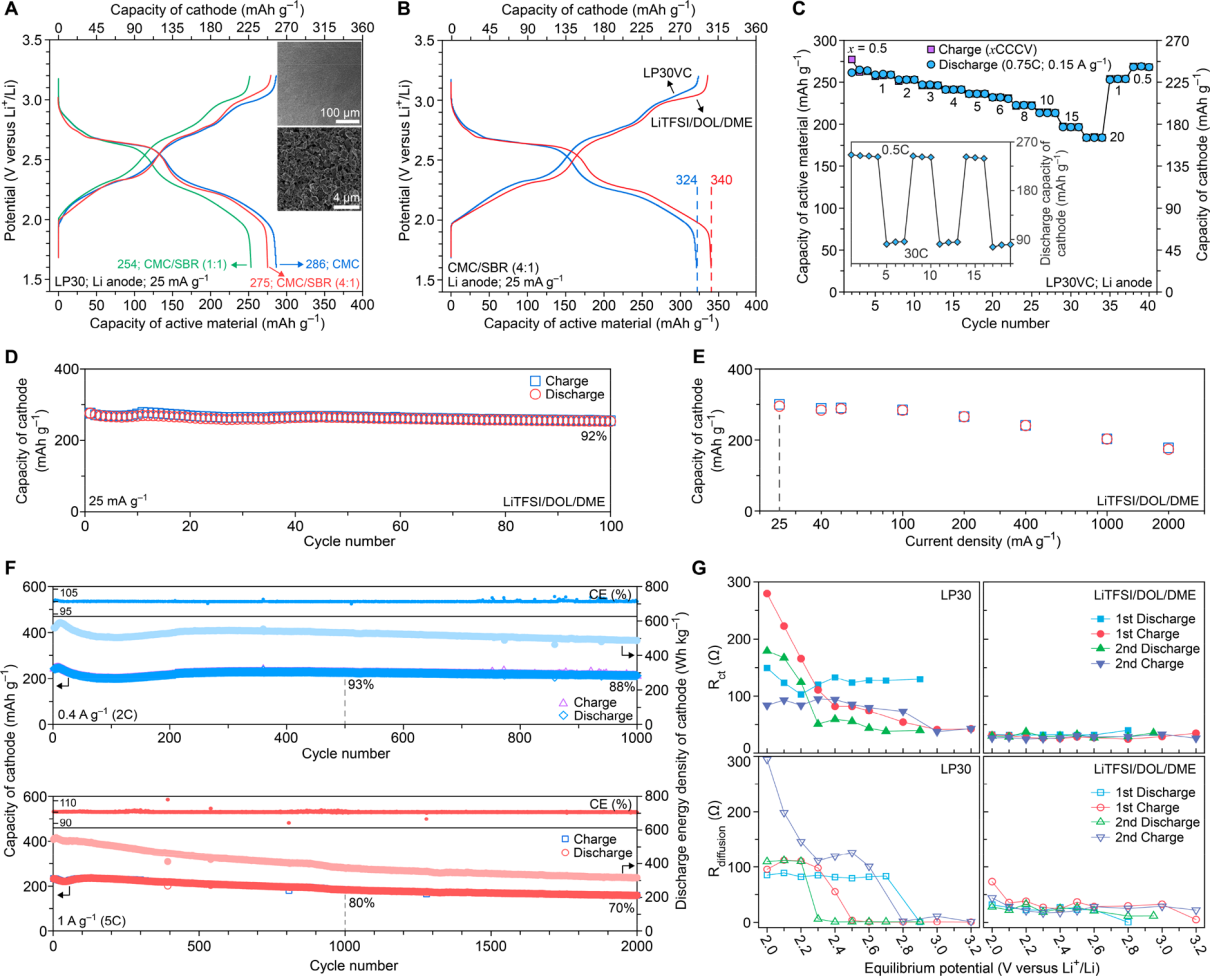
Performance of composite TAQ electrodes with 90 wt % active material. (A) GCD voltage profiles for various additive compositions in LP30 electrolyte. Insets are SEM images of composite electrodes using a CMC:SBR = 4:1 (optimized electrode). (B) GCD voltage profiles of optimized electrodes in different electrolytes. (C) Power capability studies at constant current discharge of 0.75 C and charge from 0.5 to 20 CCCV. Inset shows continuous switching between cycling at 0.5 C and 30 C. (D) Cycling of optimized electrode at a constant charge and discharge current density of 25 mA g^–1^ at 100% DOD in LiTFSI/DOL/DME. (E) Average capacity of cathodes over 600 h-cycling at current densities ranging from 25 mA g^–1^ to 2000 mA g^–1^. (F) Cycling at 0.4 A g^–1^ (2 C/2 C for charge/discharge) and 1 A g^–1^ (5 C/5 C for charge/discharge) at 100% DOD in LiTFSI/DOL/DME. (G) *R*_ct_ and *R*_diffusion_ of TAQ/CMC/SBR||Li cells in LP30 and LiTFSI/DOL/DME.

Compared to neat TAQ electrodes, TAQ/CMC composite electrodes delivered greater reversible capacity of 286 mAh g^–1^_TAQ_ (Li anode) or 299 mAh g^–1^_TAQ_ (GrLi anode) at 25 mA g^–1^ in LP30, enhanced ICE of 92–94% (Figures 3A, S18A,B), significantly improved rate capability, and greater cycling stability (Figure S18C–F). A proof-of-concept TAQ/CMC||GrLi full cell with a nearly balanced negative/positive electrode capacity ratio (N/P) of 1.1 exhibited a cathode capacity of 180 mAh g^–1^ (Figure S19).

Despite their superior performance relative to neat TAQ, TAQ/CMC electrodes suffered from poor adhesion to the current collectors, which prompted us to use increments of the SBR additive for optimized electrode formulations. Although increasing SBR content decreases the overall electrode capacity (Figure 3A), the CMC/SBR combination, common in commercial graphite anodes, substantially enhances the mechanical integrity and adhesion of TAQ to the stainless steel current collector (Figure S20). A CMC:SBR ratio of 4:1 provided a good balance of the capacity and mechanical properties, leading to optimized electrodes with uniform and robust coatings (Figure 3A insets) and a reversible capacity of 275 mAh g^–1^_TAQ_. The CMC/SBR composite further increases the stability of already insoluble TAQ, such that TAQ/CMC/SBR electrodes show very limited dissolution in LP30 even at 100 °C after 24 h (Figure S21).

Further increase in capacity is possible by addition of 5% vinylene carbonate (VC) to the LP30 electrolyte (LP30VC), a common strategy in commercial devices.^39^ This led to an initial discharge capacity of 356 mAh g^–1^_TAQ_, equal to TAQ’s theoretical capacity, and an improved reversible capacity of 324 mAh g^–1^_TAQ_ (Figure 3B). Rate capability studies in LP30VC revealed a cathode capacity of 192 mAh g^–1^ at 10 CCCV and 166 mAh g^–1^ at 20 CCCV (Figure 3C), which correspond to total charging times of 6 and 3 min, with a capacity retention of 80% and 70%, respectively, relative to 240 mAh g^–1^ at 0.5 CCCV (i.e., a total charging time of 2 h). Stable fast-switching between cathode capacities of 239 mAh g^–1^ at 0.5 C and 90 mAh g^–1^ at 30 C (charging time of 2 min) further highlights the high power capability of TAQ (Figure 3C inset). Replacing LP30VC with LP40VC (1.0 M LiPF_6_ in 1:1 EC/diethyl carbonate (DEC) with 5% VC) enhanced the performance in cells with GrLi anodes (Figure S22).

Compared with carbonate electrolytes, ether-based electrolytes such as 1.0 M lithium bis(trifluoromethanesulfonyl)imide in 1:1 1,3-dioxolane/dimethoxyethane (LiTFSI/DOL/DME) are known to deliver better CE with lithium anodes.^40,41^ TAQ composite electrodes delivered reversible capacities of up to 340 mAh g^–1^_TAQ_ (Figure 3B) with enhanced ICE of 95–100% (Figure S23) based on five cells, and stable cycling at 25 mA g^–1^ and 100% DOD in LiTFSI/DOL/DME, exhibiting a cathode capacity of 254 mAh g^–1^ after 100 cycles (i.e., 92% retention after more than three months of continuous cycling; Figure 3D). Cycling studies at higher current densities ranging from 40 mA g^–1^ (0.2 C) to 2000 mA g^–1^ (10 C) revealed a limited decrease of the average cathode capacity over 600-h cycling from 256 to 157 mAh g^–1^ (Figure 3E, Table S4), suggesting both outstanding power performance and cycling stability. Specifically, cycling studies at 0.4 A g^–1^ (2C) and 1 A g^–1^ (5C) revealed a cathode capacity of 213 mAh g^–1^ after 1000 cycles and 159 mAh g^–1^ after 2000 cycles (Figure 3F), corresponding to capacity retention of 88% and 70%, respectively. More importantly, TAQ’s molecular structure remains unchanged upon prolonged cycling (Figure S24). GITT studies revealed slightly higher diffusion coefficients of Li^+^ in LiTFSI/DOL/DME (∼10^–9^ cm^2^ s^–1^) relative to that of LP30 (∼10^–10^ cm^2^ s^–1^) (Figure S25). Significantly lower charge transfer resistances (*R*_ct_), ranging from 20 to 40 Ω during the whole charge/discharge process, were observed in LiTFSI/DOL/DME relative to LP30 (Figure 3G, S26, S27). Ion diffusion resistances (*R*_diffusion_) remained below 40 Ω from 2.1 to 3.2 V in LiTFSI/DOL/DME, superior to the step-like increase of *R*_diffusion_ at a higher degree of discharge in LP30. Nevertheless, the *R*_ct_ and *R*_diffusion_ values observed in both LP30 and LiTFSI/DOL/DME are among the lowest values observed for any cathode materials at similar active content levels, and lower even than organic cathodes mixed with significant amounts of conducting additives.^42^ Overall, these metrics are indicative of rapid and reversible Li ion intercalation in TAQ, which enable its function as a cathode against metallic Li and prelithiated graphite anodes.

### Structural and Morphological Evolution

**Figure 4:**
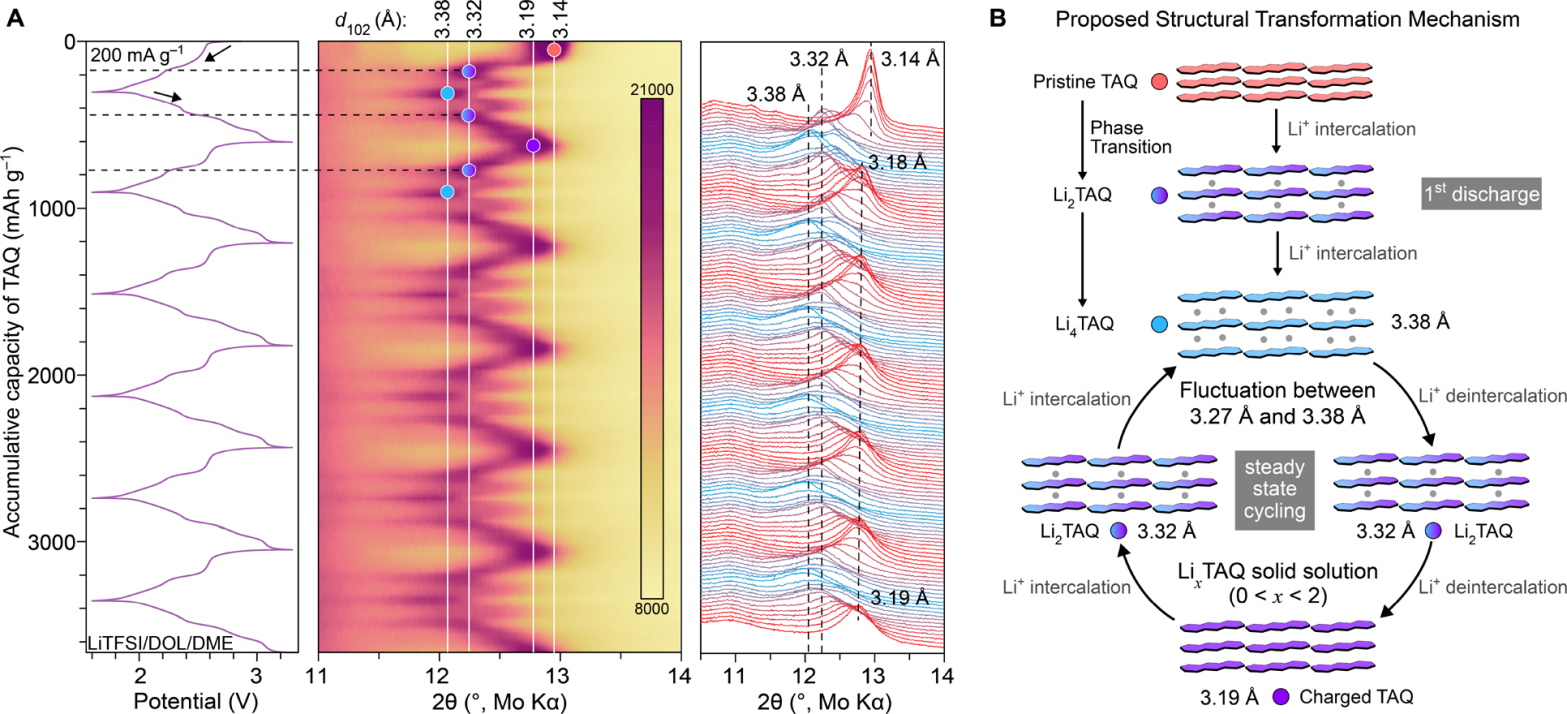
Structural evolution of TAQ during charge/discharge. (A) In-operando PXRD patterns in the region of the (102) reflection and the corresponding potential-capacity profile of a TAQ cell in LiTFSI/DOL/DME cycled six times at 200 mA g^–1^. (B) Diagram representing the proposed progression of TAQ during cycling. The values in Å represent the interlayer spacing for the TAQ under various charging/discharging states.

TAQ’s crystallinity and extreme insolubility enabled structural studies of Li^+^ intercalation and deintercalation through a combination of in-operando powder X-ray diffraction (PXRD; Figure S28) and ex-situ electron microscopy. Electrodes of TAQ were prepared as free-standing films, suitable for in-operando experiments, with 30 wt % carbon black and 10 wt % polytetrafluoroethylene binder (see Supporting Information, Methods). Cycling was performed at a practically relevant current density of 200 mA g^–1^. Tellingly, the interlayer distance, *d*_102_, which appears at 3.14 Å in pristine TAQ, increases to 3.32 Å upon discharging through the first cycle, where TAQ undergoes Li^+^ intercalation and a phase transition to Li_2_TAQ (Figure 4A). Upon further discharging and formation of Li_4_TAQ, the interlayer distance fluctuates between 3.27 and 3.38 Å (Figures 4B, S29), as expected for the accommodation of both four Li^+^ ions and a greater electrostatic repulsion between tetranionic TAQ^4–^ molecules. The interlayer distance fluctuates once again within the same range upon charging, when Li_4_TAQ first transforms to Li_2_TAQ, but the PXRD data do not provide sufficient resolution to describe structural details of this transformation at this stage (see related discussion in Supporting Information). Interestingly, the structural progression between the limiting compositions, Li_2_TAQ and recharged TAQ, is smooth, as verified by the continuous shifting of interlayer distance between 3.32 and 3.19 Å. This is indicative of a Li^+^ intercalation/deintercalation mechanism that occurs in a single-phase solid solution Li_*x*_TAQ (*x* = 0–2) during steady state cycling (Figure 4B).^43^ The coherent phase transformations through the formation of extended solid solutions during charge–discharge, likely enabled by the flexible layered structure and the strong in-plane molecular packing of TAQ, account for the good rate capability of TAQ cathodes. A similar smooth transition between charged and discharged phases is also key for enabling the high-rate performance of nanosized olivine phosphate cathodes.^44^ Notably, fully recharged TAQ has slightly expanded lattice dimensions (575.8 Å^3^; see cryo-EM analysis in Supporting Information and Figure S30) relative to pristine TAQ (540.3 Å^3^), suggesting that initial lithiation of TAQ and delithiation of Li_4_TAQ induce a slight rearrangement of individual TAQ/TAQ^4–^ molecules, which subsequently support continuous cycling without further structural changes. Importantly, differences in the interlayer spacing between Li_4_TAQ and recharged TAQ suggest a maximal volume change of 6.0% under a high lithiation capacity of 300 mAh g^–1^_TAQ_, an important consideration for potential practical use.

### Benchmarking TAQ in Practically Relevant Metrics against Other Cathode Material Classes

**Figure 5:**
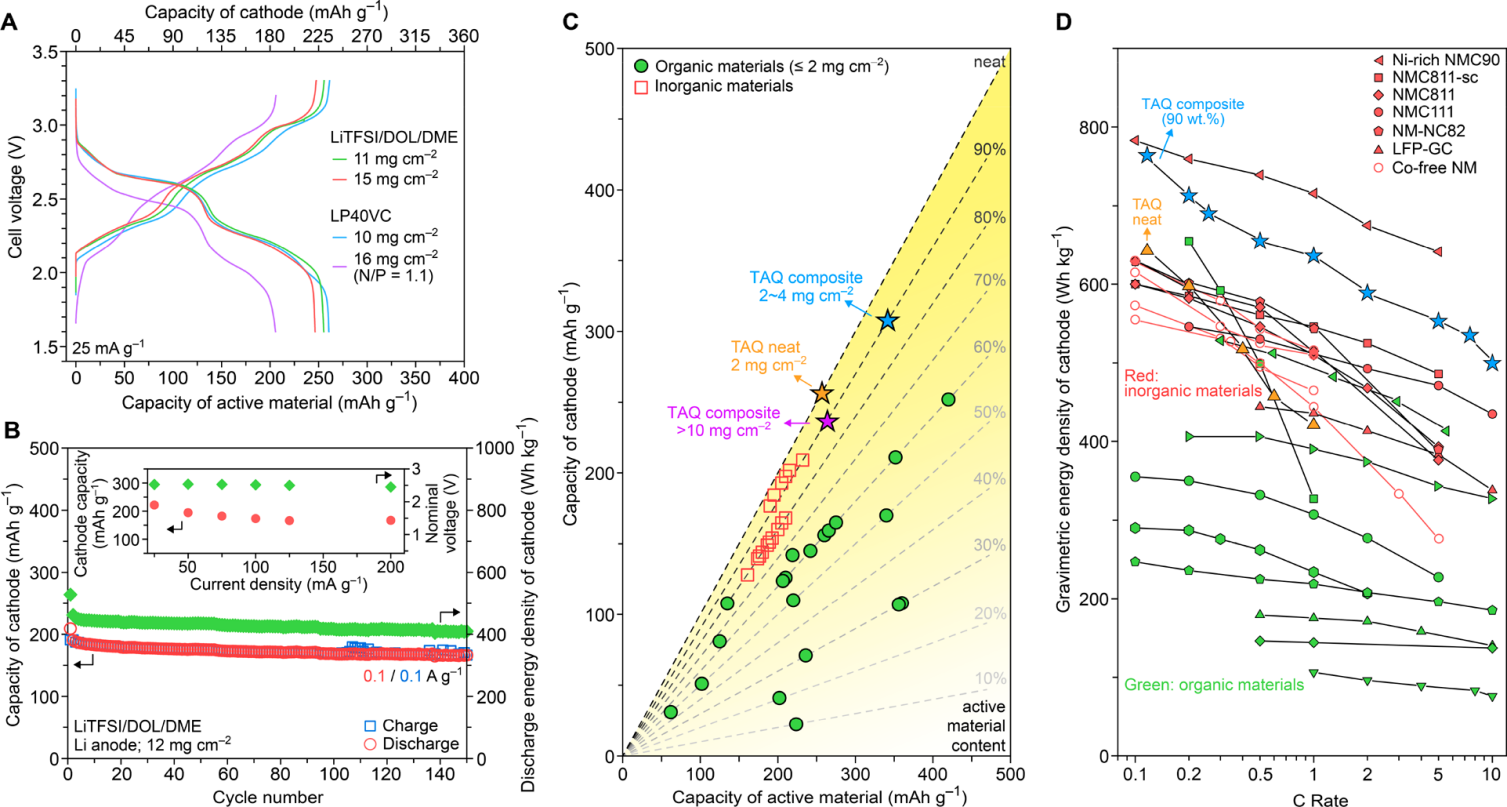
Benchmarking TAQ performance at high mass loading and against state-of-the-art cathodes. (A) GCD voltage profiles of TAQ/CMC/SBR composite electrodes at TAQ mass loadings over 10 mg cm^–2^. Li anodes were used except for the purple trace, collected with a GrLi anode. (B) Cycling of a TAQ/CMC/SBR composite electrode with a TAQ mass loading of 12 mg cm^–2^ at 0.1 A g^–1^. Inset shows the power rate performance of a TAQ/CMC/SBR composite electrode with a TAQ mass loading of 11 mg cm^–2^. (C) A comparison of active-material-based and electrode-based gravimetric specific capacities for various reported OEMs, TAQ, and inorganic cathodes. (D) Comparison of electrode-based gravimetric energy densities of various LIB cathode materials. Materials chosen in this comparison have an average discharge potential greater than 2 V vs Li^+^/Li. For each inorganic cathode material, data are selected from reports with the highest level of material optimization, either through electrode coatings, doping, or control on the crystalline domain size.^47−54^ For organic cathodes, the best performing materials are chosen from comprehensive recent reviews.^14−17^ A complete list of references can be found in the Supporting Information.

One of the major challenges for OEMs is the difficulty of achieving high areal mass loadings.^45^ OEMs have relatively low densities and thus require fabrication into thicker electrodes in order to achieve mass loadings that are comparable to those of inorganic cathodes. However, thicker electrodes made from intrinsically insulating common OEMs compound the problem of high ohmic resistances at practical-level mass loadings. As exposed above, TAQ is electrically conductive and virtually insoluble, but it also has high crystallographic density (for an OEM) of 1.9 g cm^–3^, which leads to a compaction density of ∼1.1 g cm^–3^ for TAQ cathodes and enables high mass loadings and areal capacities. Indeed, TAQ/CMC/SBR||Li cells with cathode mass loadings up to 15 mg cm^–2^ deliver cathode capacities of ∼230 mAh g^–1^ in both LP40VC and LiTFSI/DOL/DME (Figures 5A, S31A), corresponding to practically competitive areal capacities up to 3.52 mAh cm^–2^. Cycling electrodes with a mass loading of 12 mg cm^–2^ at a current density of 0.1 A g^–1^ (0.5C) and 100% DOD delivered a cathode capacity of 166 mAh g^–1^ after 150 cycles, with 87% capacity retention (Figure 5B). Moreover, increasing the rate from 25 to 200 mA g^–1^ delivered a consistent average discharge potential of 2.5 V and a 75% capacity retention (Figures 5B, S31B). Most relevantly, TAQ/CMC/SBR||GrLi full cells using LP40VC with TAQ mass loadings as high as 16 mg cm^–2^ and N/P ratios as low as 1.1 reached areal cathode capacities of 3.25 mAh cm^–2^ at 25 mA g^–1^ (Figures 5A, S31C,D), on par with the highest areal capacities reported previously for OEMs employing advanced electrode engineering.^16,46^

The electrode-level gravimetric specific capacities and energies of TAQ are compared with state-of-the-art inorganic and organic cathode materials in Figure 5C,D (material details and source data in Figure S32 and Tables S5 and S6). As discussed briefly earlier, typical OEMs are insulating and require large amounts (30–70 wt %) of conducting additives and binders, significantly above the commercial standard of 5–10 wt %. Thus, even though many OEMs exhibit high material-level metrics, their more practically relevant electrode-level metrics are modest. Here, because TAQ cells function with 90 wt % active material loading, TAQ-based cathodes store up to 306 mAh g^–1^ at 2–4 mg cm^–2^ and 240 mAh g^–1^ at >10 mg cm^–2^ active-material mass loadings (Figure 5C). In fact, although TAQ has a nominal voltage lower than that of common inorganic cathodes, electrode-level gravimetric specific energies for TAQ cathodes over a range of C rates outperform even optimized inorganic cathodes (Figures 5D, S32, S33). For instance, TAQ cathodes deliver at least 20–30% higher energy density, at the electrode level, than most of the composite cathodes based on single-crystalline NMC811 (NMC811-sc), commercial polycrystalline NMC811 and NMC111, or even state-of-the-art Co-free oxides, at rates from ∼0.1 C to 10 C.^47−54^ Notably, TAQ also delivers higher gravimetric energy density than graphite-coated LiFePO_4_ (LFP-GC) cathodes at charging rates that are at least 10 times faster.^48^ Equally importantly, TAQ electrodes deliver a higher volumetric energy density than comparable LFP electrodes (Table S7). TAQ thus presents measurable advantages relative to the leading contemporary LIB cathode technologies.

## Supporting Information Available

Experimental details, materials and electrochemical characterizations, supplementary texts, supplementary figures and tables (PDF)

## References

[ref1] Li W.; Erickson E. M.; Manthiram A. High-nickel layered oxide cathodes for lithium-based automotive batteries. Nat. Energy 2020, 5, 26–34. 10.1038/s41560-019-0513-0.

[ref2] Sovacool B. K. The precarious political economy of cobalt: balancing prosperity, poverty, and brutality in artisanal and industrial mining in the Democratic Republic of the Congo. Extr. Ind. Soc. 2019, 6, 915–939. 10.1016/j.exis.2019.05.018.

[ref3] Turcheniuk K.; Bondarev D.; Singhal V.; Yushin G. Ten years left to redesign lithium-ion batteries. Nature 2018, 559, 467–470. 10.1038/d41586-018-05752-3. 30046087

[ref4] Gent W. E.; Busse G. M.; House K. Z. The predicted persistence of cobalt in lithium-ion batteries. Nat. Energy 2022, 7, 1132–1143. 10.1038/s41560-022-01129-z.

[ref5] Lee S.; Manthiram A. Can cobalt be eliminated from lithium-ion batteries?. ACS Energy Lett. 2022, 7, 3058–3063. 10.1021/acsenergylett.2c01553.

[ref6] Zeng A.; Chen W.; Rasmussen K. D.; Zhu X.; Lundhaug M.; Müller D. B.; Tan J.; Keiding J. K.; Liu L.; Dai T.; Wang A.; Liu G. Battery technology and recycling alone will not save the electric mobility transition from future cobalt shortages. Nat. Commun. 2022, 13, 1341 10.1038/s41467-022-29022-z. 35292628 PMC8924274

[ref7] Padhi A. K.; Nanjundaswamy K. S.; Goodenough J. B. Phospho-olivines as positive-electrode materials for rechargeable lithium batteries. J. Electrochem. Soc. 1997, 144, 1188 10.1149/1.1837571.

[ref8] Wang Y.; Wang Y.; Hosono E.; Wang K.; Zhou H. The design of a LiFePO_4_/carbon nanocomposite with a core-shell structure and its synthesis by an in situ polymerization restriction method. Angew. Chem., Int. Ed. 2008, 47, 7461–7465. 10.1002/anie.200802539. 18720357

[ref9] Yuan L.-X.; Wang Z.-H.; Zhang W.-X.; Hu X.-L.; Chen J.-T.; Huang Y.-H.; Goodenough J. B. Development and challenges of LiFePO_4_ cathode material for lithium-ion batteries. Energy Environ. Sci. 2011, 4, 269–284. 10.1039/C0EE00029A.

[ref10] Cordell D.; Drangert J.-O.; White S. The story of phosphorus: Global food security and food for thought. Glob. Environ. Change 2009, 19, 292–305. 10.1016/j.gloenvcha.2008.10.009.

[ref11] Spears B. M.; Brownlie W. J.; Cordell D.; Hermann L.; Mogollón J. M. Concerns about global phosphorus demand for lithium-iron-phosphate batteries in the light electric vehicle sector. Commun. Mater. 2022, 3, 14 10.1038/s43246-022-00236-4.

[ref12] Tarascon J.-M. Key challenges in future Li-battery research. Philos. Trans. R. Soc. A 2010, 368, 3227–32411. 10.1098/rsta.2010.0112. 20566508

[ref13] Larcher D.; Tarascon J.-M. Towards greener and more sustainable batteries for electrical energy storage. Nat. Chem. 2015, 7, 19–29. 10.1038/nchem.2085. 25515886

[ref14] Poizot P.; Gaubicher J.; Renault S.; Dubois L.; Liang Y.; Yao Y. Opportunities and challenges for organic electrodes in electrochemical energy storage. Chem. Rev. 2020, 120, 6490–6557. 10.1021/acs.chemrev.9b00482. 32207919

[ref15] Lu Y.; Chen J. Prospects of organic electrode materials for practical lithium batteries. Nat. Rev. Chem. 2020, 4, 127–142. 10.1038/s41570-020-0160-9. 37128020

[ref16] Kim J.; Kim Y.; Yoo J.; Kwon G.; Ko Y.; Kang K. Organic batteries for a greener rechargeable world. Nat. Rev. Mater. 2023, 8, 54–70. 10.1038/s41578-022-00478-1.

[ref17] Lu Y.; Zhang Q.; Li L.; Niu Z.; Chen J. Design strategies toward enhancing the performance of organic electrode materials in metal-ion batteries. Chem. 2018, 4, 2786–2813. 10.1016/j.chempr.2018.09.005.

[ref18] Wang S.; Wang L.; Zhang K.; Zhu Z.; Tao Z.; Chen J. Organic Li_4_C_8_H_2_O_6_ nanosheets for lithium-ion batteries. Nano Lett. 2013, 13, 4404–4409. 10.1021/nl402239p. 23978244

[ref19] Kolek M.; Otteny F.; Schmidt P.; Mück-Lichtenfeld C.; Einholz C.; Becking J.; Schleicher E.; Winter M.; Bieker P.; Esser B. Ultra-high cycling stability of poly(vinylphenothiazine) as a battery cathode material resulting from π-π interactions. Energy Environ. Sci. 2017, 10, 2334–2341. 10.1039/C7EE01473B.

[ref20] Song Z.; Qian Y.; Liu X.; Zhang T.; Zhu Y.; Yu H.; Otani M.; Zhou H. A quinone-based oligomeric lithium salt for superior Li-organic batteries. Energy Environ. Sci. 2014, 7, 4077–4086. 10.1039/C4EE02575J.

[ref21] Luo Z.; Liu L.; Zhao Q.; Li F.; Chen J. An insoluble benzoquinone-based organic cathode for use in rechargeable lithium-ion batteries. Angew. Chem., Int. Ed. 2017, 56, 12561–12565. 10.1002/anie.201706604. 28787540

[ref22] Peng C.; Ning G.-H.; Su J.; Zhong G.; Tang W.; Tian B.; Su C.; Yu D.; Zu L.; Yang J.; Ng M.-F.; Hu Y.-S.; Yang Y.; Armand M.; Loh K. P. Reversible multi-electron redox chemistry of π-conjugated N-containing heteroaromatic molecule-based organic cathodes. Nat. Energy 2017, 2, 17074 10.1038/nenergy.2017.74.

[ref23] Liang Y.; Zhang P.; Chen J. Function-oriented design of conjugated carbonyl compound electrodes for high energy lithium batteries. Chem. Sci. 2013, 4, 1330–1337. 10.1039/c3sc22093a.

[ref24] Lee J.; Park M. J. Tattooing dye as a green electrode material for lithium batteries. Adv. Energy Mater. 2017, 7, 1602279 10.1002/aenm.201602279.

[ref25] Huang W.; Zhu Z.; Wang L.; Wang S.; Li H.; Tao Z.; Shi J.; Guan L.; Chen J. Quasi-solid-state rechargeable lithium-ion batteries with a calix[4]quinone cathode and gel polymer electrolyte. Angew. Chem., Int. Ed. 2013, 52, 9162–9166. 10.1002/anie.201302586. 23825051

[ref26] Chen T.; Banda H.; Yang L.; Li J.; Zhang Y.; Parenti R.; Dincǎ M. High-rate, high-capacity electrochemical energy storage in hydrogen-bonded fused aromatics. Joule 2023, 7, 986–1002. 10.1016/j.joule.2023.03.011.

[ref27] Lin Z.; Liu T.; Ai X.; Liang C. Aligning academia and industry for unified battery performance metrics. Nat. Commun. 2018, 9, 5262 10.1038/s41467-018-07599-8. 30531912 PMC6288112

[ref28] Li Z.; Jia Q.; Chen Y.; Fan K.; Zhang C.; Zhang G.; Xu M.; Mao M.; Ma J.; Hu W.; Wang C. A small molecular symmetric all-organic lithium-ion battery. Angew. Chem., Int. Ed. 2022, 61, e202207221 10.1002/anie.202207221. 35641442

[ref29] Lee S.; Hong J.; Jung S.-K.; Ku K.; Kwon G.; Seong W. M.; Kim H.; Yoon G.; Kang I.; Hong K.; Jang H. W.; Kang K. Charge-transfer complexes for high-power organic rechargeable batteries. Energy Stor. Mater. 2019, 20, 462–469. 10.1016/j.ensm.2019.05.001.

[ref30] Feng J. K.; Cao Y. L.; Ai X. P.; Yang H. X. Polytriphenylamine: A high power and high capacity cathode material for rechargeable lithium batteries. J. Power Sources 2008, 177, 199–204. 10.1016/j.jpowsour.2007.10.086.

[ref31] Jia H.; Quan T.; Liu X.; Bai L.; Wang J.; Boujioui F.; Ye R.; Vlad A.; Lu Y.; Gohy J.-F. Core-shell nanostructured organic redox polymer cathodes with superior performance. Nano Energy 2019, 64, 103949 10.1016/j.nanoen.2019.103949.

[ref32] Brédas J. L.; Scott J. C.; Yakushi K.; Street G. B. Polarons and bipolarons in polypyrrole: Evolution of the band structure and optical spectrum upon doping. Phys. Rev. B 1984, 30, 1023–1025. 10.1103/PhysRevB.30.1023.

[ref33] Petit P.; Jouguelet E.; Fischer J. E.; Rinzler A. G.; Smalley R. E. Electron spin resonance and microwave resistivity of single-wall carbon nanotubes. Phys. Rev. B 1997, 56, 9275–9278. 10.1103/PhysRevB.56.9275.

[ref34] Tukamoto H.; West A. R. Electronic conductivity of LiCoO_2_ and its enhancement by magnesium doping. J. Electrochem. Soc. 1997, 144, 3164–3168. 10.1149/1.1837976.

[ref35] Amin R.; Chiang Y.-M. Characterization of electronic and ionic transport in Li_1-*x*_Ni_0.33_Mn_0.33_Co_0.33_O_2_ (NMC333) and Li_1-*x*_Ni_0.50_Mn_0.20_Co_0.30_O_2_ (NMC523) as a function of Li content. J. Electrochem. Soc. 2016, 163, A1512–A1517. 10.1149/2.0131608jes.

[ref36] Xu Y.-N.; Chung S.-Y.; Bloking J. T.; Chiang Y.-M.; Ching W. Y. Electronic structure and electrical conductivity of undoped LiFePO_4_. Electrochem. Solid-State Lett. 2004, 7, A131–A134. 10.1149/1.1703470.

[ref37] Kasnatscheew J.; Evertz M.; Streipert B.; Wagner R.; Klöpsch R.; Vortmann B.; Hahn H.; Nowak S.; Amereller M.; Gentschev A.-C.; Lamp P.; Winter M. The truth about the 1st cycle Coulombic efficiency of LiNi_1/3_Co_1/3_Mn_1/3_O_2_ (NCM) cathodes. Phys. Chem. Chem. Phys. 2016, 18, 3956–3965. 10.1039/C5CP07718D. 26771035

[ref38] Lee J.-H.; Lee S.; Paik U.; Choi Y.-M. Aqueous processing of natural graphite particulates for lithium-ion battery anodes and their electrochemical performance. J. Power Sources 2005, 147, 249–255. 10.1016/j.jpowsour.2005.01.022.

[ref39] Aurbach D.; Gamolsky K.; Markovsky B.; Gofer Y.; Schmidt M.; Heider U. On the use of vinylene carbonate (VC) as an additive to electrolyte solutions for Li-ion batteries. Electrochim. Acta 2002, 47, 1423–1439. 10.1016/S0013-4686(01)00858-1.

[ref40] Barchasz C.; Leprêtre J.-C.; Patoux S.; Alloin F. Electrochemical properties of ether-based electrolytes for lithium/sulfur rechargeable batteries. Electrochim. Acta 2013, 89, 737–743. 10.1016/j.electacta.2012.11.001.

[ref41] Zhang K.; Guo C.; Zhao Q.; Niu Z.; Chen J. High-performance organic lithium batteries with an ether-based electrolyte and 9,10-anthraquinone (AQ)/CMK-3 cathode. Adv. Sci. 2015, 2, 1500018 10.1002/advs.201500018. PMC5115363 27980937

[ref42] Zhao Q.; Wang J.; Chen C.; Ma T.; Chen J. Nanostructured organic electrode materials grown on graphene with covalent-bond interaction for high-rate and ultra-long-life lithium-ion batteries. Nano Res. 2017, 10, 4245–4255. 10.1007/s12274-017-1580-9.

[ref43] Liu H.; Strobridge F. C.; Borkiewicz O. J.; Wiaderek K. M.; Chapman K. W.; Chupas P. J.; Grey C. P. Capturing metastable structures during high-rate cycling of LiFePO_4_ nanoparticle electrodes. Science 2014, 344, 1252817 10.1126/science.1252817. 24970091

[ref44] Ravnsbæk D. B.; Xiang K.; Xing W.; Borkiewicz O. J.; Wiaderek K. M.; Gionet P.; Chapman K. W.; Chupas P. J.; Chiang Y.-M. Extended solid solutions and coherent transformations in nanoscale olivine cathodes. Nano Lett. 2014, 14, 1484–1491. 10.1021/nl404679t. 24548146

[ref45] Ue M.; Sakaushi K.; Uosaki K. Basic knowledge in battery research bridging the gap between academia and industry. Mater. Horiz. 2020, 7, 1937–1954. 10.1039/D0MH00067A.

[ref46] Molina A.; Patil N.; Ventosa E.; Liras M.; Palma J.; Marcilla R. Electrode engineering of redox-active conjugated microporous polymers for ultra-high areal capacity organic batteries. ACS Energy Lett. 2020, 5, 2945–2953. 10.1021/acsenergylett.0c01577.

[ref47] Moiseev I. A.; Savina A. A.; Pavlova A. D.; Abakumova T. A.; Gorshkov V. S.; Pazhetnov E. M.; Abakumov A. M. Single crystal Ni-rich NMC cathode materials for lithium-ion batteries with ultra-high volumetric energy density. Energy Adv. 2022, 1, 677–681. 10.1039/D2YA00211F.

[ref48] Song J.; Sun B.; Liu H.; Ma Z.; Chen Z.; Shao G.; Wang G. Enhancement of the rate capability of LiFePO_4_ by a new highly graphitic carbon-coating method. ACS Appl. Mater. Interfaces 2016, 8, 15225–15231. 10.1021/acsami.6b02567. 27238368

[ref49] Park G. T.; Namkoong B.; Kim S. B.; Liu J.; Yoon C. S.; Sun Y. K. Introducing high-valence elements into cobalt-free layered cathodes for practical lithium-ion batteries. Nat. Energy 2022, 7, 946–954. 10.1038/s41560-022-01106-6.

[ref50] Ren D.; Padgett E.; Yang Y.; Shen L.; Shen Y.; Levin B. D. A.; Yu Y.; DiSalvo F. J.; Muller D. A.; Abruña H. D. Ultrahigh rate performance of a robust lithium nickel manganese cobalt oxide cathode with preferentially orientated Li-diffusing channels. ACS Appl. Mater. Interfaces 2019, 11, 41178–41187. 10.1021/acsami.9b05602. 31600433

[ref51] Liu T.; Yu L.; Lu J.; Zhou T.; Huang X.; Cai Z.; Dai A.; Gim J.; Ren Y.; Xiao X.; Holt M. V.; Chu Y. S.; Arslan I.; Wen J.; Amine K. Rational design of mechanically robust Ni-rich cathode materials via concentration gradient strategy. Nat. Commun. 2021, 12, 6024 10.1038/s41467-021-26290-z. 34654811 PMC8520018

[ref52] Zhang R.; Wang C.; Zou P.; Lin R.; Ma L.; Li T.; Hwang I. H.; Xu W.; Sun C.; Trask S.; Xin H. L. Long-life lithium-ion batteries realized by low-Ni, Co-free cathode chemistry. Nat. Energy 2023, 8, 695–702. 10.1038/s41560-023-01267-y.

[ref53] Liu T.; Yu L.; Liu J.; Lu J.; Bi X.; Dai A.; Li M.; Li M.; Hu Z.; Ma L.; Luo D.; Zheng J.; Wu T.; Ren Y.; Wen J.; Pan F.; Amine K. Understanding Co roles towards developing Co-free Ni-rich cathodes for rechargeable batteries. Nat. Energy 2021, 6, 277–286. 10.1038/s41560-021-00776-y.

[ref54] de Biasi L.; Kondrakov A. O.; Geßwein H.; Brezesinski T.; Hartmann P.; Janek J. Between scylla and charybdis: balancing among structural stability and energy density of layered NCM cathode materials for advanced lithium-ion batteries. J. Phys. Chem. C 2017, 121, 26163–26171. 10.1021/acs.jpcc.7b06363.

