# Supplementary material for: A Layered Organic Cathode for High-Energy, Fast-Charging, and Long-Lasting Li-Ion Batteries
Source: ACS Cent Sci. 2024 Jan 18;10(3):569–78. doi: 10.1021/acscentsci.3c01478 (PMC10979494; doi:10.1021/acscentsci.3c01478)
Supplement: Supplementary file 1 — oc3c01478_si_001.pdf [file oc3c01478_si_001.pdf]

Supporting Information for

A Layered Organic Cathode for High-Energy, Fast-Charging, and Long-Lasting Li-Ion Batteries

Tianyang Chen[1]‡, Harish Banda[1]‡, Jiande Wang[1], Julius J. Oppenheim[1], Alessandro Franceschi[2], Mircea Dincă[1]*

[1]Department of Chemistry, Massachusetts Institute of Technology, Cambridge, MA 02139, United States
[2]Department of Industrial Engineering, University of Bologna, Bologna 40136, Italy

‡These authors contributed equally to this work

Corresponding author email: mdinca@mit.edu

The PDF file includes:

Materials and Methods
Supplementary Text
Figures S1 to S33
Tables S1 to S7
References

Materials and Methods

Materials

All commercially available chemicals were used without further purification unless otherwise noted. Potassium phthalimide (98%, Sigma-Aldrich), acetonitrile (HPLC grade, Sigma-Aldrich), dimethylformamide (HPLC grade, VWR Chemicals BDH), hydrazine hydrate (Sigma-Aldrich), tetrabutylammonium chloride (99%, Alfa-Aesar), tetrabutylammonium bromide (98%, Sigma-Aldrich), methanol (VWR Chemicals BDH), ethanol (KOPTEC 200 proof), LP30 (GOTION), LP40 (GOTION), LiTFSI (Battery grade, DoDoChem), DOL (Battery grade, DoDoChem), DME (Battery grade, DoDoChem), vinylene carbonate (Battery grade, Sigma-Aldrich). All yields refer to isolated yields. House DI water and house nitrogen were used during the synthesis. NMR solvents were purchased from Cambridge Isotope Laboratory.

Methods

Synthetic details

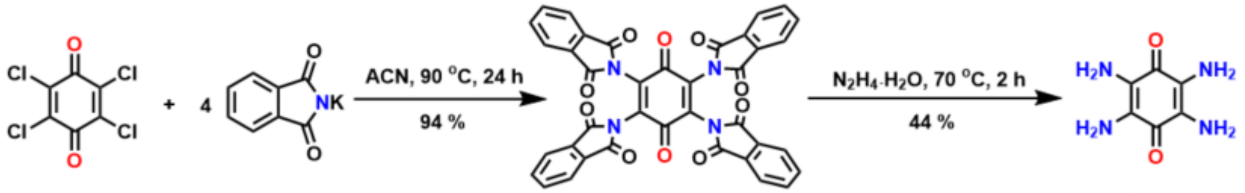

The synthesis of TABQ was adapted from previous literature report.[1]

Tetra(phthalimido)-p-benzoquinone (TPBQ)

To a stirred solution of tetrachloro-*p*-benzoquinone (100 g, Sigma-Aldrich) and 1 L acetonitrile (ACN) in a 2-L round-bottom flask, potassium phthalimide (300 g) was added. The reaction mixture was heated at 90 °C and refluxing for 24 h under N_2 atmosphere, after which the reaction mixture was cooled down to room temperature and filtered. The resulting solid was washed with 1 L of acetonitrile and then suspended in 400 mL of DMF. The dark colored suspension was heated at 100 °C and stirred for 30 min, which was followed by hot filtration to obtain pale brown solid with a yield of 267 g (94%). TPBQ is insoluble in all common NMR solvents. ATR-FTIR (cm^{-1}): 3105, 3074, 3051, 1790, 1740, 1688, 1628, 1609, 1468, 1375, 1360, 1244, 1177, 1153, 1099, 1080, 1011, 964, 876, 793, 777, 741, 710, 665.

Tetraamino-p-benzoquinone (TABQ)

To a 1-L round-bottom flask was added 70 g of TPBQ powder, followed by the addition of 250 mL of hydrazine monohydrate. The reaction mixture was heated at 70 °C for 2 hours, after which it was cooled to room temperature, filtered, and washed with DI water until the filtrate coming out is colorless. The resulting deep purple crystalline solid (7.5 g, 43.9%) was deemed to be pure TABQ by NMR spectroscopy. ^{1}H-NMR (400 MHz, DMSO-d6): 4.54 ppm; ^{13}C-NMR (100 MHz, DMSO-d6): 178.85, 121.37 ppm.

Bis-tetraaminobenzoquinone (TAQ)

TAQ is synthesized using a solvothermal method. To a 250 mL pressure tube with a PTFE cap was added 1.07 g of TABQ, 6.8 g of tetrabutylammonium chloride (99%, Alfa Aesar), and 60 mL of DMF. The tube was capped under ambient conditions, sonicated for 1 min, and heated in an isothermal oven at 120 °C for 12 hours, after which it was cooled down to room temperature. Then the reaction mixture was filtered and washed with DMF until the filtrate coming out became colorless. The resulting dark-greenish black solid was further washed with methanol and dried

under vacuum yielding 0.32 g of TAQ (33% yield). TAQ is poorly soluble in common NMR solvents. Solid-state NMR of TAQ: 169.3 ppm (*C*=O), 140.8 ppm (*C*=N), 129.1 ppm/124.5 ppm (*C*–N/*C*–O). Elemental analysis: $C_{12}H_{10}O_4N_6$, found: C, 47.45%; H, 3.25%; N, 27.08%; calculated: C, 47.69%; H, 3.33%; N, 27.81%. MALDI-TOF: $[TAQ]^+$: found, 302.077; calculated: 302.076. FTIR (cm^{-1}, Figure S14): 3470, 3404, 3356, 3298, 3235, 1651, 1626, 1614, 1568, 1543, 1462, 1364, 1244, 1186, 1136, 721, 654. The crystal structure and phase purity of TAQ were characterized by the WAXS pattern (Figure S1A).

<u>Electrode fabrication and electrochemical measurements</u>

Electrochemical performances of TAQ electrodes are tested in CR2032 coin cells assembled in an Ar-filled glove box. All electrodes are prepared under ambient conditions. Neat TAQ electrodes (i.e., 100% active material content) are made by coating well-mixed (upon sonication for at least 10 min) slurry of TAQ in ethanol (~10 mg/mL) on stainless steel disks or carbon-coated copper current collectors for high-rate tests. Neat TAQ electrodes with high mass loadings are pressed using a metal rod to ensure the adhesion to the current collector and the compaction of the electrode. TAQ composite electrodes contain 90 wt.% of TAQ, 5 wt.% of carbon black (CB) and 5 wt.% of binder (carboxymethyl cellulose (CMC) and styrene butadiene rubber (SBR) with various ratios). CMC and SBR are well dissolved/suspended in small amount of water (50~100 μL/mg) upon heating at 85 °C with vigorous stirring for an hour. Well-mixed TAQ and CB powders (upon grinding) are transferred into the CMC/SBR aqueous mixture, and the resulting mixture is vigorously stirred at 85 °C for an hour to produce the cathode slurry, which is then coated on stainless steel disks. All electrodes are dried under ambient condition before drying in a vacuum oven at 100 °C for 12 hours. Neat TAQ electrodes have areal mass loadings between 1.5 and 3.0 mg cm^{-2}, and TAQ composite electrodes have areal active material mass loadings between 2.0 and 4.0 mg cm^{-2}. High mass loading studies used electrodes containing 10 – 16 mg cm^{-2} active material. Extra caution is suggested when dealing with neat TAQ electrodes compared to the TAQ composite electrodes. Half cells use a lithium chip as the anode and a glass fiber separator. Full cells are assembled using TAQ cathode and pre-lithiated graphite anodes. To fabricate pre-lithiated graphite electrodes, commercial graphite anode on copper foil (MTI Corporation, 8 mg cm^{-2}) is cut into 15-mm disks (~14 mg of graphite per disk), and the resulting electrodes are electrochemically lithiated in graphite||Li cells by constant current discharge at a current of 0.2 mA. Commercial electrolytes including 1 M $LiPF_6$ in 1:1 ethylene carbonate/dimethyl carbonate (LP30), LP30 with 5 wt.% of vinylene carbonate (VC), 1 M $LiPF_6$ in 1:1 ethylene carbonate/diethyl carbonate (LP 40), LP40 with 5 wt.% of VC, and 1 M LiTFSI in 1:1 1,3-dioxolane/dimethoxyethane are used for battery tests. Batteries are typically tested between 1.6 V and 3.2 V versus Li^+/Li using Landt battery cyclers at room temperature (25 °C). Electrodes with high mass loadings are tested between 1.6 V and 3.3 V versus Li^+/Li. High rate cycling (5C or higher; 1C = 200 mA g^{-1}) are tested between 1.5 V and 3.2 V. Galvanostatic Intermittent Titration Technique (GITT), cyclic voltammetry, and electrochemical impedance spectroscopy (EIS) tests are performed using a BioLogic VMP-3 workstation. GITT measurements are carried out at 0.1C where each step consists of a one-hour constant-current discharge or charge followed by a two-hour rest. GITT data analysis details are described in Supplementary Information. EIS measurements were performed using a multi-sinusoidal signal with an amplitude of 10 mV over a large frequency range from 10 mHz to 1 MHz at various potentials versus Li^+/Li. The EIS data were fitted by the *ZFit* tool in *ECLab*.

Characterization methods

Powder X-ray diffraction (PXRD)

Laboratory PXRD patterns were recorded using a Bruker Advance II diffractometer equipped with a θ/2θ reflection geometry and Ni-filtered Cu Kα radiation ($K\alpha_1$ = 1.5406 Å, $K\alpha_2$ = 1.5444 Å, $K\alpha_2$/ $K\alpha_1$ = 0.5). The tube voltage and current were 40 kV and 40 mA, respectively. Samples for PXRD were prepared by placing a thin layer of the appropriate material on a zero-background silicon crystal plate.

Wide-angle X-ray scattering (WAXS) measurements

WAXS measurements were conducted at the Soft Matter Interfaces beamline (12-ID) of the National Synchrotron Light Source II (NSLS-II) at Brookhaven National Laboratory with a beam energy of 16.1 keV and beam size of 200 × 20 μm. The Scattered data were collected in vacuum with a PILATUS3 300 kW detector (Dectris, Switzerland), consisting of 0.172 mm square pixels in a 1475 × 195 array. To obtain a wide range of wave vector transfer (q), a series of 2D diffraction patterns were collected by rotating the detector on an arc with the sample-to-detector distance being 275 mm. Scattering patterns from each detector angle were stitched together using custom software and then reduced to 1D scattering intensity versus q curve by circular average.

In-operando PXRD

The in-operando PXRD measurements are performed using custom-built coin cells (Figure S28) on a Panalytical Empyrean diffractometer equipped with a Mo X-ray source, focusing mirror and Galipix 3D detector.

Elemental analyses

Elemental analyses were performed by Robertson Microlit Laboratories, Ledgewood, New Jersey.

Matrix assisted laser desorption ionization (MALDI) time-of-flight (TOF) mass spectrometry

MALDI-TOF mass spectrum was conducted using a high-resolution Bruker Autoflex LRF Speed mass spectrometer in the positive linear mode. Measurement parameters were optimized for mass range 0–500 Da. Dithranol (m/z = 226.063 Da for $[M]^+$) was used as the Matrix and for the calibration of the instrument. TAQ powders were mixed with dithranol using mortar and pestle, and the mixture was pressed into a pellet for the measurement.

Scanning electron microscopy (SEM)

SEM images were collected at MIT MRSEC (formerly the Center for Materials Science and Engineering, or CMSE) on a Zeiss Merlin high-resolution scanning electron microscope with an InLens detector at an operating voltage of 3 or 4 kV.

High resolution cryogenic transmission electron microscopy (Cryo-EM)

High-resolution Cryo-EM images were obtained at the Automated Cryogenic Electron Microscopy Facility in MIT.nano on a Talos Arctica G2 microscope operated at an accelerating voltage of 200 kV with a Falcon3EC direct electron detector. Pristine samples were prepared by sonicating TAQ powders in isopropanol for 5 to 10 seconds. To prepare the Cryo-EM specimens of cycled electrodes, the coin cells are disassembled, and the electrode materials are scraped off current collectors, washed with dry tetrahydrofuran (THF) and dried in an Ar-filled glove box. The resulting electrode materials are ultrasonicated, and dispersed in THF, and the suspension is dropcasted onto C-flat™ Cu grids with holey carbon in the glovebox. The TEM measurement conditions were adapted from our previous report (*26*). All image acquisition was done using EPU at an exposure time of 1 s, with focusing done adjacent to the region imaged to minimize beam

exposure prior to image acquisition (standard low dose imaging protocols). Analysis of the raw HRTEM data was done using Digital Micrograph (Gatan Microscopy Suite software).

Diffuse reflectance infrared Fourier transform spectroscopy (DRIFTS)

DRIFTS measurements were performed on a Bruker Tensor 37 (MIR source and KBr beam splitter) with a mercury cadmium telluride (MCT, cooled with LN_2) detector utilizing the DiffusIR™ accessory (Pike Technologies). To ensure air-free measurement, a sealable environmental chamber equipped with ZnSe window (Pike Technologies) was used. Samples were ground in air (or under Ar for ex-situ measurements) with dry KBr in a mortar and pestle (99.9%, Pike technologies) to produce solid mixtures (0.5-1% wt.). The data was averaged over 128 scans between 4000 – 600 cm^{-1} with the resolution of 4 cm^{-1}. Each of the Kubelka-Munk function (F(R)) transformed DRIFTS spectra were normalized with respect to the DRUV–vis–NIR data by matching the F(R) values at 4000 cm^{-1}.

Diffuse reflectance UV-vis-NIR spectroscopy (DRUV–vis–NIR)

DRUV-Vis-NIR spectra were collected on a Cary 5000i spectrophotometer fitted with the UV-Vis DiffusIR accessory (Pike Technologies) between 200 and 2500 nm, at the scan rate of 600 nm/min under ambient conditions. A KBr baseline and a zero-background correction were collected prior to the sample measurements. Samples were prepared as described above for the DRIFTS measurements.

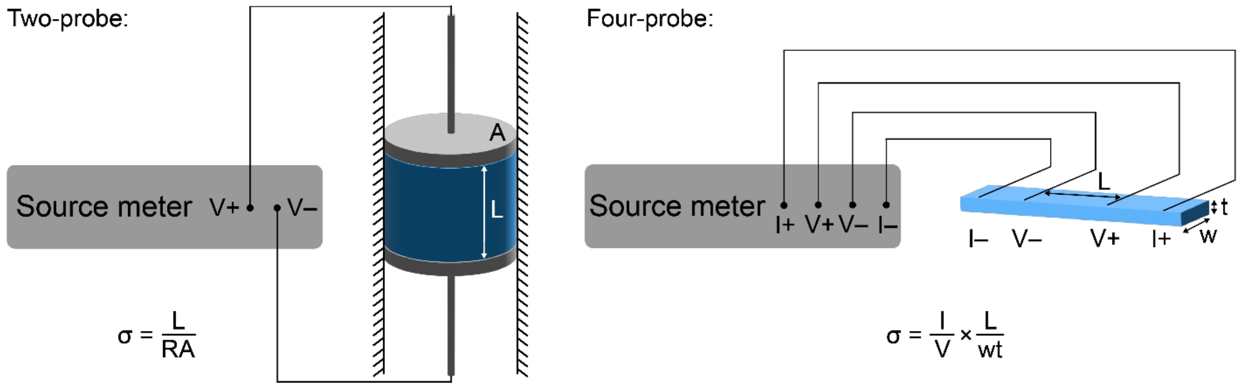

Room temperature electrical conductivity measurements

Electrical conductivities were measured at 296 K in ambient atmosphere on pressed pellets using a 2-probe or 4-probe setup described previously.[2] At least three pellets from different batches of each sample were measured. The thickness of the pellets is between 0.3 mm and 1 mm. For each sample, the conductivity values were averaged to give the average conductivity value.

Variable temperature electrical conductivity measurements

Temperature-dependent electrical conductivities were collected from 270 K to 390 K using a Quantum Design PPMS DynaCool instrument equipped with the Electrical Transport Option. A four-contact probe cell was fabricated for the measurements as described previously.[3] The temperature was varied at 5 K/min, and the resistance value was collected in AC mode for every 1 K. At least two heating-cooling cycles were performed. The phase angle remained within 1° throughout the entire temperature range of interest, which means that the difference of AC and DC resistance is below 1%.

Electron paramagnetic resonance spectroscopy (EPR)

EPR measurements were performed on samples packed under N_2 (for pristine TAQ) or under Ar (for discharged TAQ) in septum-sealed quartz tubes using a Bruker EMX spectrometer equipped with an ER 4199HS cavity and Gunn diode microwave source at room temperature, with a microwave frequency of ~9.37 GHz. The measurements were taken in perpendicular mode.

Pristine TAQ was measured in its neat form. For discharged TAQ samples, neat TAQ electrodes were first discharged to 1.6 V in the half-cell configuration using LP30 electrolyte. The electrode materials were then collected from current collectors, washed with DMC three times, and dried under vacuum. The resulting solid was mixed with KBr (spectroscopic grade) to give a diluted sample containing ~2.2 wt.% of discharged TAQ. The fitting/simulation of EPR signals were conducted using EasySpin.[4]

Magnetic property measurements

Magnetic data were collected using microcrystalline samples on a Quantum Design Dynacool D-209 Physical Property Measurement System (PPMS). Magnetization measurements were performed at 1.8 K in a field range of ±90 kOe. Susceptibility measurements were performed under 1 kOe external field in temperature range of 1.8–300 K. Experimental data were corrected for diamagnetic contribution based on blank sample holder measurements and Pascal's constants.

Computation

The calculations of both keto- and enol-form of TAQ, including HOMO/LUMO energies and vibrational spectroscopy, were conducted at B3LYP/ma-def2-QZVP or B3LYP/def2-TZVP level using ORCA.

X-ray photoelectron spectroscopy (XPS)

XPS measurements were performed at the MIT MRSEC using a Physical Electronics PHI Versaprobe II X-ray photoelectron spectrometer equipped with a monochromatic Al anode X-ray source. The main chamber pressure was in the 10^{-10} Torr range. Sample powders were pressed on copper tapes with full coverage. Survey spectra were collected from 0~1100 eV in binding energy (BE), with a resolution of 0.8 eV. High resolution spectra of C 1s, N 1s, O 1s, and Li 1s regions were collected with a resolution of 0.1 eV. BE calibration was carried out by shifting the adventitious carbon C1s peak to 284.8 eV.

Supplementary Text

Electronic and magnetic characterizations

For discharged TAQ samples, neat TAQ electrodes were first discharged to 1.6 V in coin cells using Li anode and LP30, and delivered an average discharge capacity of 268.3 mAh g^{-1} (~ 75% of theoretical capacity), corresponding to the 1:1 mixture of Li_2TAQ and Li_4TAQ (Li_3TAQ on average; MW = 323.07 g mol^{-1}). The voltage of discharged TAQ generally stabilizes between 1.90 V and 2.0 V once the discharge process is finished. A Dysonian lineshape was used to fit the EPR signal of pristine TAQ following the equation:[5]

$$\frac{d\chi}{dH} = I_0 \frac{\cos\varphi}{\Delta H^2} \left\{ \frac{-2\left[\frac{H-H_0}{\Delta H}\right] + \tan\varphi\left[1-\left(\frac{H-H_0}{\Delta H}\right)^2\right]}{\left[1+\left(\frac{H-H_0}{\Delta H}\right)^2\right]^2} \right\}$$

I_0 is the EPR signal intensity and is related to the spin susceptibility, H is the applied magnetic field, H_0 is the center field, and ΔH is the linewidth. The angle, φ, is indicative of the lineshape asymmetry and electron delocalization.

GITT analysis

During the GITT measurement, the electrode is completely wetted, so that the capacitive current measured arises entirely from the voltage sweep.

$$S \approx \frac{i}{C_m}\left(\frac{dE}{dt}\right)^{-1}$$

Where S is the real electrode electrolyte wetting area, C_m is the specific double layer capacitance measured at ±50 mV of the open circuit potential,[6] E is the voltage. The real electrode electrolyte wetting area S (cm^2) can thus be calculated.

The effective chemical lithium diffusion coefficient in the active material can be derived from the GITT potential response by following the method reported by Weppner and Huggins.[7] The chemical diffusion coefficient can be calculated at each step, with the following formula:

$$D = \frac{4}{\pi}\left(\frac{iV_m}{Z_A FS}\right)^2 \left[\left({dE}/{d\delta}\right) \Big/ \left({dE}/{d\sqrt{t}}\right)\right]^2$$

Here, i is the current (A); V_m is the molar volume of the electrode ($cm^3\ mol^{-1}$); Z_A is the charge number; F is the Faraday's constant (96485 C mol^{-1}); S is the electrode/electrolyte contact area or wetting area (cm^2); $dE/d\delta$ is the slope of the coulometric titration curve, found by plotting the steady state voltages E (V) measured after each titration step δ; $dE/d\sqrt{t}$ is the slope of the linearized plot of the potential E (V) during the current pulse of duration t (s).

If sufficient small currents are applied for short time intervals, so that $dE/d\sqrt{t}$ can be considered linear and the coulometric titration curve can be also considered linear over the composition range involved in that step, we can get:

$$D = \frac{4}{\pi\tau}\left(\frac{n_m V_m}{S}\right)^2 \left[\frac{\Delta E_s}{\Delta E_t}\right]^2 \qquad \left(\tau \ll {L^2}/{D}\right)$$

Here, τ is the duration of the current pulse (s); n_m is the number of moles (mol) of the active material; V_m is the molar volume of the electrode ($cm^3\ mol^{-1}$); S is the electrode/electrolyte contact area (cm^2); ΔE_s is the steady-state voltage change, due to the current pulse and ΔE_t is the voltage change during the constant current pulse, eliminating the iR drop. L is the film thickness. The figure below shows the examples for charge and discharge, where $\Delta E_s = E_4 - E_3$, $\Delta E_t = E_2 - E_1$.

The density of TAQ-based electrodes is around 1.1 g cm^{-3}, giving V_m = 274.6 $cm^3\ mol^{-1}$.

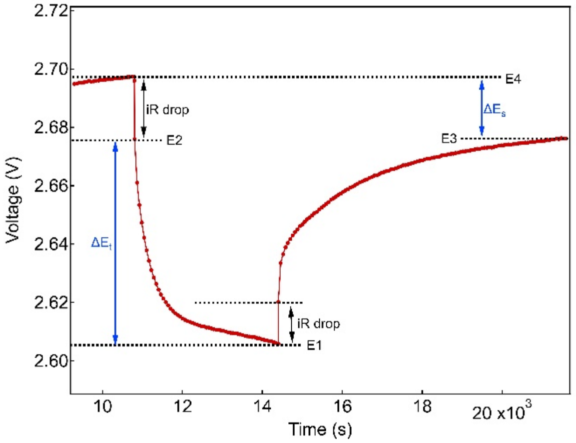

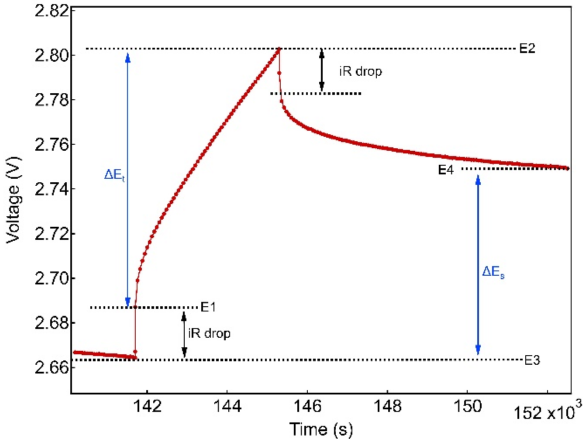

<u>In-operando powder X-ray diffraction</u>

The custom-made half-cells for in-operando PXRD measurements use a 10-µm-thick Al foil as the window (Figure S28). The corresponding electrodes are prepared as free-standing films, which is specifically designed for the in-operando measurements, by repeated kneading and rolling of a slurry of TAQ, acetylene black and a polytetrafluoroethylene (PTFE) solution mixture (6:3:1)

with ethanol. The prepared films are dried at 100 °C for at least 12 hours and pressed onto stainless steel mesh prior to use. To enhance the intensities of PXRD signals, TAQ mass loadings between 8 mg cm^{-2} and 10 mg cm^{-2} were used. The PXRD patterns were recorded in the Bragg-Brentano θ/2θ geometry using an in-operando coin cell battery holder. The coin cells were tested at a current density of 200 mA g^{-1} between 1.6 V and 3.3 V while PXRD patterns were recorded. To obtain enough intensity on this in-house diffractometer, 8~10 min of collection time is required for each PXRD pattern, which inherently limits the number of patterns collected during each charging/discharging cycle. Moreover, the 2θ range has to be set between 10° and 14° to obtain enough structural information. A larger 2θ range is not feasible due to the time limitation. Therefore, refinements of the in-situ PXRD patterns are not practical given such a narrow 2θ range.

Cryo-EM analysis

According to the Cryo-EM images of the cycled electrode and their Fast Fourier-Transform (FFT) patterns, the *d* spacings of (020), (002), (102), (011), and $(1\bar{1}0)$ planes are found to be 5.71 Å, 5.00 Å, 3.19 Å, 7.53 Å, and 4.53 Å, respectively. Therefore, referring to the equation for calculating the *d* spacing of (hkl) planes in Monoclinic crystal systems,

$$\frac{1}{d_{hkl}^2} = \frac{1}{\sin^2 \beta}\left(\frac{h^2}{a^2} + \frac{k^2 \sin^2 \beta}{b^2} + \frac{l^2}{c^2} - \frac{2hl \cos \beta}{ac}\right)$$

the estimated lattice parameters are obtained as:

$$\begin{cases} a = 5.05 \text{ Å} \\ b = 11.41 \text{ Å} \\ c = 10.23 \text{ Å} \\ \beta = 102.35° \end{cases}$$

These estimated lattice parameters lead to d_{102} and $d_{12\text{–}2}$ values of 3.186 Å and 3.253 Å, respectively, matching very well with the d_{102} (3.19 Å) and $d_{12\text{–}2}$ (3.25 Å) obtained from in-operando PXRD patterns.

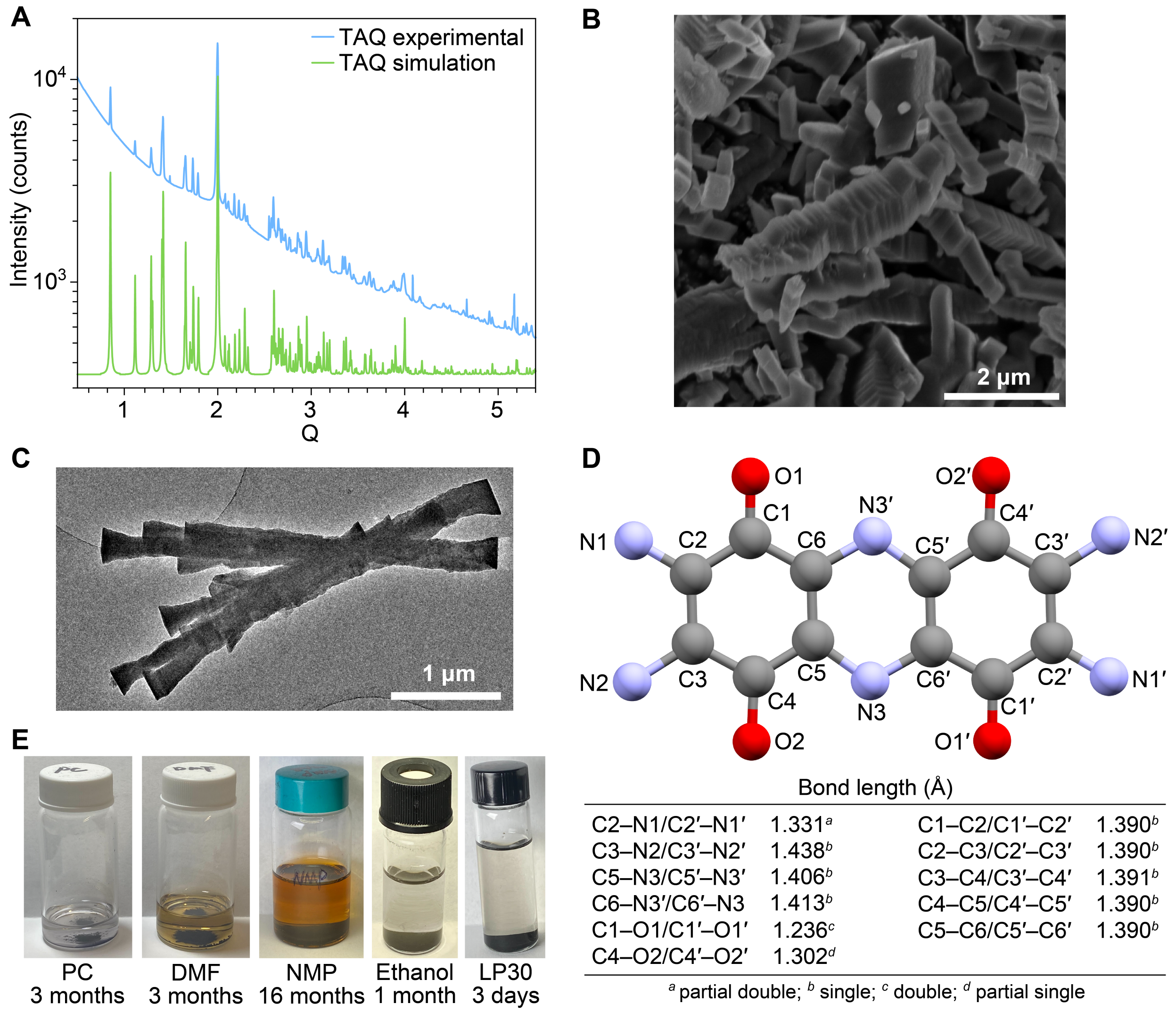

Bond length (Å)

C2–N1/C2′–N1′	1.331[a]	C1–C2/C1′–C2′	1.390[b]
C3–N2/C3′–N2′	1.438[b]	C2–C3/C2′–C3′	1.390[b]
C5–N3/C5′–N3′	1.406[b]	C3–C4/C3′–C4′	1.391[b]
C6–N3′/C6′–N3	1.413[b]	C4–C5/C4′–C5′	1.390[b]
C1–O1/C1′–O1′	1.236[c]	C5–C6/C5′–C6′	1.390[b]
C4–O2/C4′–O2′	1.302[d]		

[a] partial double; [b] single; [c] double; [d] partial single

Figure S1. Characterization of TAQ.

(A) Experimental and simulated WAXS patterns of TAQ. **(B)** A SEM image of TAQ. **(C)** Low-magnification Cryo-EM image of TAQ. **(D)** Bond lengths of TAQ obtained from the crystal structure. **(E)** Solubility tests of TAQ in common organic solvents and electrolytes.

Table S1.

Chemical and crystal structures of TAQ and organic molecules containing dihydropyrazine ring(s). Most of the molecules are flat. Average C–N bond lengths of dihydropyrazine rings are listed, which further supports that TAQ contains a dihydropyrazine ring.

ChemDraw Structures	Crystal Structures	Average C–N bond lengths†
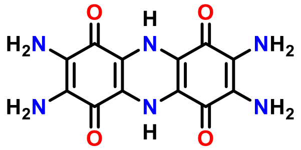 TAQ	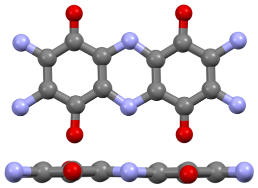 CCDC 2144223	1.410 Å
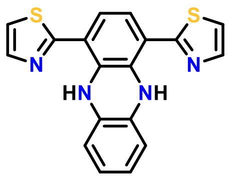	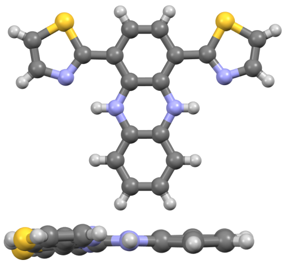 CCDC 2117191	1.384 Å
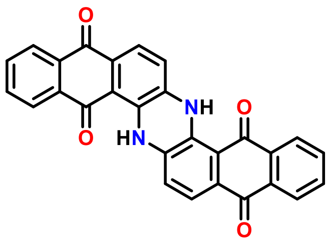	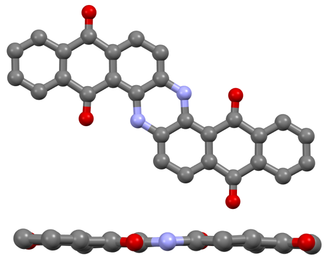 CCDC 1180353	1.388 Å
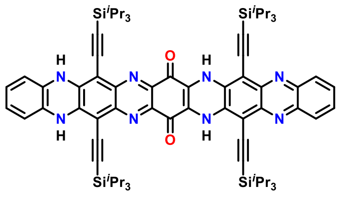	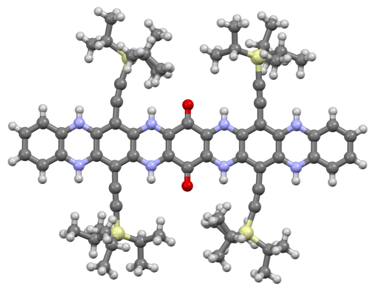	1.353 Å (average C–N distance of the dihydro-pyrazine and pyrazine rings)

	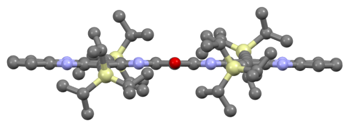 CCDC 1046845	
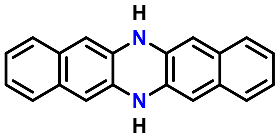	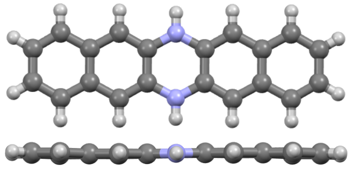 CCDC 220863/220864	1) 1.388 Å, 1.387 Å;<br>2) 1.389 Å.
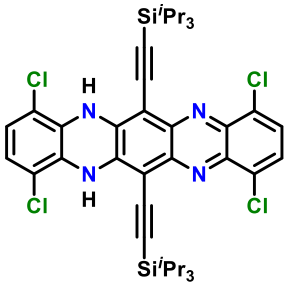	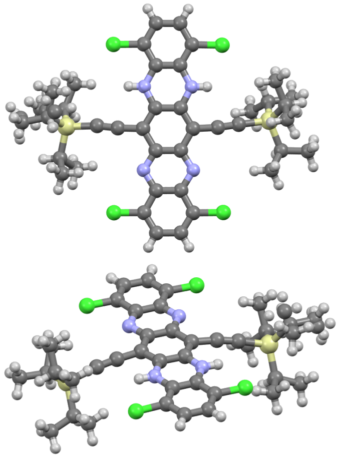 CCDC 1439302	1.381 Å;<br>1.347 Å (average C–N distance of the pyrazine ring).
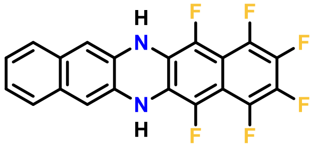	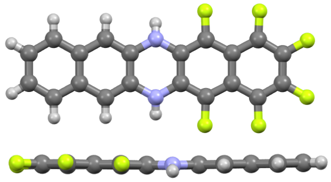 CCDC 1051996	1.386 Å
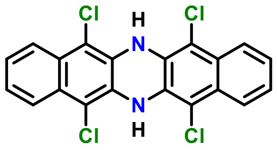	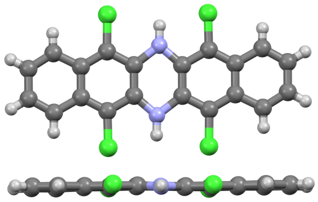 CCDC 726847	1.456 Å

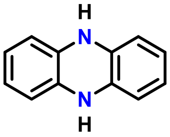 (*)	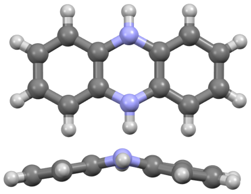 CCDC 148755	1.408 Å
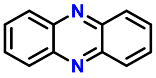 : 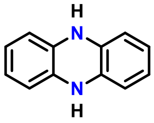 =1:1	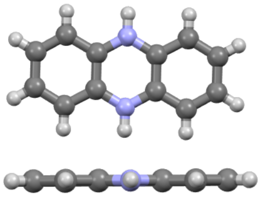 CCDC 148757	1.369 Å
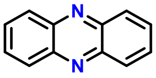 : 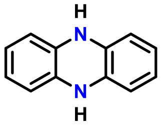 =3:1	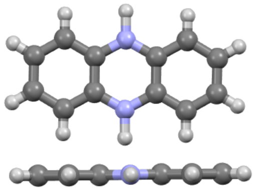 CCDC 148756	1.388 Å
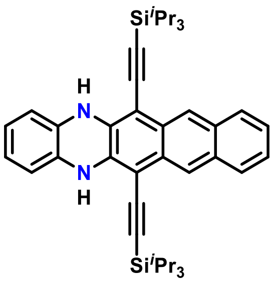	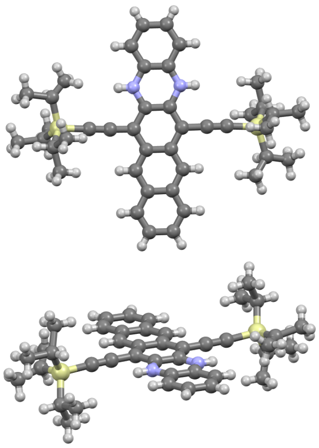 CCDC 793242	1.374 Å

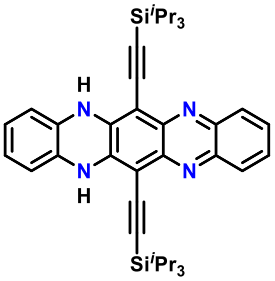

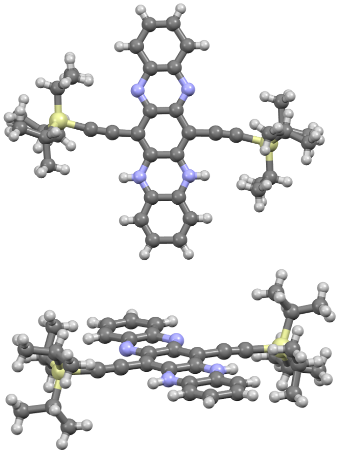

CCDC 793244

1.382 Å

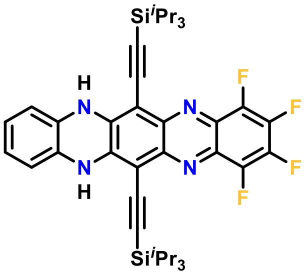

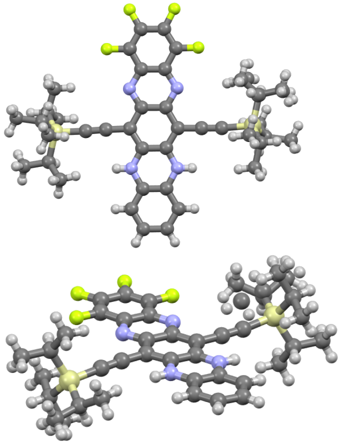

CCDC 956115

1.384 Å;
1.348 Å (average C–N distance of the pyrazine ring).

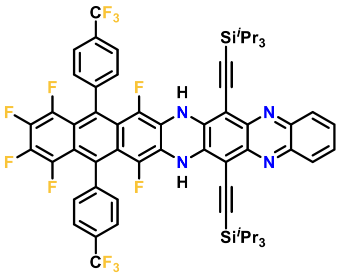

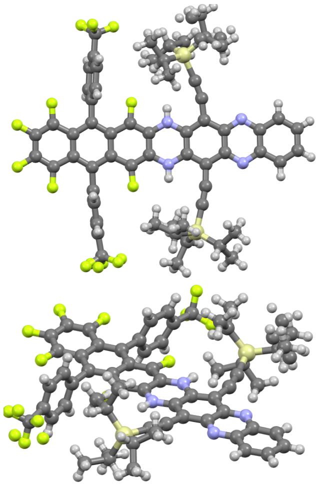

CCDC 956118

1.376 Å;
1.348 Å (average C–N distance of the pyrazine ring).

$[pzH_2]_3(OTf)_2$ (pz = phenazine)	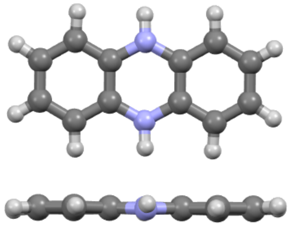 CCDC 1844902	1.391 Å (pzH_2); 1.375 Å ($[pzH_2]^{2+}$).

†Average bond length based on the C–N bonds of dihydropyrazine ring(s). *Dihedral angles: 150° ~ 160°.

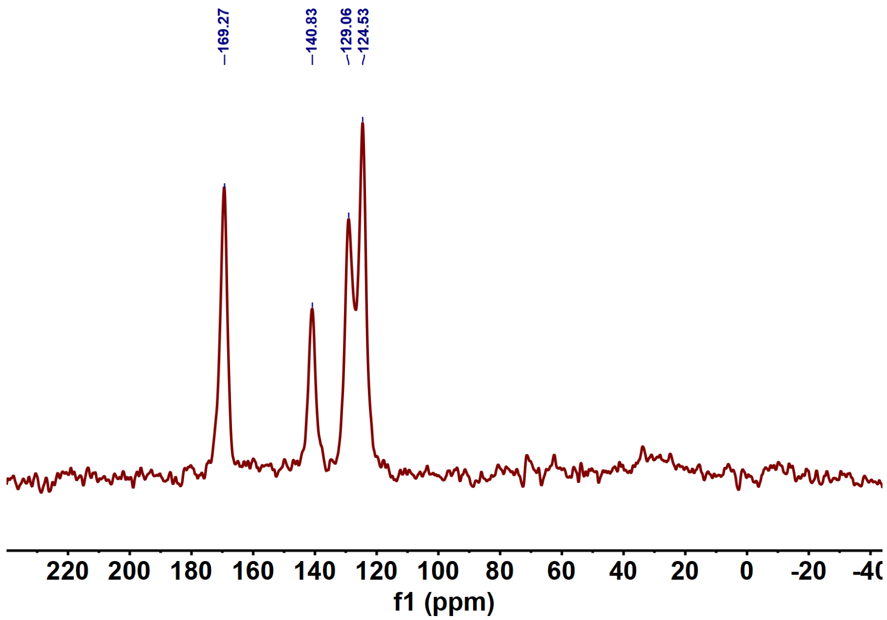

Figure S2. ^{13}C solid-state NMR spectrum of TAQ.

The peak at 140.83 ppm indicates the presence of C=NH, which is the result of keto-enol tautomerization of TAQ. The peak at 169.27 ppm belongs to carbonyl groups, which have slightly lower chemical shift relative to common carbonyl signals (> 170 ppm) due to the excellent electronic delocalization in TAQ molecules. The two peaks at 129.06 ppm and 124.53 ppm belong to C–N/C–O.

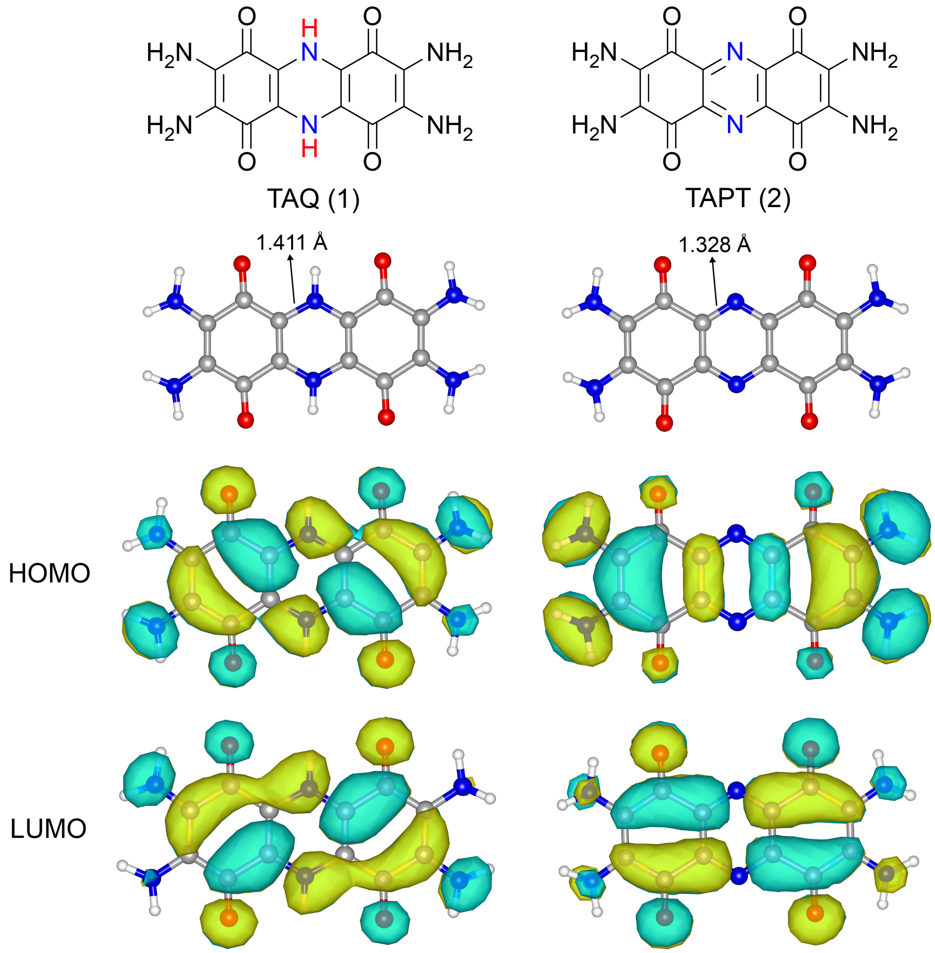

Figure S3. Molecular structure, and HOMO/LUMO orbital diagrams of TAQ (1) and tetraamino-phenazine-1,4,6,9-tetrone (2) optimized by DFT calculations.

The bond length of the marked C–N bond of TAQ matches well with the experimental determined bond length (CCDC 2144223/2263467, only *non-H* atoms have been experimentally located by continuous rotational electron diffraction), whereas that of **2** is significantly shorter than the experimental values and is within the range of C=N. The frontier orbitals of TAQ exhibit significantly better electronic delocalization compared with **2**, indicating better extended conjugation of TAQ.

Table S2.
HOMO and LUMO energies of both quinone and imine forms of TAQ (1) and tetraamino-phenazine-1,4,6,9-tetrone (2).

	B3LYP/ma-def2-QZVP			B3LYP/def2-TZVP		
	HOMO (eV)	LUMO (eV)	HOMO-LUMO gap (eV)	HOMO (eV)	LUMO (eV)	HOMO-LUMO gap (eV)
**1**	–4.9203	–3.4363	**1.484**	–4.4962	–3.2543	**1.2419**
**2**	–5.8621	–3.6328	**2.2293**	–5.7602	–3.5483	**2.2119**

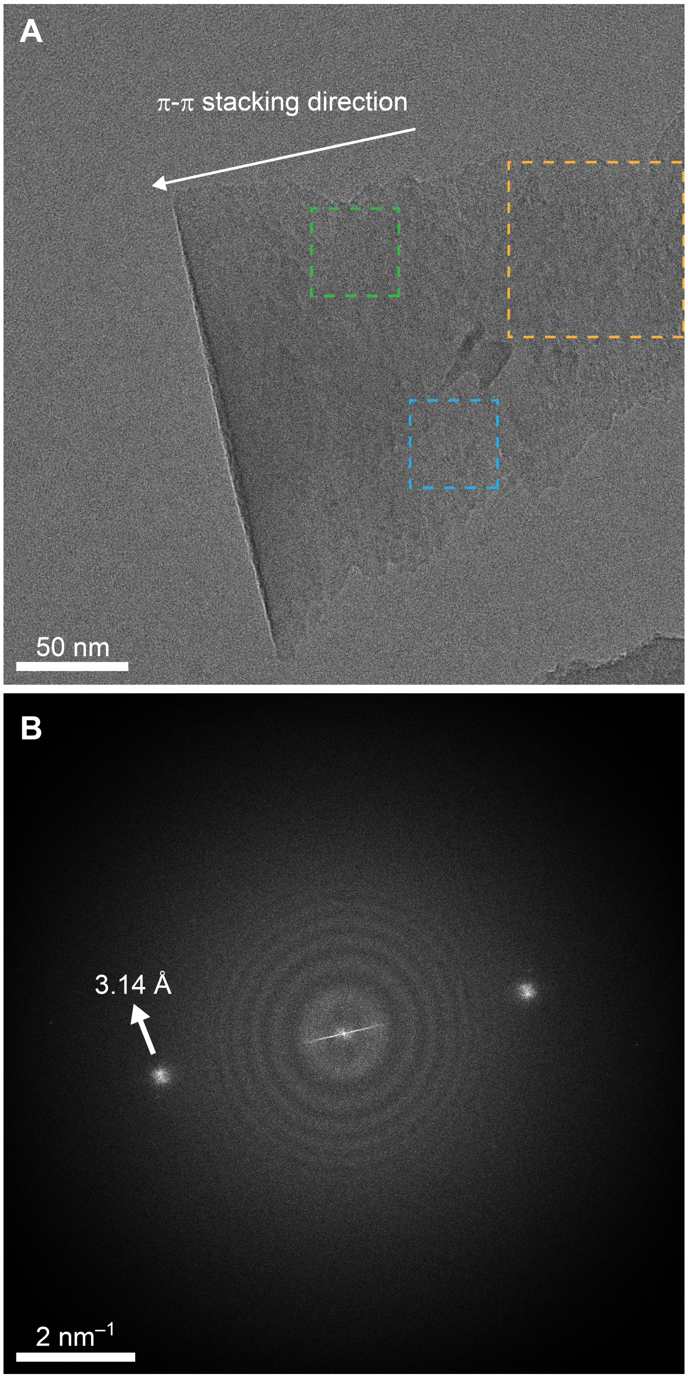

Figure S4. A Cryo-EM image of TAQ and its Fast-Fourier Transform (FFT).
The diffraction spots in **B** correspond to a *d* spacing of 3.14 Å, matching well with the interlayer distance of TAQ 2D layers. The π–π stacking direction of TAQ 2D layers is parallel to the long axis of this TAQ rod, as indicated by the white arrow in **A**. Higher magnification images of the highlighted dashed square areas are shown in Figure S5.

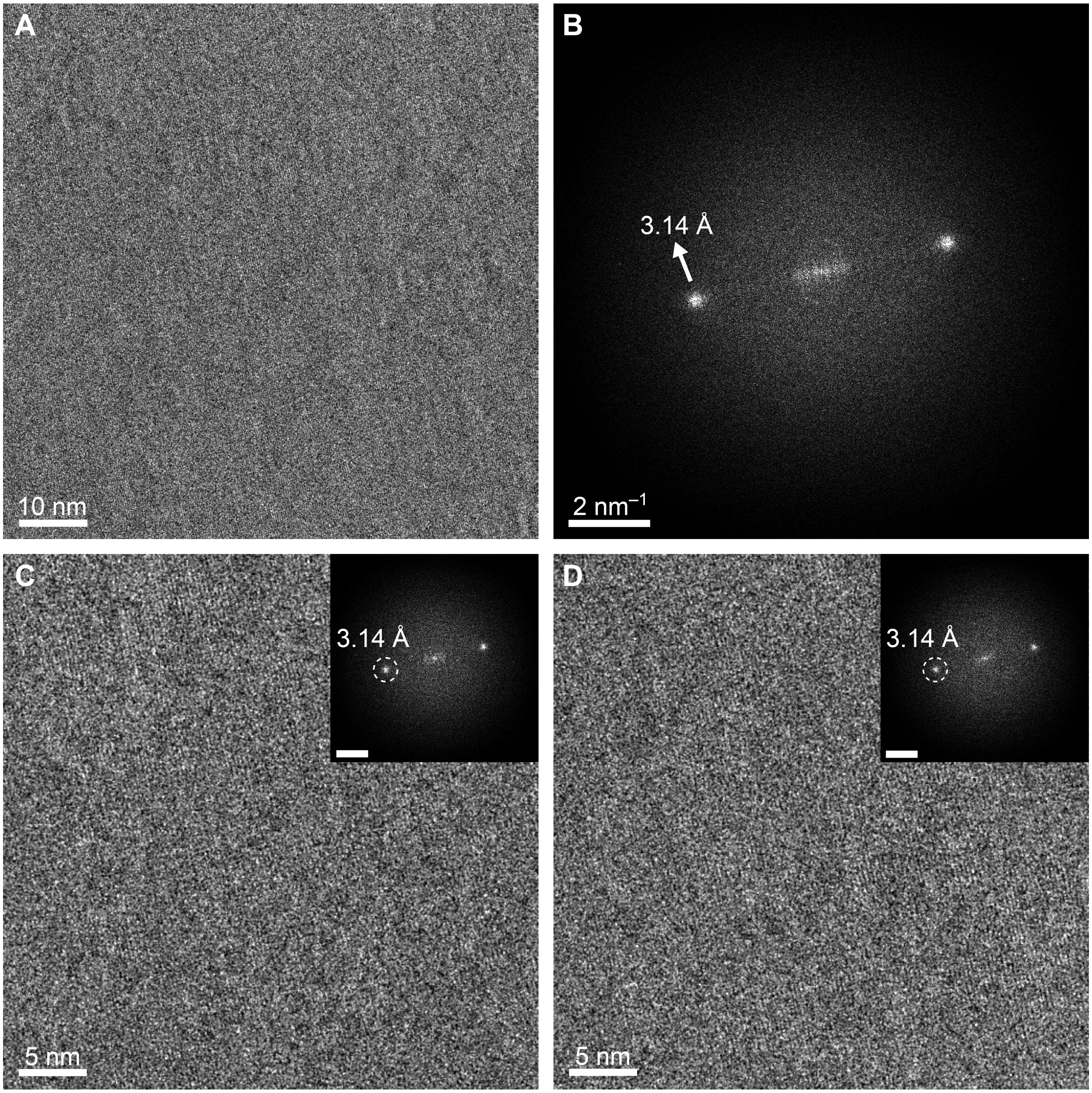

Figure S5. High magnification Cryo-EM images of TAQ.

(A, B) The yellow dashed square area in Figure S4A and its corresponding FFT. **(C)** The green dashed square area in Figure S4A and its corresponding FFT. **(D)** The blue dashed square area in Figure S4A and its corresponding FFT. Scale bars of FFT, 2 nm^{-1}. The π–π stacking of TAQ 2D layers can be directly seen and confirmed by FFT.

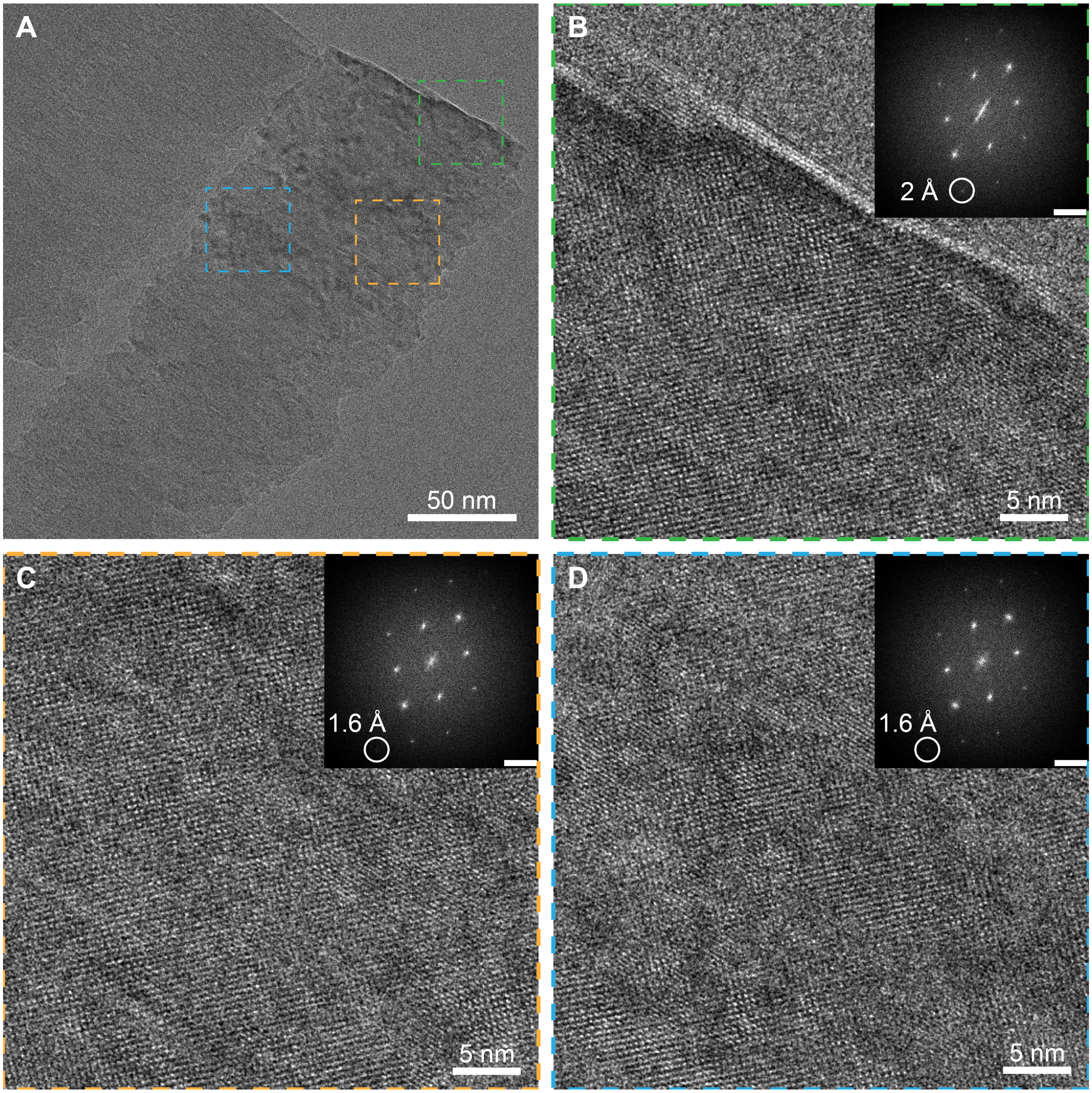

Figure S6. Cryo-EM images of TAQ.

(A) A Cryo-EM image of a single crystal of TAQ. **(B)** A high magnification micrograph of the green dashed square area and its FFT. **(C)** A high magnification micrograph of the yellow dashed square area and its FFT. **(D)** A high magnification micrograph of the blue dashed square area and its FFT. The short white dashes in **B**, **C**, and **D** are individual TAQ molecules. The highly ordered intermolecular solid-state packing is revealed by the FFT. The highest resolution is 1.6 Å. Scale bars of FFT, 2 nm^{-1}.

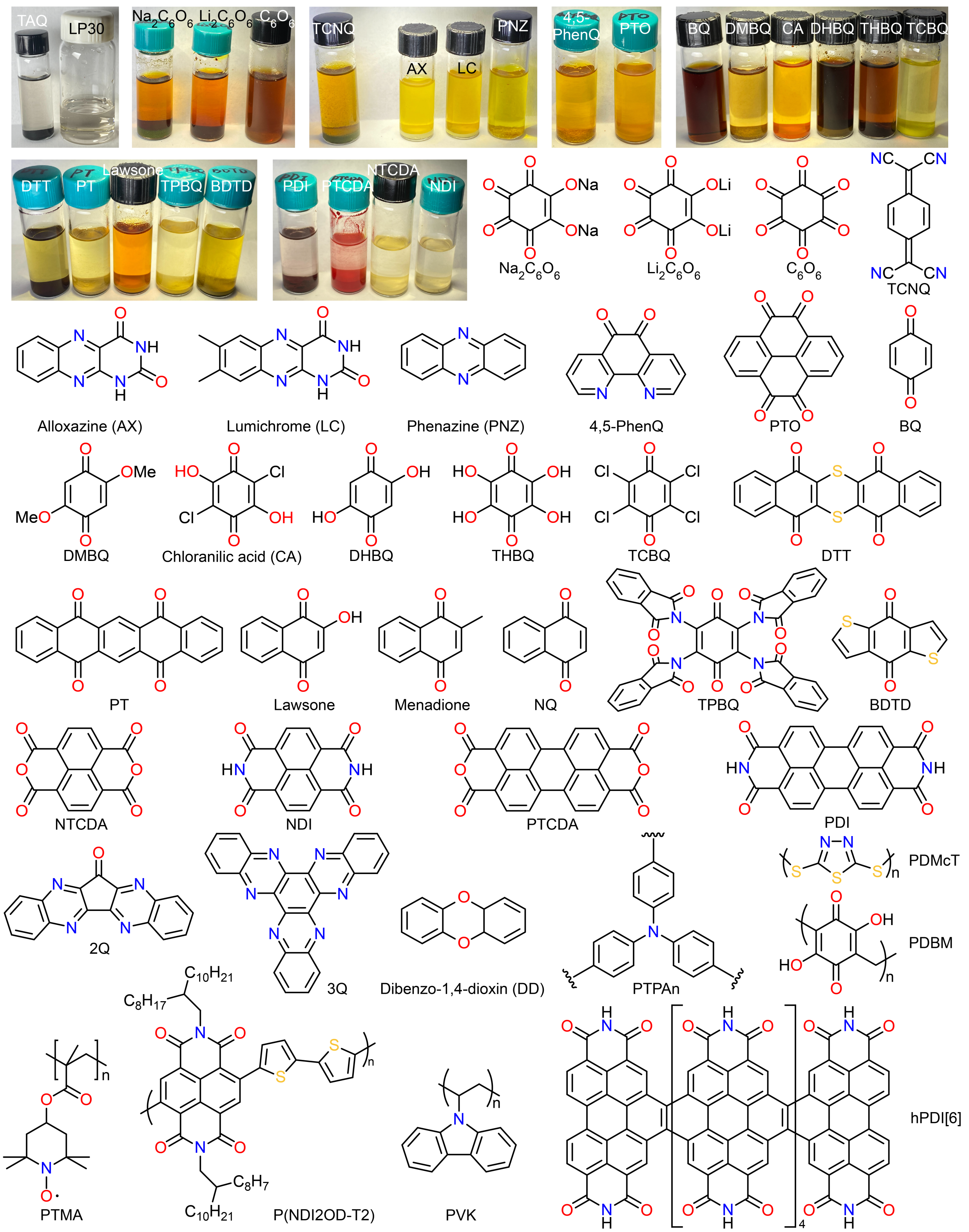

Figure S7. Solubility tests of common OEMs in LP30 electrolyte.

The photos were taken after soaking solids to sit in LP30 for three days. Whereas TAQ shows no dissolution, most OEMs undergo various degrees of dissolution in LP30.

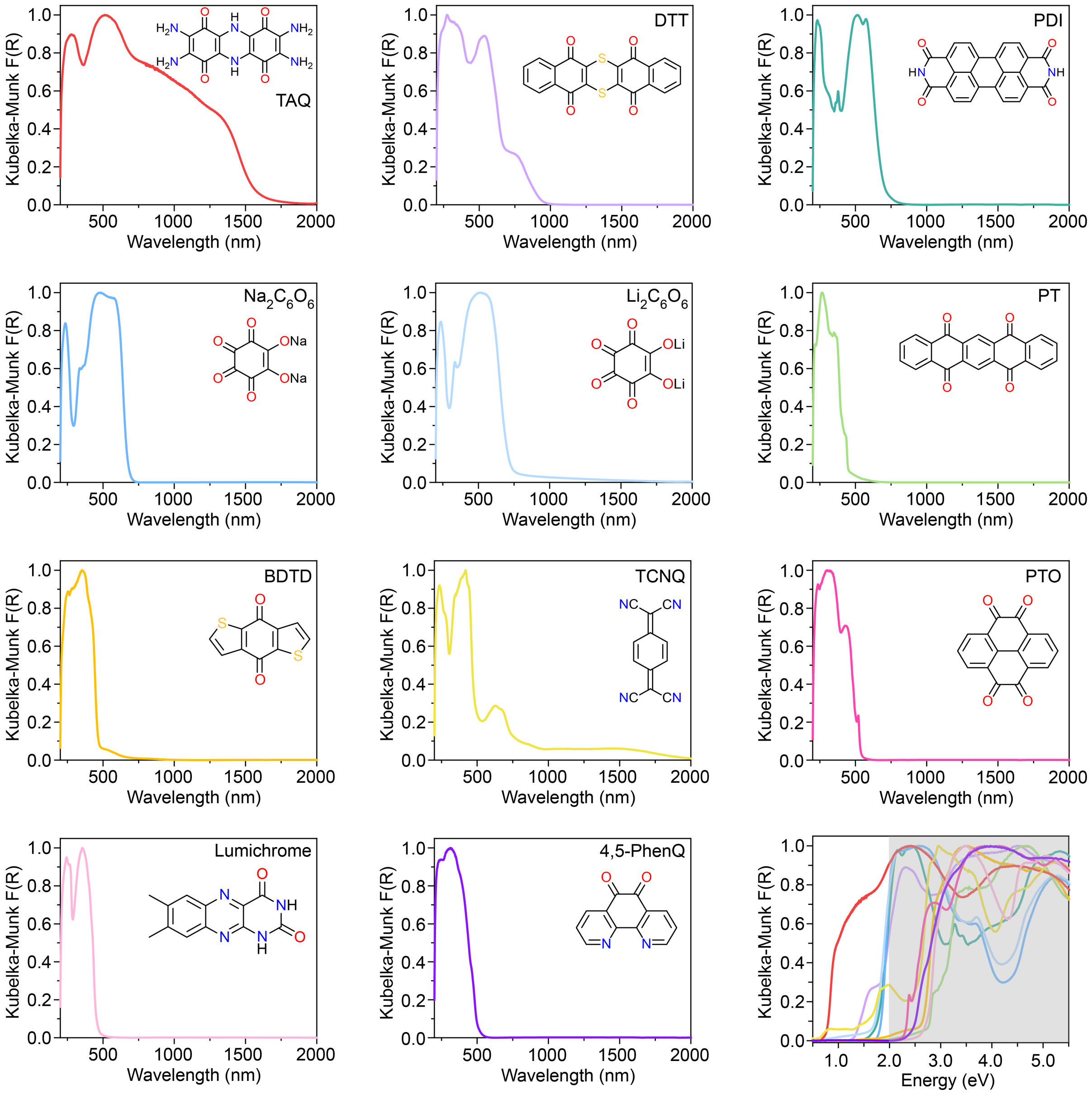

Figure S8. DRUV-Vis-NIR spectra of TAQ and selected OEMs.

DRUV-Vis spectra plotted in the energy scale (lower right corner) highlight that only TAQ exhibits significant low-energy absorption (< 2 eV).

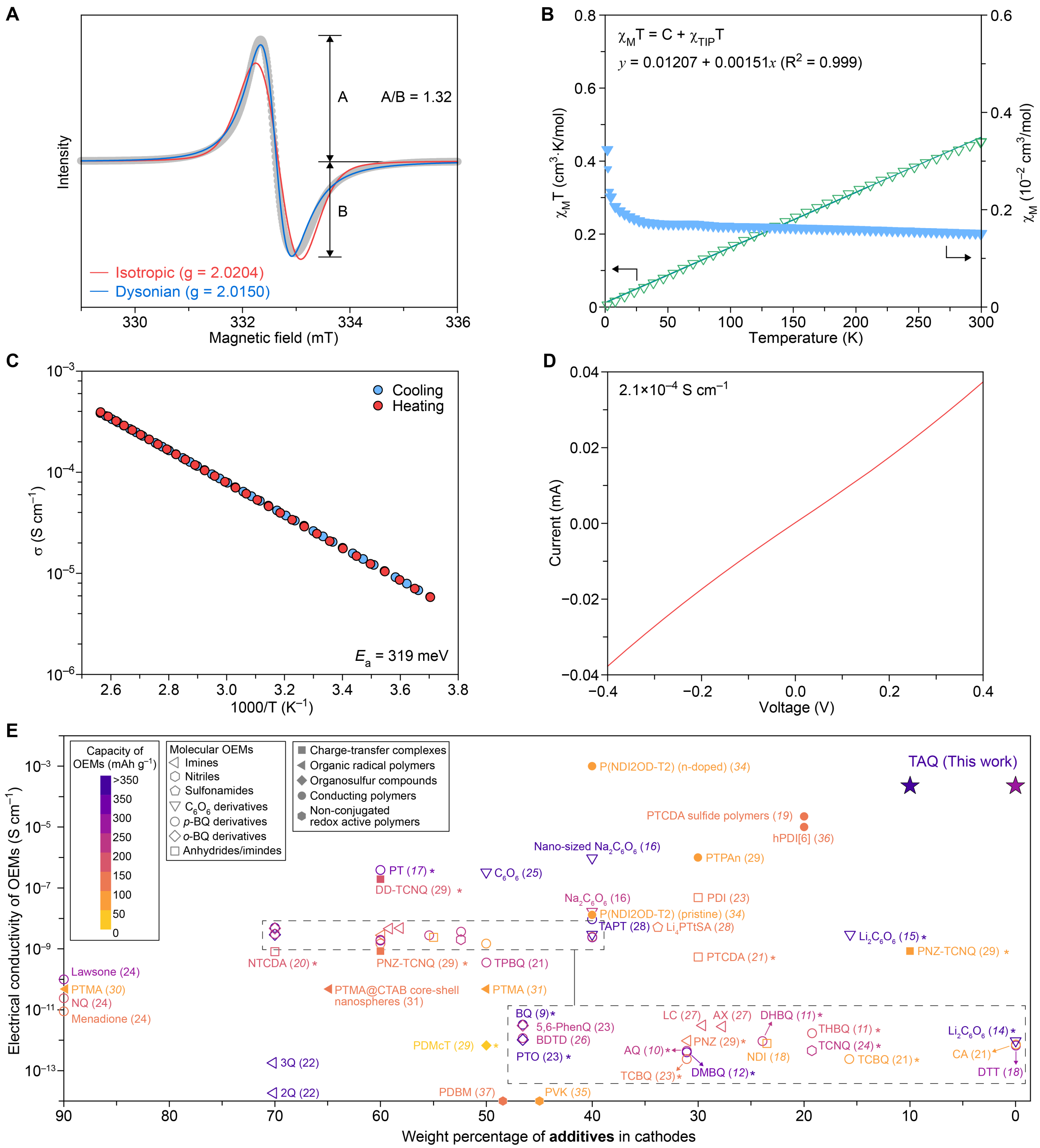

Figure S9. Electronic properties of TAQ and prototypical OEMs.

(A) EPR spectrum of TAQ at room temperature. The EPR signal is fitted better with a modified Dysonian lineshape with a g-factor of 2.0150 (blue trace) than with a Lorentzian lineshape with isotropic conventional broadening and a g-factor of 2.0204. The A/B ratio, which represents the EPR lineshape asymmetry and is indicative of electron delocalization, is 1.32. For localized electrons, the A/B ratio is strictly 1.[5] **(B)** Temperature dependence of magnetic susceptibility of TAQ measured at an applied field of 1000 Oe. The linear relationship of $\chi_M T$ versus T suggests the significant contribution of temperature-independent susceptibility (TIP) in addition to the Curie

component (i.e., the inset equation). The best linear fit gave a Curie constant of 0.01207 cm^3 mol^{-1} K, corresponding to a Curie spin density of 0.032 per TAQ molecule. TIP might be due to the presence of Pauli paramagnetism.[8] **(C)** Temperature dependence of the electrical conductivity of TAQ, revealing thermally-activated carrier transport behavior. Fitting of this data using the Arrhenius equation gives an activation energy E_a = 319 meV. **(D)** *I*-*V* curve of a TAQ pellet at room temperature gives an electrical conductivity of 2.1×10^{-4} S cm^{-1}. Typical values of electrical conductivity of TAQ range from 10^{-5} to 10^{-4} S cm^{-1}, with an average value of 7.7×10^{-5} S cm^{-1} based on six devices. **(E)** Experimentally measured electrical conductivity values of different classes of molecular OEMs and TAQ versus typical amounts of additives required for electrode fabrication. The raw data are presented in Table S3.[18–25, 41 in the main text; *9–37* in the SI] The reference numbers in *Italic* indicate references cited in the Supporting Information. The electrical conductivities of PDBM and PVK are essentially non-measurable. The electrodes based on lawsone, NQ, and menadione used advanced electrode architecture by infiltrating active materials into a porous gas-diffusion-layer (GDL) current collector, which leads to low active material content if considering the mass of the GDL. The electrical conductivity of P(NDI2OD-T2) increases upon discharge (n-doping) by ~5 orders of magnitude, and the electrical conductivity of PTPAn increases by ~6 orders of magnitude when fully charged.

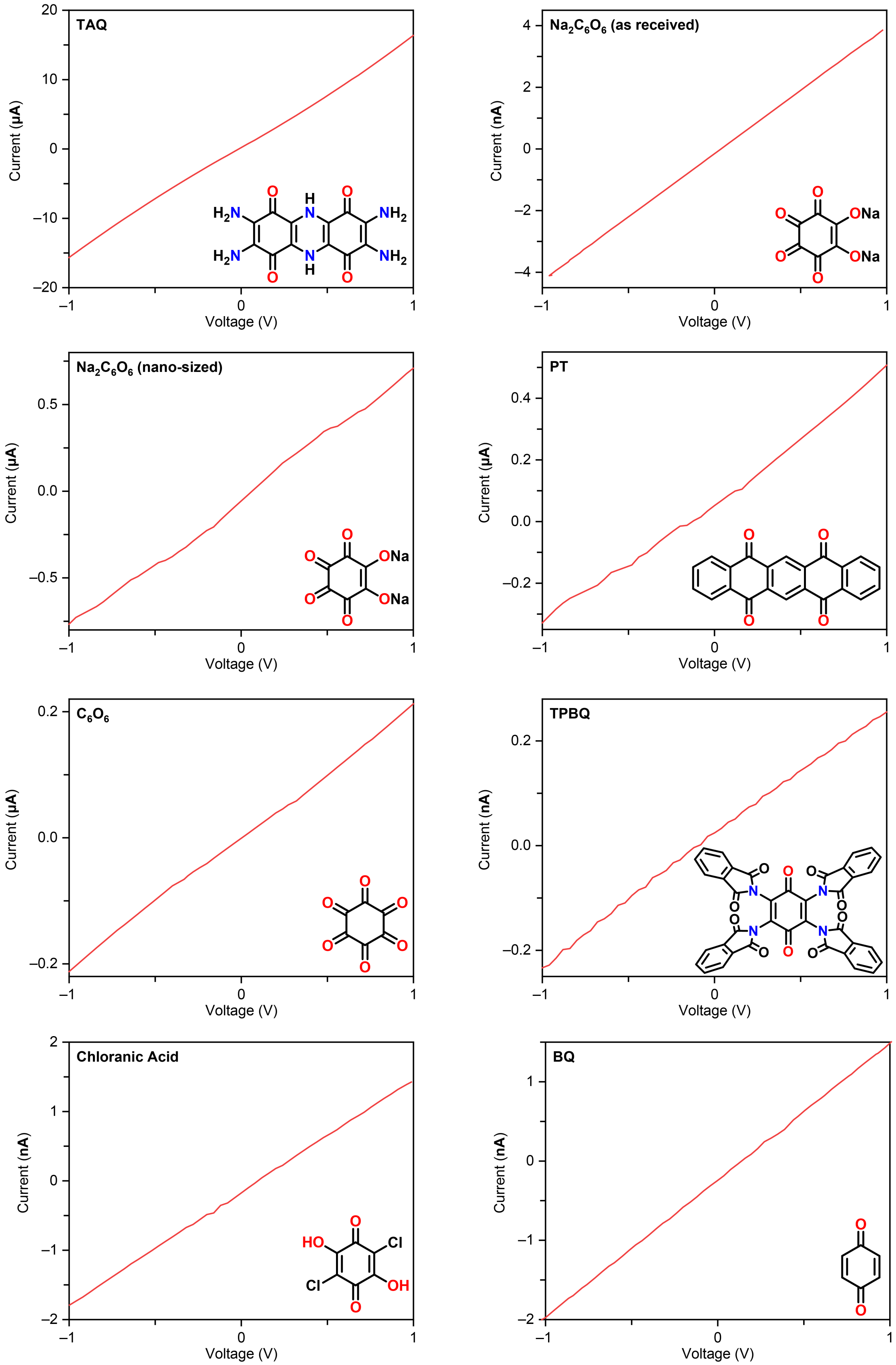
TAQ
Current (μA)
Voltage (V)
H2N
NH2
Na2C6O6 (as received)
Current (nA)
ONa
Na2C6O6 (nano-sized)
PT
C6O6
TPBQ
Chloranic Acid
HO
Cl
OH
BQ

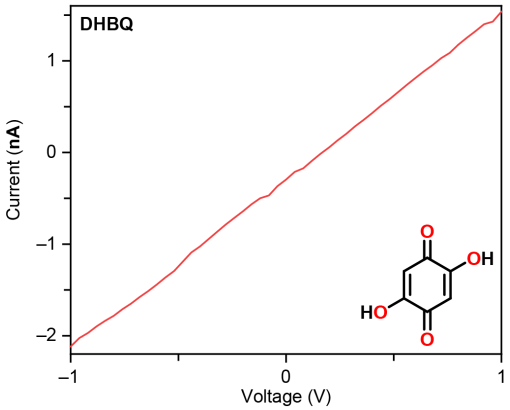
DHBQ
Current (nA)
1
0
−1
−2
−1
0
1
Voltage (V)
O
OH
HO
O

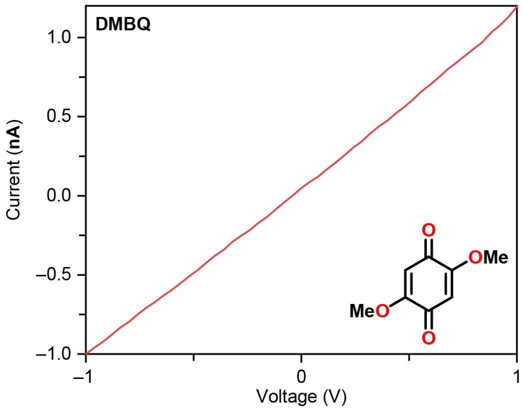
DMBQ
Current (nA)
1.0
0.5
0.0
−0.5
−1.0
−1
0
1
Voltage (V)
O
OMe
MeO
O

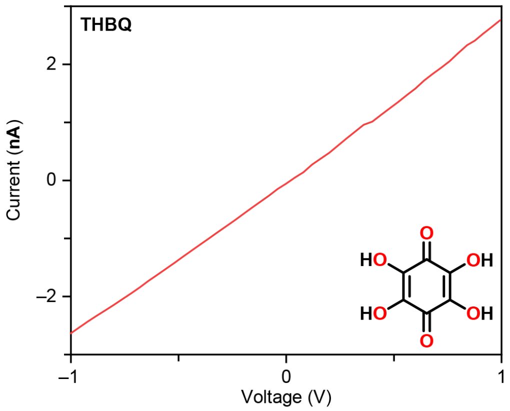
THBQ
Current (nA)
2
0
−2
−1
0
1
Voltage (V)
O
HO
OH
HO
OH
O

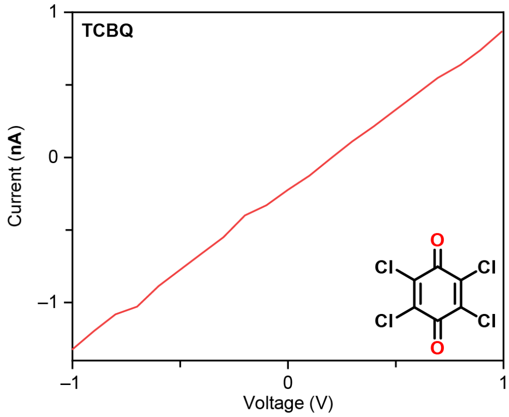
TCBQ
Current (nA)
1
0
−1
−1
0
1
Voltage (V)
O
Cl
Cl
Cl
Cl
O

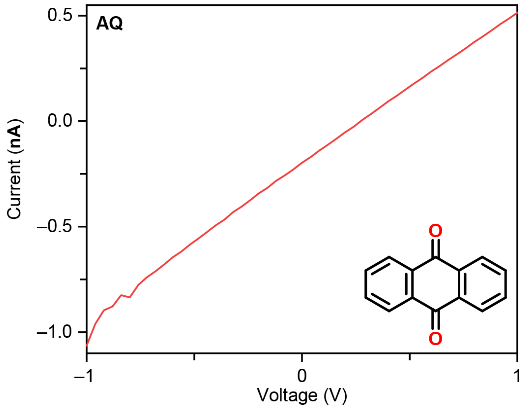
AQ
Current (nA)
0.5
0.0
−0.5
−1.0
−1
0
1
Voltage (V)
O
O

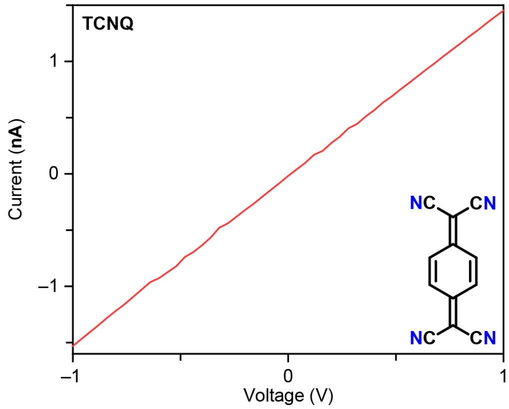
TCNQ
Current (nA)
1
0
−1
−1
0
1
Voltage (V)
NC
CN
NC
CN

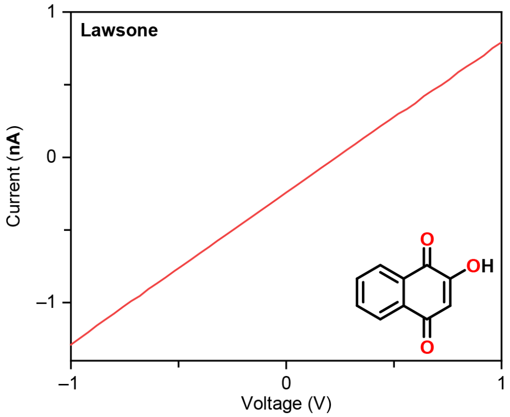
Lawsone
Current (nA)
1
0
−1
−1
0
1
Voltage (V)
O
OH
O

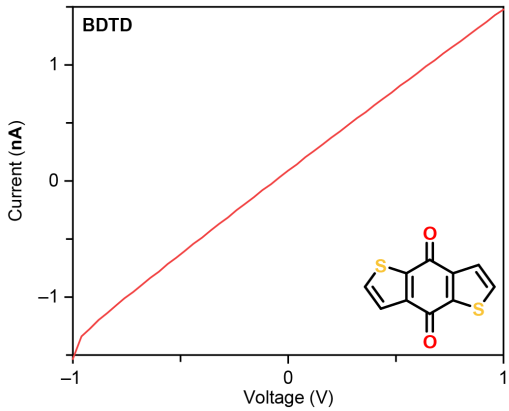
BDTD
Current (nA)
1
0
−1
−1
0
1
Voltage (V)
O
S
S
O

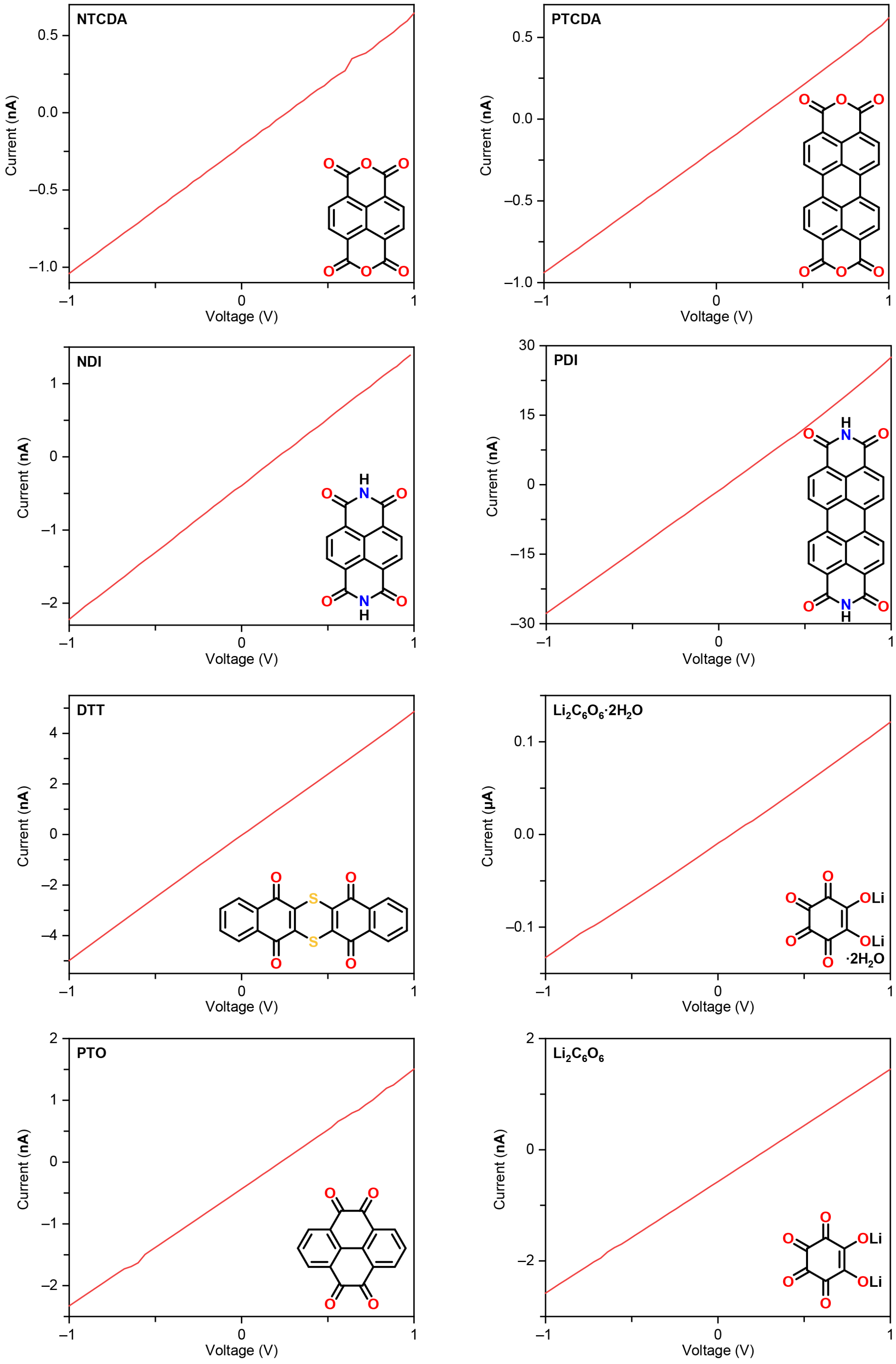
NTCDA
PTCDA
NDI
PDI
DTT
Li2C6O6·2H2O
PTO
Li2C6O6
Current (nA)
Current (μA)
Voltage (V)

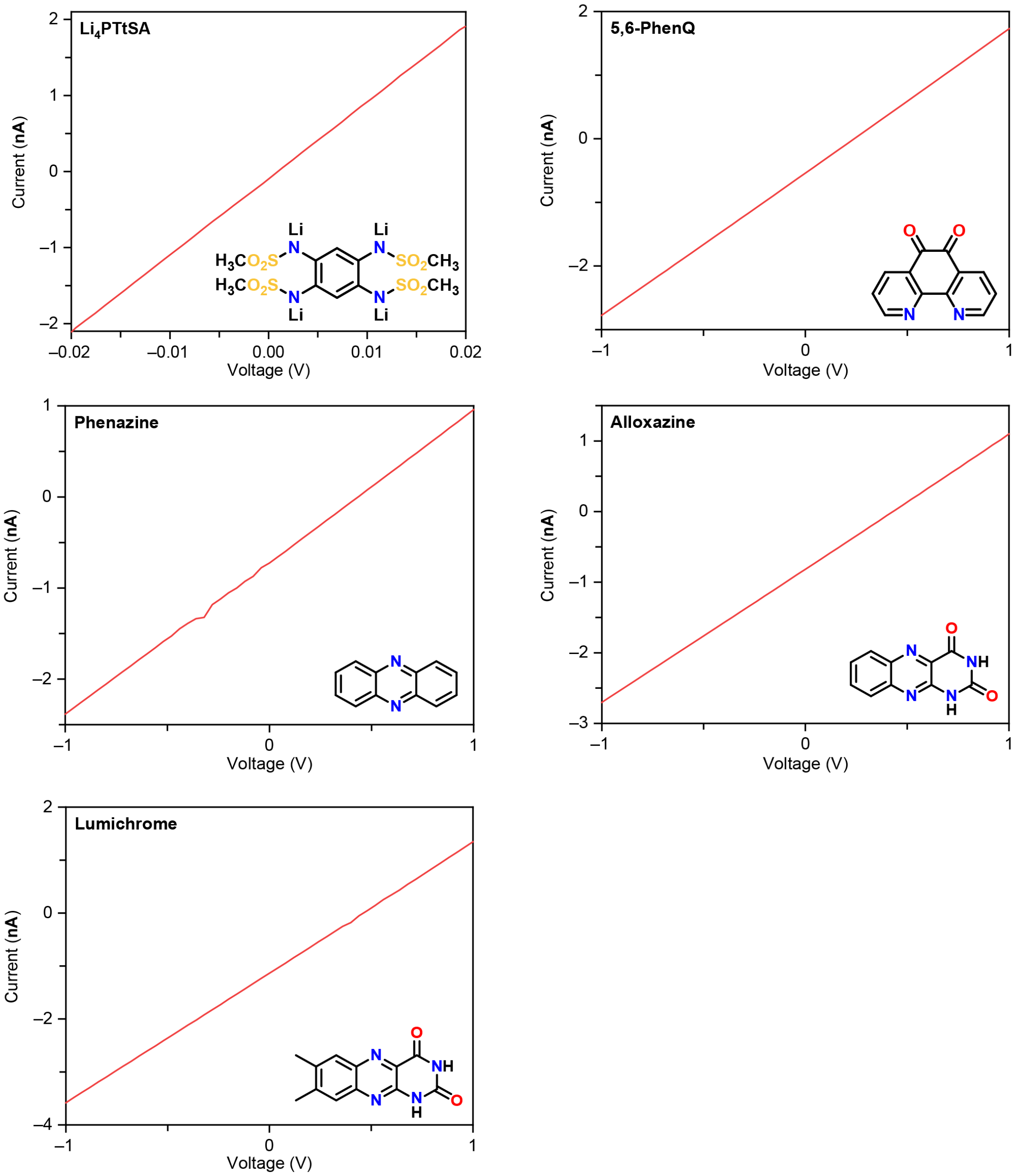

Figure S10.

IV curves of two-probe devices of TAQ and common OEMs with nominal voltages ≥ 2.0 V except for C_6O_6 (nominal voltage = 1.6 V).

Table S3.

Summary of electrical conductivity of common OEMs for LIB cathodes with nominal voltage ≥ 2.0 V, the active material content when they are used to make electrodes, and the corresponding specific capacity of active materials. The electrical conductivity values were measured using the two-probe pressed pellet configuration unless otherwise noted.

Chemical structure of OEMs	Active Material Content	Specific Capacity of **active material** (mAh g^{-1})	Electrical Conductivity of **active material** (S cm^{-1})	Reference
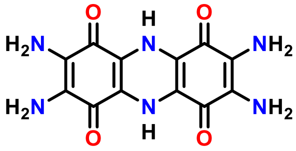 TAQ	**a) 100%**<br>**b) 90%**	**a) 297 (1st discharge, 25 mA g^{-1})**<br>**b) 356 (1st discharge, 25 mA g^{-1})**	**2.1×10^{-4}**	**This work**
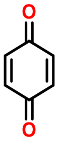 Benzoquinone (BQ)	30%	429 (1st discharge, 50 mA g^{-1})<br>137 (20th cycle, 50 mA g^{-1}) [a]	5.0×10^{-9}	*9*
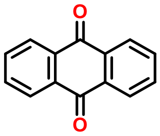 Anthraquinone (AQ)	40%	250 (1st discharge, 50 mA g^{-1})<br>100 (50th cycle, 50 mA g^{-1}) [a]	2.0×10^{-9}	*10*
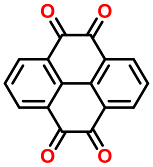 Pyrene-4,5,9,10-Tetraone (PTO)	30%	360 (0.05C) [a]	2.9×10^{-9}	23
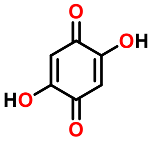 2,5-Dihydroxy-1,4-benzoquinone (DHBQ)	44.6%	~210 (0.2C) [a]	2.8×10^{-9}	*11*
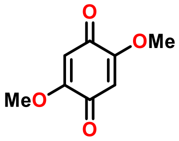 2,5-Dimethoxy-1,4-benzoquinone (DMBQ)	40%	312 (10 mA g^{-1}) [a]	1.9×10^{-9}	*12*

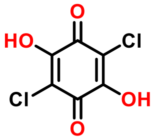 Chloranilic acid (CA)	60%	80 (50 mA g^{-1})	2.4×10^{-9}	20
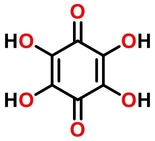 Tetrahydroxybenzoquinone (THBQ)	47.6%	~170 (0.2C) [a]	3.7×10^{-9}	*11*
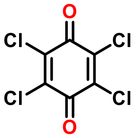 Tetrachlorobenzoquinone (TCBQ)	a) 50%<br>b) 40%	a) ~100 (0.1C) [a]<br>b) ~150 (10 mA g^{-1}) [a]	1.5×10^{-9}	a) 21<br>b) *13*
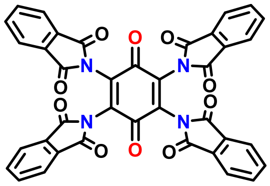 Tetraphthalimide benzoquinone (TPBQ)	50%	223.2 (0.2C)	3.6×10^{-10}	21
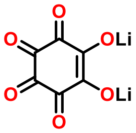 $Li_2C_6O_6$	(a) 60%<br>(b) 85%	a) ~580 (50 mA g^{-1}; 37% retention after 30 cycles) [a]<br>b) 580 (1st discharge, C/60); ~320 (10th cycle, C/60) [a]	3.0×10^{-9}	(a) *14*<br>(b) *15*
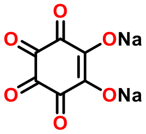 $Na_2C_6O_6$	60%	a) 210 (50 mA g^{-1})<br>b) 484 (50 mA g^{-1})	a) 1.7×10^{-8} (as received)<br>b) 9.3×10^{-7} (nano-sized)	*16*
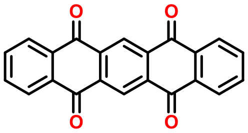 5,7,12,14-Pentacenetetrone (PT)	40%	304 (1st discharge, 20 mA g^{-1}); 170 (10th cycle, 20 mA g^{-1}) [a]	3.9×10^{-7}	*17*
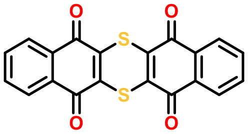	60%	261 (0.2C)	2.5×10^{-9}	*18*

Dibenzo[*b*,*i*]thianthrene-5,7,12,14-tetraone (DTT)				
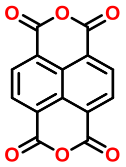 Naphthalenetetracarboxylic dianhydride (NTCDA)	a) 80%<br>b) 60%<br>c) 30%	a) 216 (0.1 A g^{-1}) [b]<br>b) 610.5 (50 mA g^{-1}) [c]<br>c) 191 (0.05C) [a]	8.1×10^{-10}	a) *19*<br>b) *20*<br>c) 23
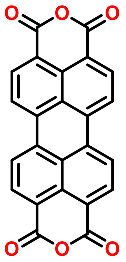 Perylenetetracarboxylic dianhydride (PTCDA)	70%	134 (10 mA g^{-1}) [a]	5.4×10^{-10}	*21*
PTCDA sulfide polymers	80%	148 (0.1 A g^{-1})	3.1×10^{-5}	*19*
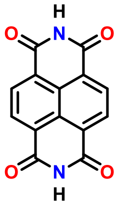 Naphthalenediimide (NDI)	45%	92 (0.2C)	2.4×10^{-9}	*22*
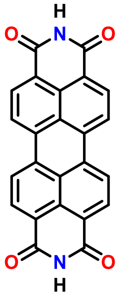 Perylenediimide (PDI)	70%	138 (C/6 or 20 mA g^{-1})	4.9×10^{-8}	*23*
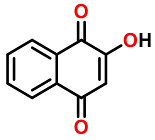 Lawsone	Loaded onto porous gas-diffusion layer (GDL) current collector	280 (0.2C or 54 mA g^{-1})	1.01×10^{-10} [d]<br>2.3×10^{-9}	24

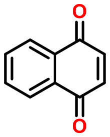<br>1,4-Naphthoquinone (NQ)	Loaded onto porous GDL current collector	190 (0.2C or 54 mA g^{-1})	2.36×10^{-11} [d]	24
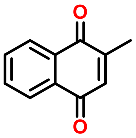<br>Menadione	Loaded onto porous GDL current collector	130 (0.2C or 54 mA g^{-1})	8.98×10^{-12} [d]	24
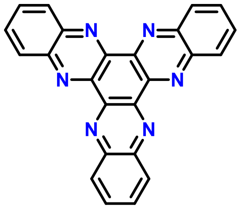<br>Triquinoxalinylene (3Q) [e]	30%	394 (1C or 0.4 A g^{-1})	1.78×10^{-13} [d]	22
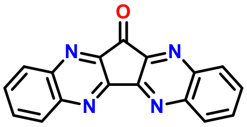<br>Diquinoxalinylene (2Q) [e]	30%	373 (1C or 0.4 A g^{-1})	2.13×10^{-14} [d]	22
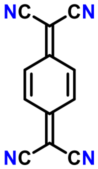<br>Tetracyanoquinodimethane (TCNQ)	47.6%	~220 (1st discharge) and ~30 (5th cycle) at 0.2C [a]	2.0×10^{-9}	*24*
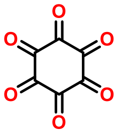<br>Hexaketocyclohexane (C_6O_6) [f]	50%	902 (20 mA g^{-1})<br>656 (50 mA g^{-1})	3.3×10^{-7}	*25*
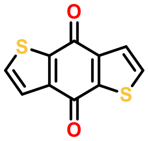<br>Benzo[1,2-*b*:4,5-*b′*]dithiophene-4,8-dione (BDTD)	30%	208 (0.1C)	3.1×10^{-9}	*26*

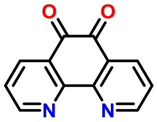 1,10-Phenanthroline-5,6-dione (5,6-PhenQ)	30%	231 (0.05C)	4.7×10^{-9}	23
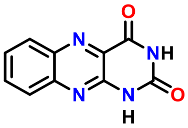 Alloxazine (AX)	41%	181 (20 mA g^{-1})	4.6×10^{-9}	*27*
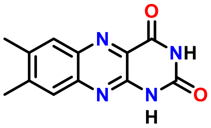 Lumichrome (LC)	43%	169 (20 mA g^{-1})	4.8×10^{-9}	*27*
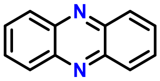 Phenazine (PNZ)	40%	134 (50 mA g^{-1}) [a]	2.8×10^{-9}	29
PNZ-TCNQ charge-transfer complex	a) 40%<br>b) 90%	a) 134 (50 mA g^{-1}) [a]<br>b) ~90 (50 mA g^{-1}) [a]	8×10^{-10} [d]	29
Dibenzo-1,4-dioxin (DD)-TCNQ charge-transfer complex	40%	171 (50 mA g^{-1}) [a]	2.785×10^{-7} [d]	29
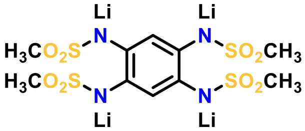 Li_4PTtSA	67%	~110 (0.1C)	5.1×10^{-9}	*28*
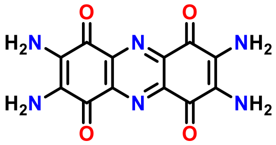 TAPT	60%	354 (0.1 A g^{-1})	1.16×10^{-8} [d]	28
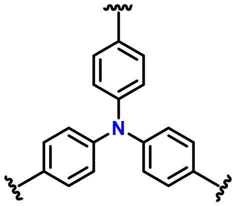 Polytriphenylamine (PTPAn)	70%	~100 (50 mA g^{-1})	~1×10^{-6} (pristine) [d]<br><br>~1 (doped during charge) [d]	29
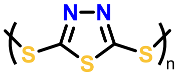	50%	30 (0.1 mA cm^{-2} or 30.6 mA g^{-1}) after three cycles [a]	5.9×10^{-13} [d]	*29*

poly(2,5-dimercapto-1,3,4-thiadiazole) (PDMcT)				
Conductive polypyrrole (PPy)-coated PDMcT	50%	150 (0.1 mA cm^{-2} or 30.6 mA g^{-1}) after three cycles	2.91×10^{-3} [d]	*29*
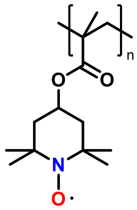 poly (2,2,6,6-tetramethylpiperidinyl oxy methacrylate) (PTMA)	a) 10%<br>b) 50%	a) 77 (0.1 mA cm^{-2})<br>b) ~80 (0.8C)	$\sim5\times10^{-11}$ [d]	a) *30*<br>b) *31*<br>c) *32*
PTMA/graphene	10%	222 (0.1C)	$\sim5\times10^{-11}$ [d]	*33*
PTMA@CTAB core-shell nanospheres	42.1% (based on nanospheres) or 35.07% (based on PTMA)	140.5 (0.2C)	$\sim5\times10^{-11}$ [d]	31
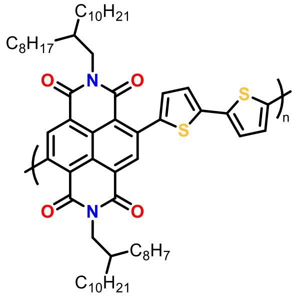 P(NDI2OD-T2)	60%	54.2 (1C)	$\sim10^{-8}$ (pristine) and $\sim10^{-3}$ (n-doped during dishcharge) [d]	*34*
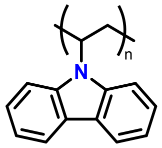 Poly(vinyl carbazole) (PVK) (composite with PEDOT:PSS)	55%	99.4 (1C)	1.2×10^{-17} (bare PVK) [d]<br>250 (PVK/PEDOT:PSS/CB cathode) [d]	*35*
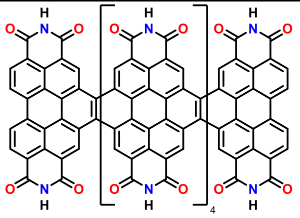 Helical perylene	80%	131 (0.1 A g^{-1} or 0.77C)	$\sim10^{-5}$ [d]	*36*

diimide (hPDI[6])				
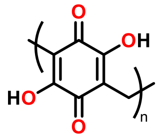 Poly(2,5-dihydroxy-1,4-benzoquinone-3,6-methylene) (PDBM) 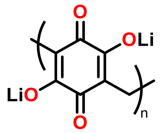 PDBM(Li)	52%	~150 (0.1 mA cm^{-2})	Not measurable [d]	*37*

[a] Rapid capacity decays. [b] The cell is not rechargeable after the second cycle due to electrode dissolution. [c] Used as an anode with a nominal voltage of ~0.7 V. Therefore this data point was not used in Figure 1i. [d] Reported value in the cited reference. [e] The nominal voltage is between 1.86 V and 1.88 V. [f] The nominal voltage is 1.6 V.

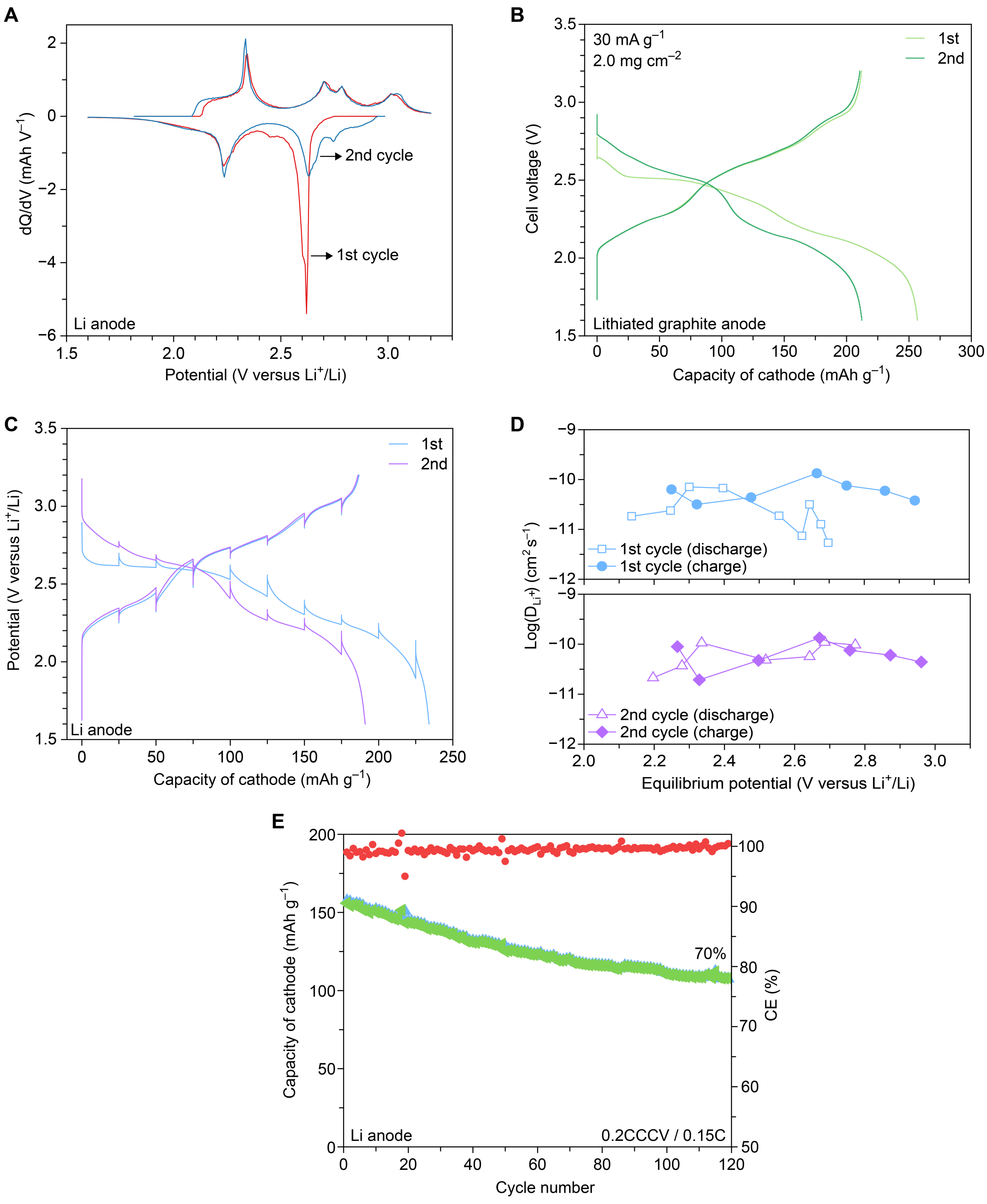

Figure S11. Battery performance of neat TAQ-based cells using LP30 electrolyte.
(A) dQ/dV curves of a TAQ||Li cell at 25 mA g^{-1}. **(B)** Typical GCD voltage profiles of neat TAQ||GrLi full cells recorded at a current density of 30 mA g^{-1}. **(C)** GITT voltage profiles of a neat TAQ||Li cell recorded at 0.1C. **(D)** Diffusion coefficients of Li^+ for the neat TAQ||Li cell calculated based on GITT data. **(E)** Slow cycling of a neat TAQ||Li cell at 0.2CCCV / 0.15C.

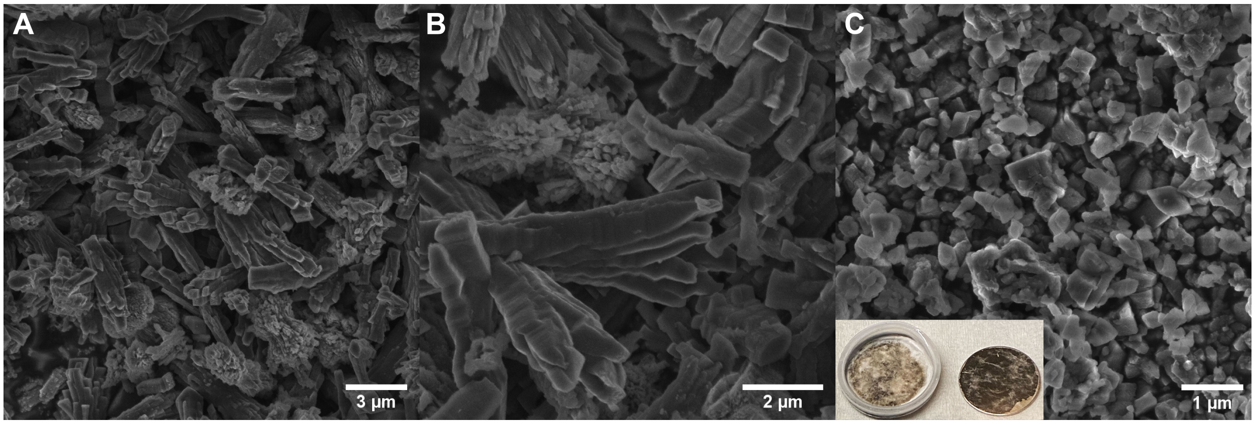

Figure S12. SEM images of pristine and cycled TAQ electrodes.

(A, B) Pristine TAQ electrodes. **(C)** TAQ electrode after 80 cycles at 0.2CCCV/0.1C. Inset shows the photo of the corresponding cycled electrode and separator, confirming little electrode dissolution has happened.

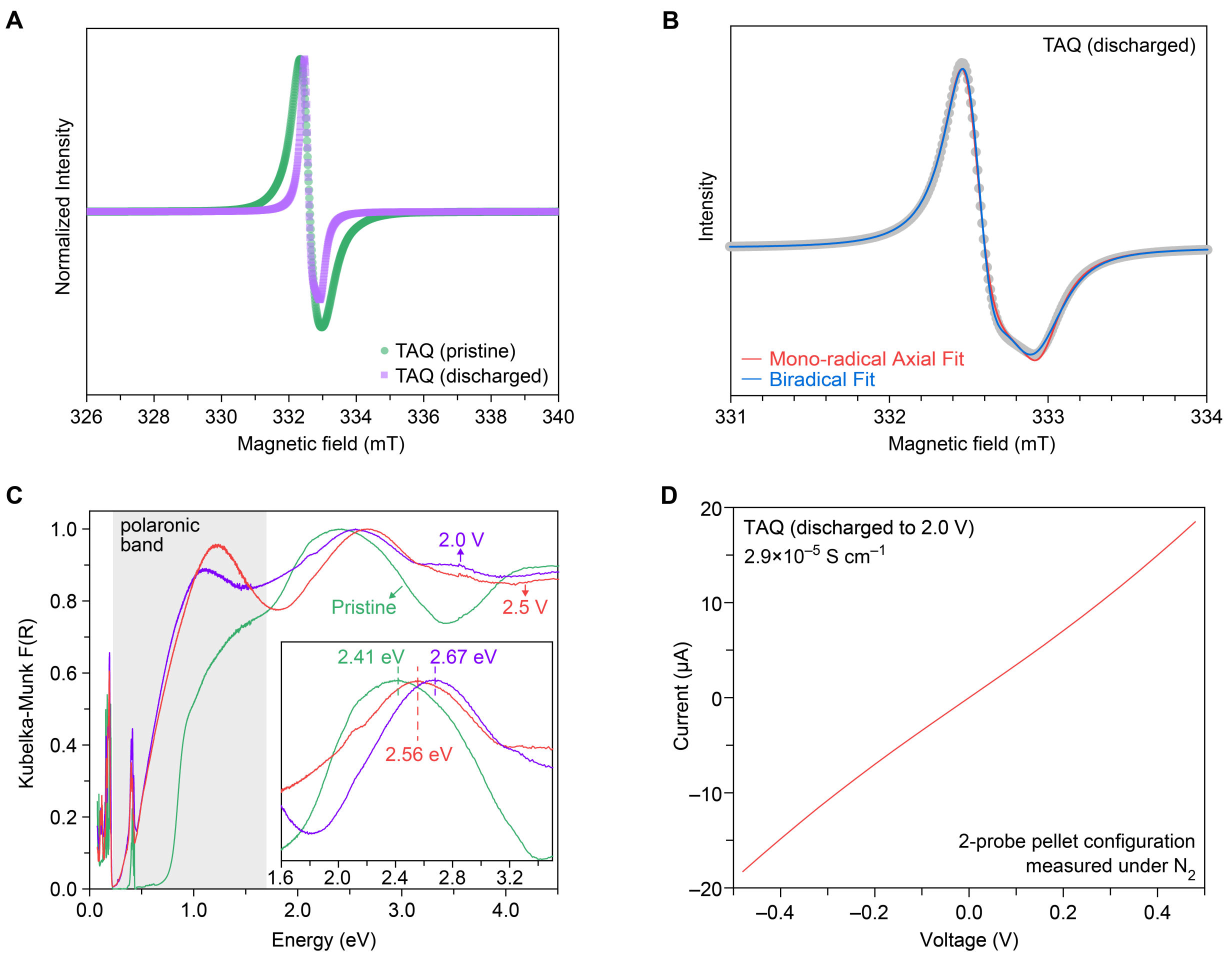

Figure S13. Studies of the charge storage mechanism of neat TAQ electrodes.

(A) Normalized EPR spectra of discharged TAQ (purple) and of pristine TAQ (green) at room temperature. The double integrated area under the EPR signals (non-normalized) gives a ratio of the spin molar density of discharged TAQ vs. pristine TAQ of ~26.3, corresponding to an estimated radical density in discharged TAQ of ~0.85 per molecule. **(B)** The EPR signal of discharged TAQ is fitted better with a biradical model with radicals on both O (g = 2.0203) and N atoms (g = 2.0191 with small hyperfine coupling) than with a mono-radical model with an axial g tensor ($g_{\perp}$ = 2.0206, $g_{\parallel}$ = 2.0180). **(C)** DRUV-Vis spectra of neat TAQ electrodes before discharging, discharged to 2.5 V, and to 2.0 V (versus Li^+/Li). Significant polaronic bands are evident around 1.0 eV. **(D)** *I-V* curve and corresponding electrical conductivity of TAQ discharged to 2.0 V. The obtained electrical conductivity value is on par with the average value of pristine TAQ.

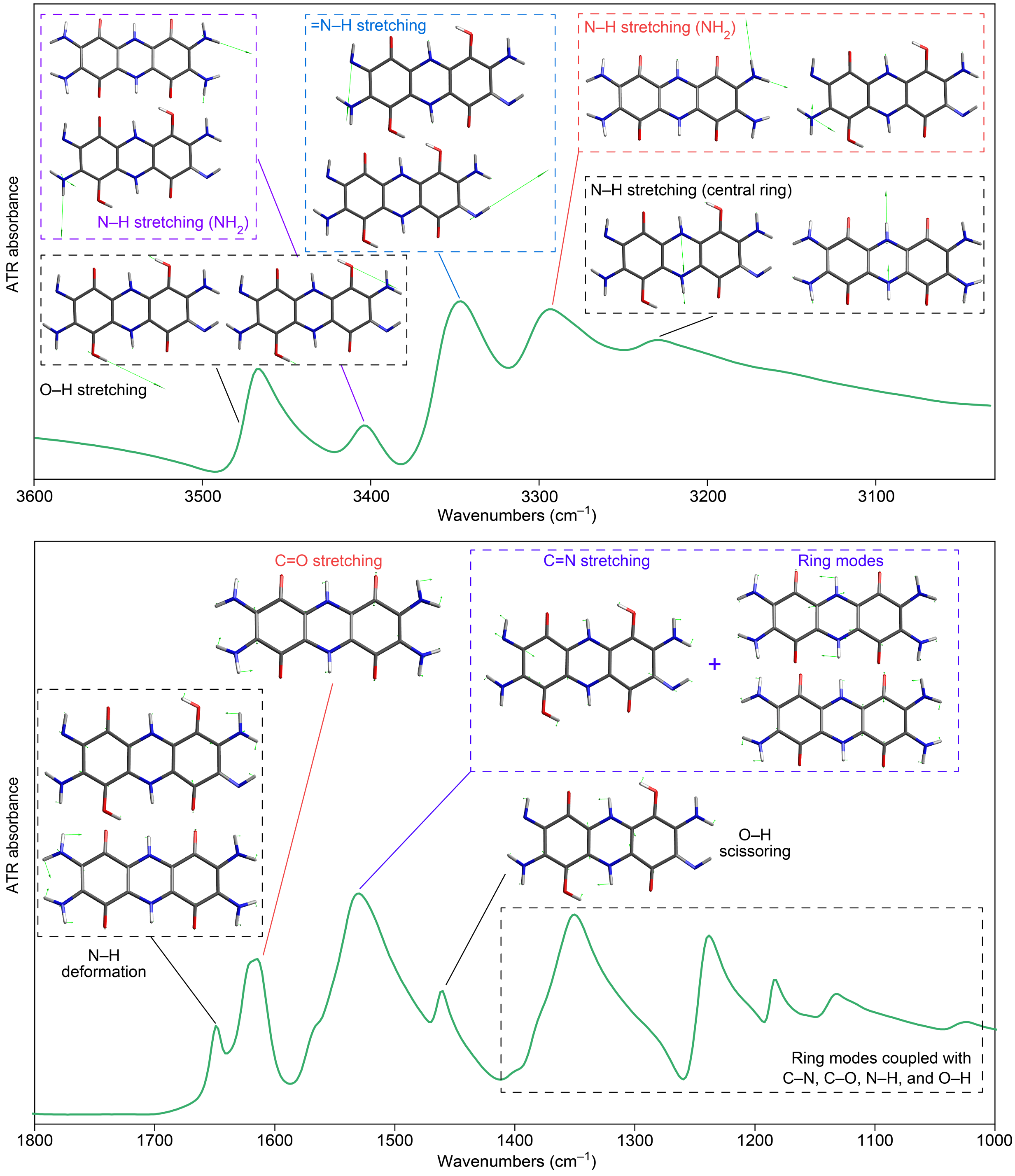

Figure S14.
ATR-FTIR spectrum of TAQ and the corresponding calculated vibrational modes.

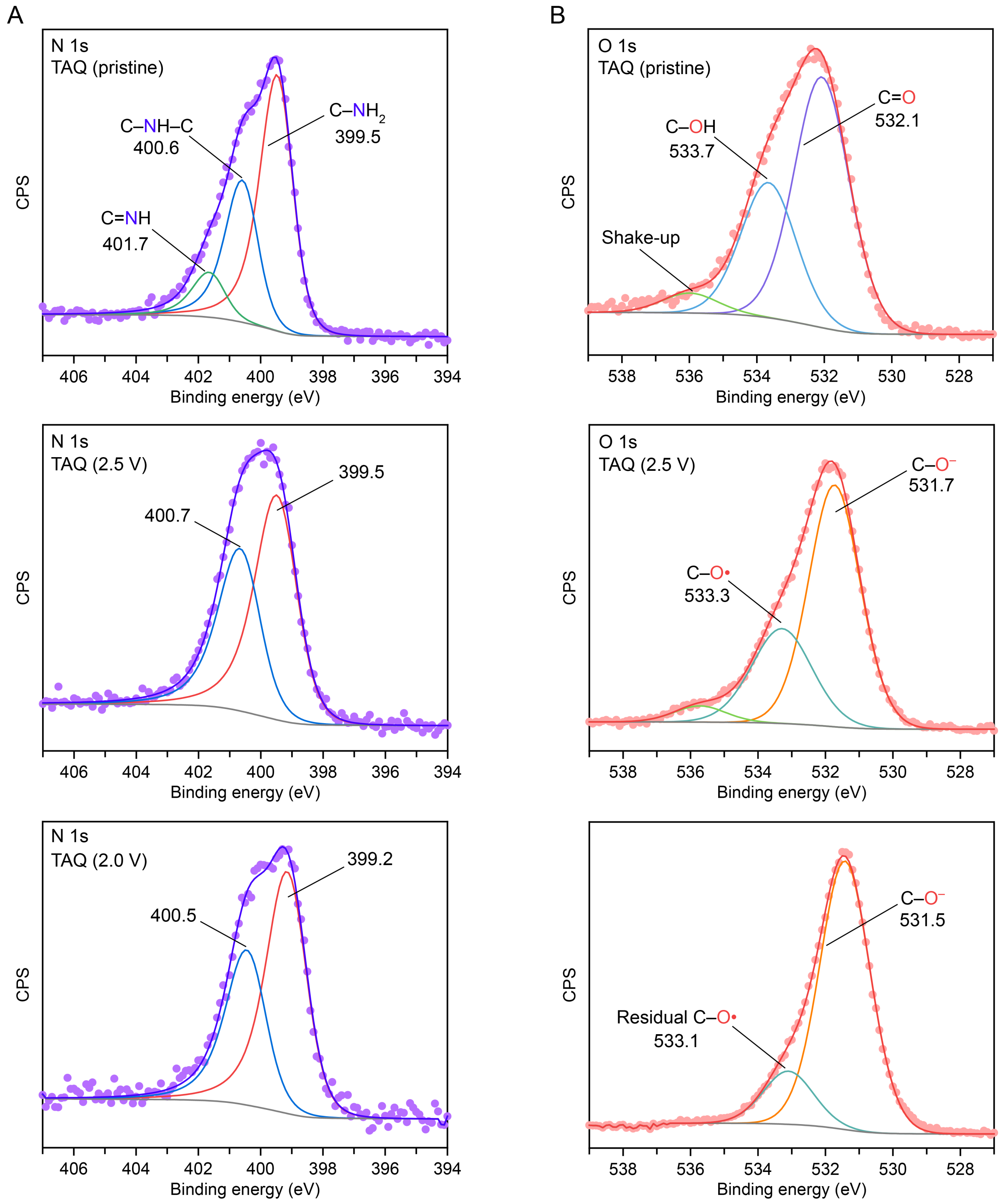

Figure S15.
High-resolution XPS analysis of pristine TAQ electrode, TAQ electrode discharged to 2.5 V, and TAQ electrode discharged to 2.0 V.

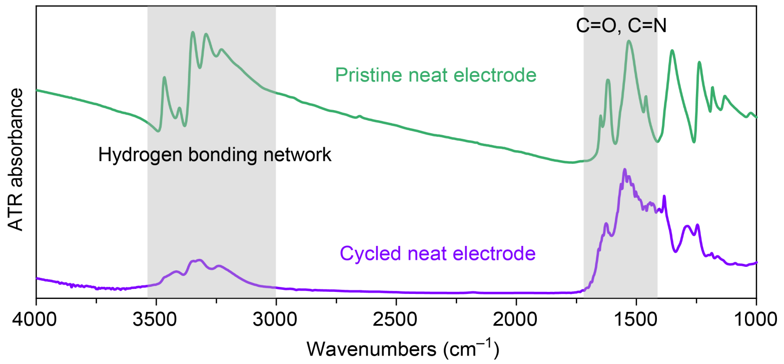

Figure S16.
ATR-FTIR spectra of neat TAQ electrodes in the pristine state and after slow cycling at 0.2CCCV/0.15C for 120 cycles.

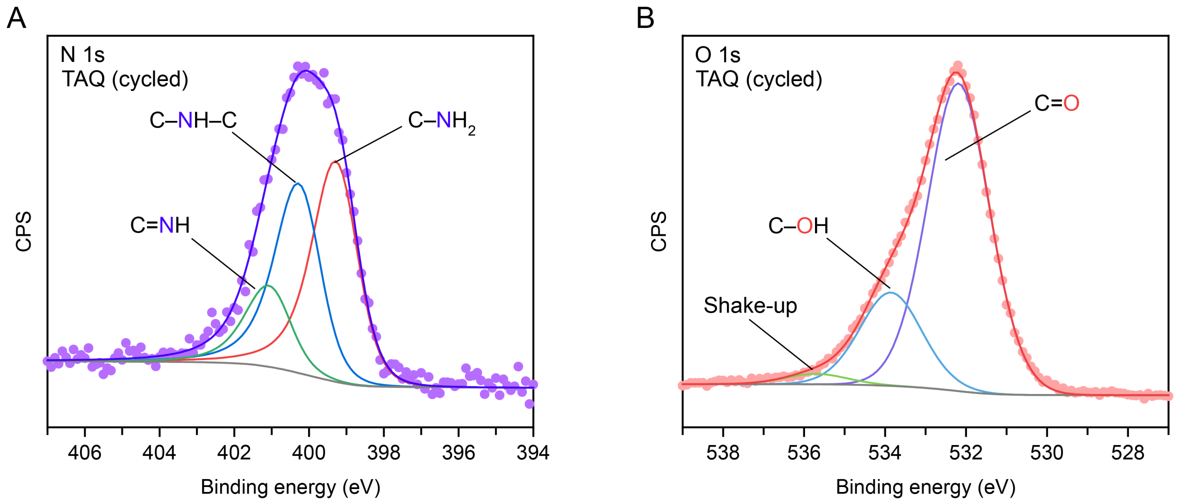

Figure S17.

XPS analysis of TAQ electrode after cycling. Little changes of both N 1s and O 1s spectra can be observed compared to the pristine state of TAQ.

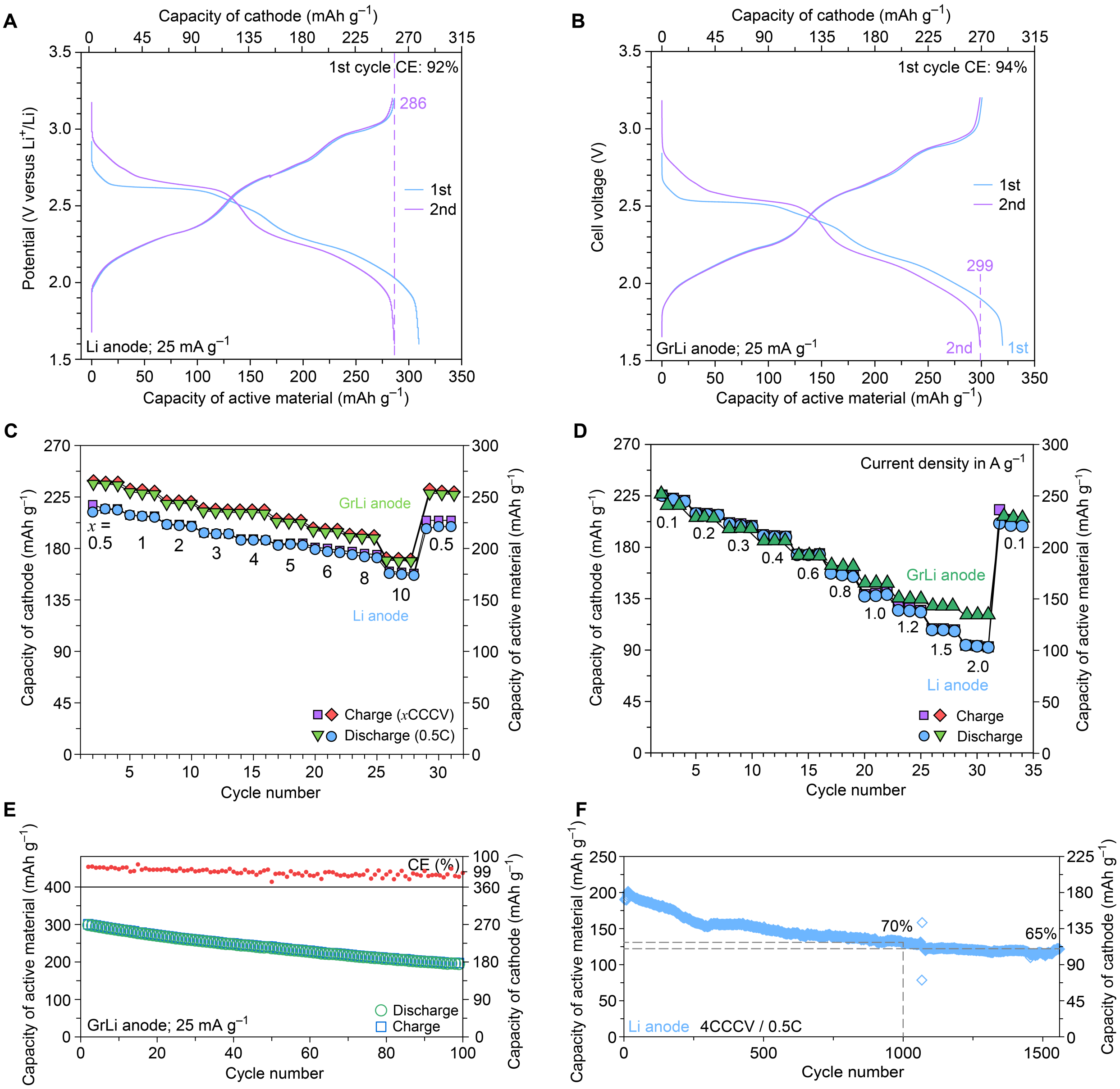

Figure S18. Battery performance of cells based on TAQ/CMC composite electrodes with 90% active content.

(A) The first- and second-cycle voltage profiles of a TAQ/CMC||Li cell recorded at 25 mA g^{-1} in LP30, exhibiting a first-cycle CE of 92%. **(B)** The first- and second-cycle voltage profiles of a TAQ/CMC||GrLi cell recorded at 25 mA g^{-1} in LP30, exhibiting a first-cycle CE of 94%. **(C)** Power capability studies of coin cells based on TAQ/CMC composite electrodes using Li and GrLi anodes. Constant current discharge rate of 0.5C and increasingly higher CCCV charging rates from 0.5 to 10CCCV were applied. **(D)** Power capability of a TAQ/CMC||Li cell and a TAQ/CMC||GrLi cell recorded from 0.1 A g^{-1} to 2 A g^{-1}. **(E)**. Constant-current cycling of a TAQ/CMC||GrLi full cell at 100% DOD and 25 mA g^{-1}. **(F)** Long-term 4CCCV/0.5C cycling of a TAQ/CMC||Li cell at 100% DOD, exhibiting 70% and 65% discharge capacity retention after 1000 and 1550 cycles, respectively.

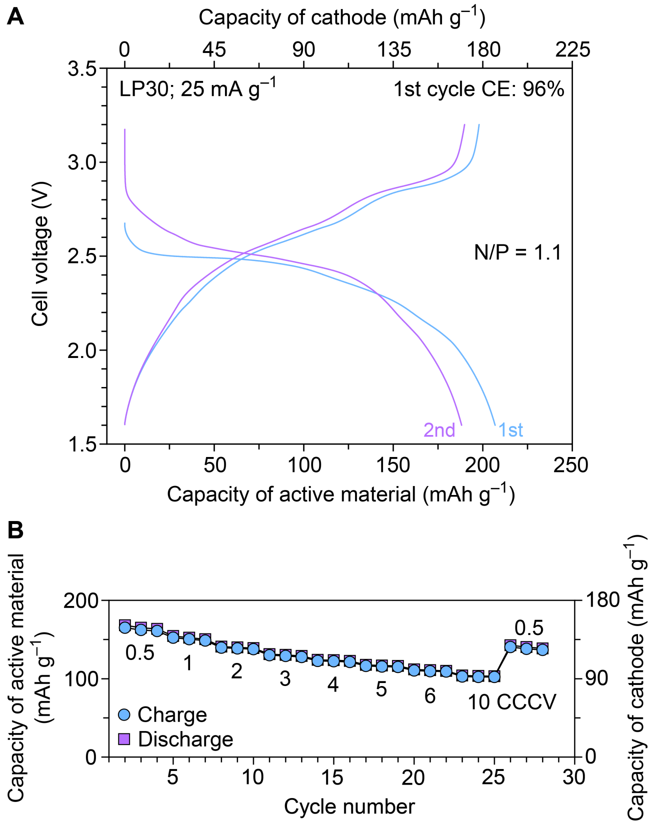

Figure S19. Electrochemical measurements of a TAQ/CMC||GrLi full cell with a N/P ratio of 1.1.

(A) The first- and second-cycle voltage profiles of the cell. **(B)** Power capability studies of the cell using various CCCV charging rates and a discharge rate of 0.5C.

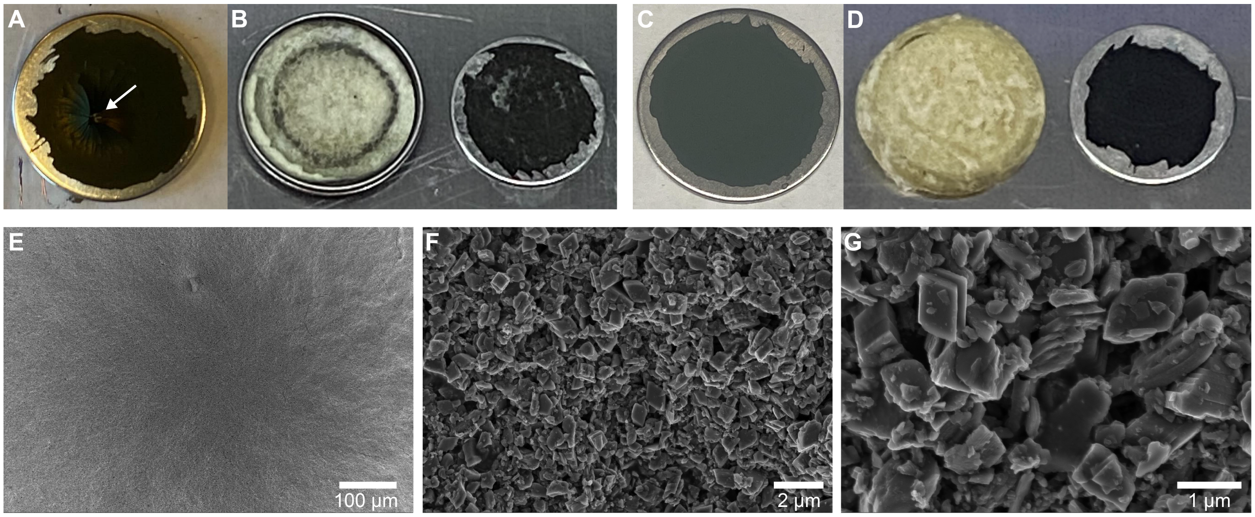

Figure S20. Characterization of TAQ composite electrodes using either only CMC or both CMC and SBR as the binder.

(A) A TAQ composite electrode using only CMC as the binder. **(B)** A TAQ composite electrode using only CMC as the binder and the separator after cycling. **(C)** A TAQ composite electrode using both CMC and SBR as the binder. **(D)** A TAQ composite electrode using both CMC and SBR as the binder and the separator after cycling. **(E-G)** SEM images of TAQ composite electrode using both CMC and SBR as the binder.

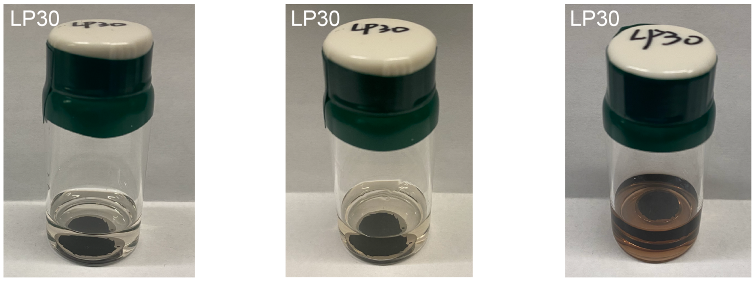

room temperature, 3 days | 80 °C, 24 hours | 100 °C, 24 hours

Figure S21. Soaking studies of TAQ/CMC/SBR composite electrodes in LP30.

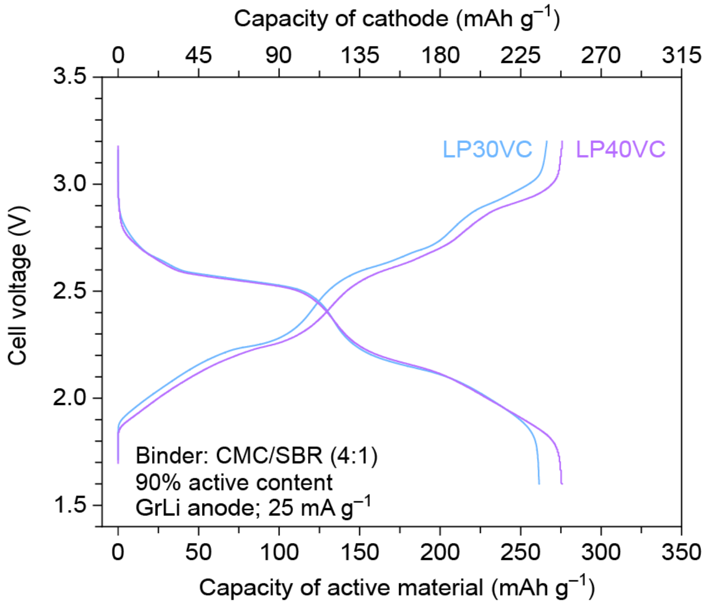

Figure S22.
Voltage profiles of TAQ/CMC/SBR||GrLi cells in LP30VC and LP40VC at 25 mA g^{-1}.

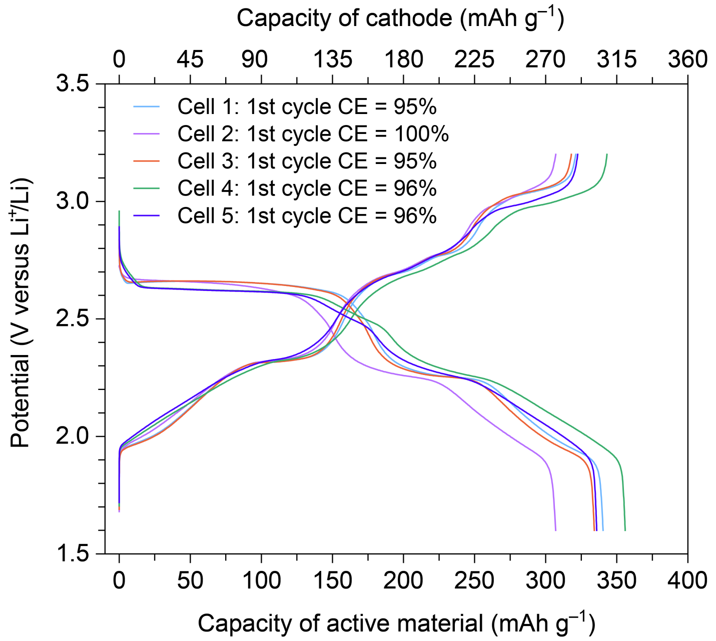

Figure S23.

Voltage profiles of five TAQ/CMC/SBR||Li cells in LiTFSI DOL/DME measured at 25 mA g^{-1}. The typical first-cycle Coulombic efficiency is no less than 95%.

Table S4

Summary of power rate capability data of TAQ/CMC/SBR||Li cells in LiTFSI/DOL/DME.

Current density (mA g^{-1})	Average charge/discharge capacity (mAh g^{-1}) of **active material** based on 600-hour cycling studies	Average charge/discharge capacity (mAh g^{-1}) of **cathode** based on 600-hour cycling studies
25	300.5/295.1	270.4/265.6
40	289.4/284.1	260.5/255.7
50	292.8/290.7	263.6/261.6
100	285.2/283.7	256.6/255.3
200	262.5/262.0	236.3/235.8
400	241.6/241.0	217.4/216.9
1000	203.2/202.6	182.9/182.4
2000	178.4/174.2	160.5/156.8

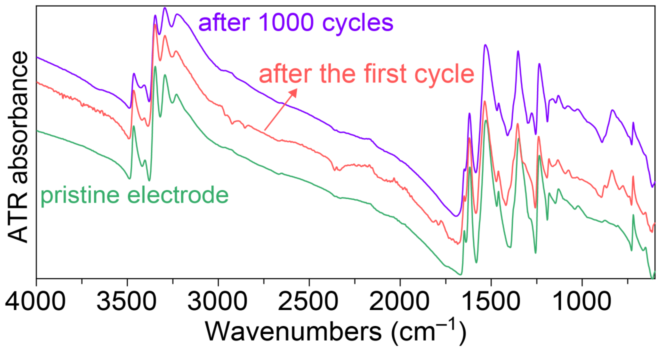

Figure S24. ATR-FTIR spectra of TAQ electrodes in the pristine state, after the first discharge-charge cycle, and after 1000 cycles at 2C.

The three essentially identical spectra reveal that the chemical and molecular structure of TAQ is resilient upon prolonged cycling.

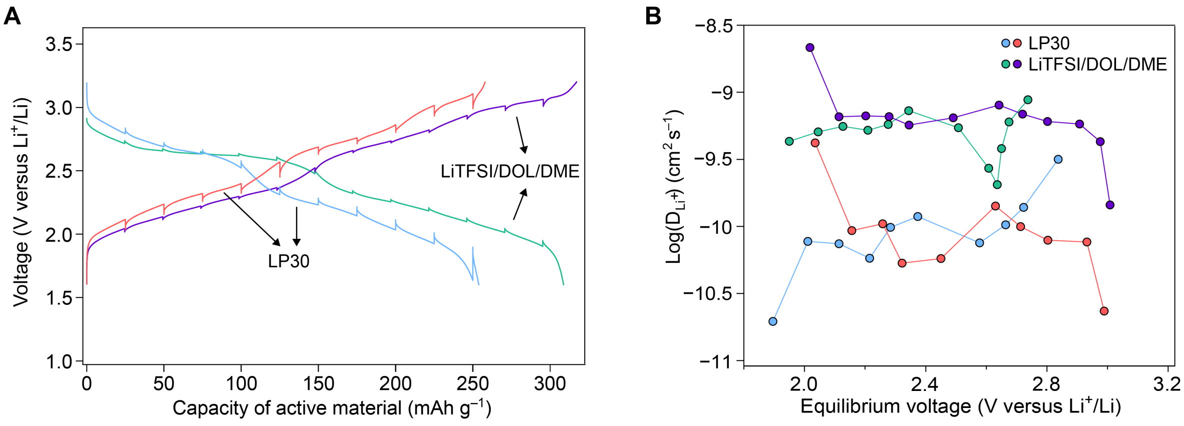

Figure S25. GITT analysis of TAQ/CMC/SBR||Li cells in different electrolytes.

(A) GITT voltage profiles of TAQ/CMC/SBR||Li cells in LP30 and LiTFSI/DOL/DME. **(B)** Li^+ diffusion coefficients obtained from GITT data in **(A)**.

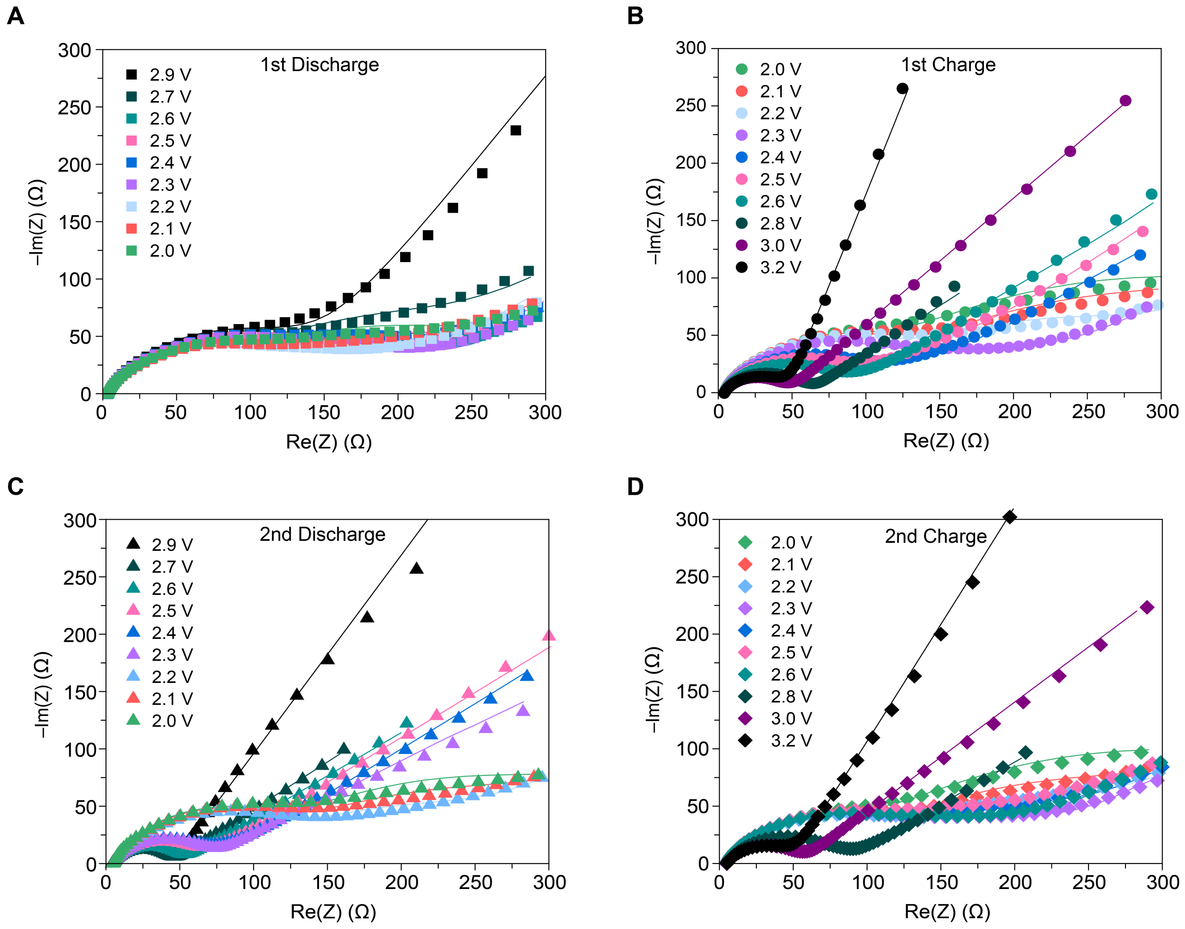

Figure S26. Nyquist plots of a TAQ/CMC/SBR||Li cell in LP30 measured at various discharge/charge states during the first and second cycles.

Solid lines represent the fitting of experimental data. Significant differences in the charge storage dynamics were observed during the first discharge process **(A)** relative to the first charge process **(B)** and the second cycle **(C, D)**.

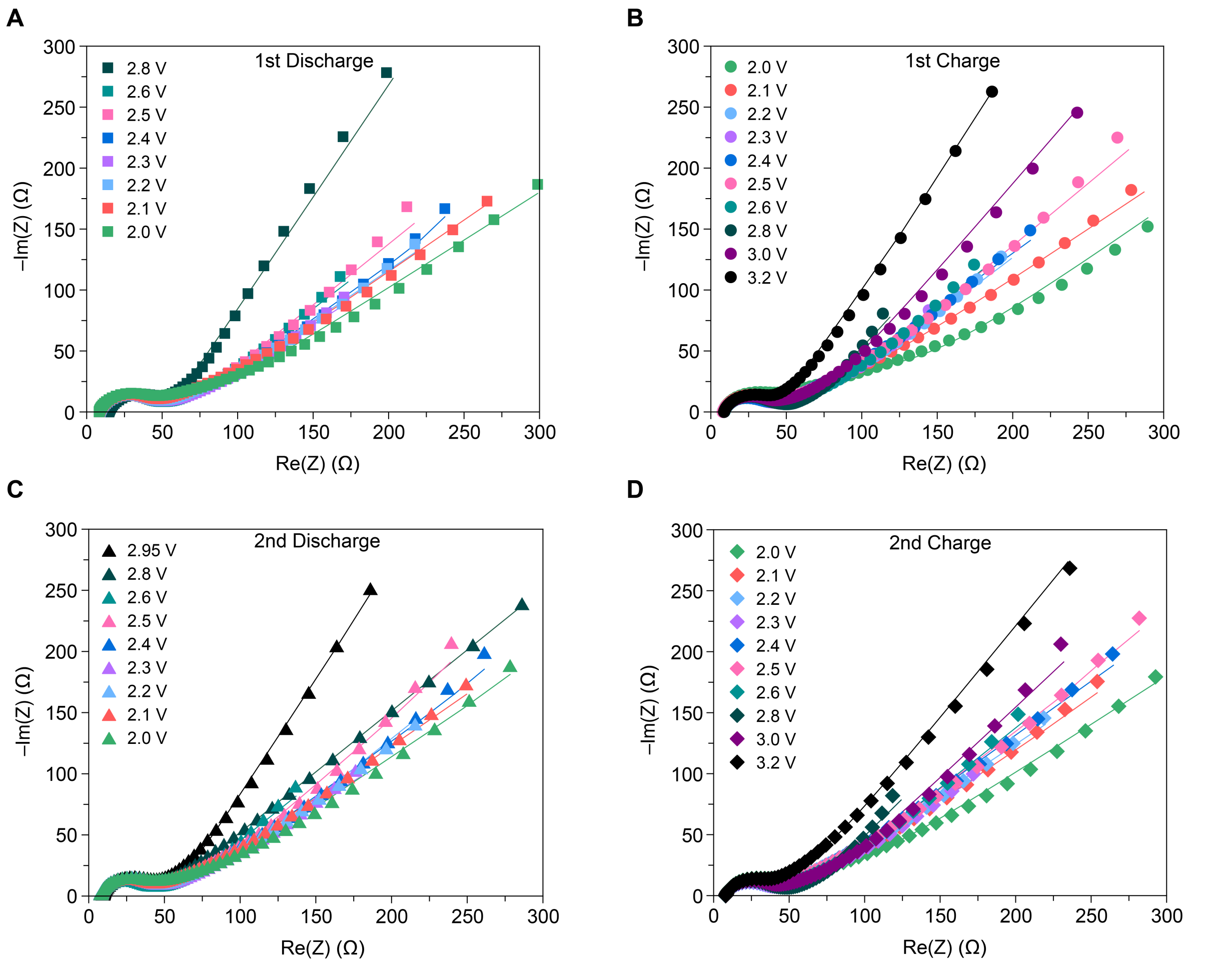

Figure S27. Nyquist plots of a TAQ/CMC/SBR||Li cell in LiTFSI DOL/DME measured at various discharge/charge states during the first and second cycles.

Solid lines represent the fitting of experimental data. Similar charge storage dynamics were observed during both cycles.

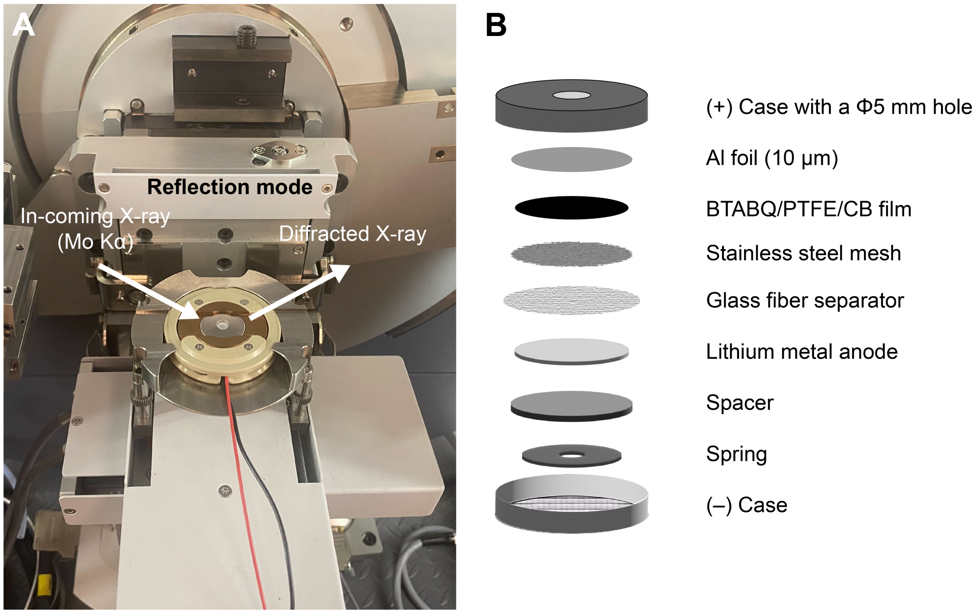

Figure S28. Instrument set-up and custom-made coin cell for in-operando PXRD measurements.

(A) In-operando battery holder for coin cell and the Bragg-Brentano configuration of in-operando PXRD measurements. **(B)** Schematic illustration of the custom-made coin cell for in-operando PXRD measurements.

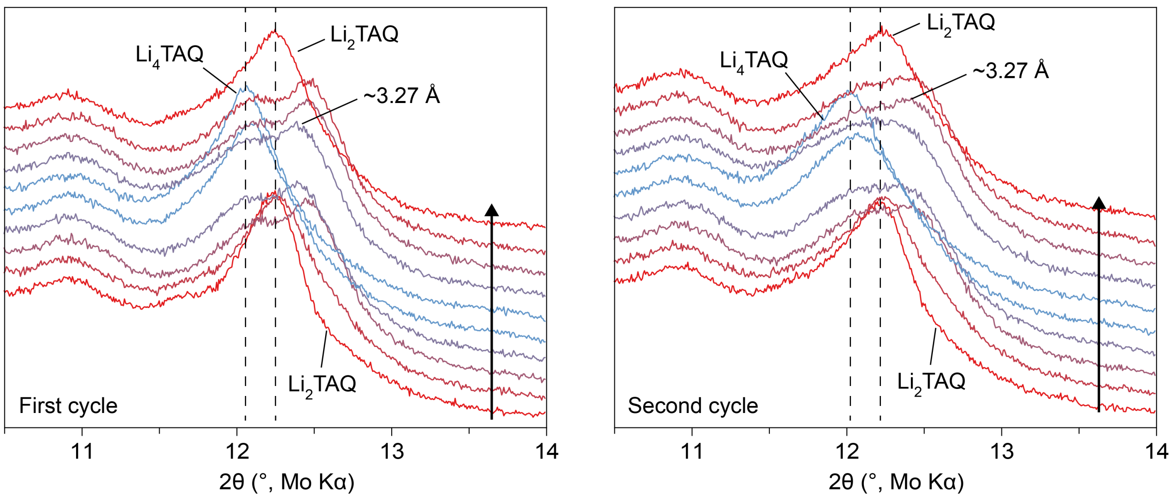

Figure S29.

In-operando PXRD patterns of the TAQ cell between Li_2TAQ and Li_4TAQ, exhibiting pronounced fluctuations of interlayer distance and complicated PXRD characteristics.

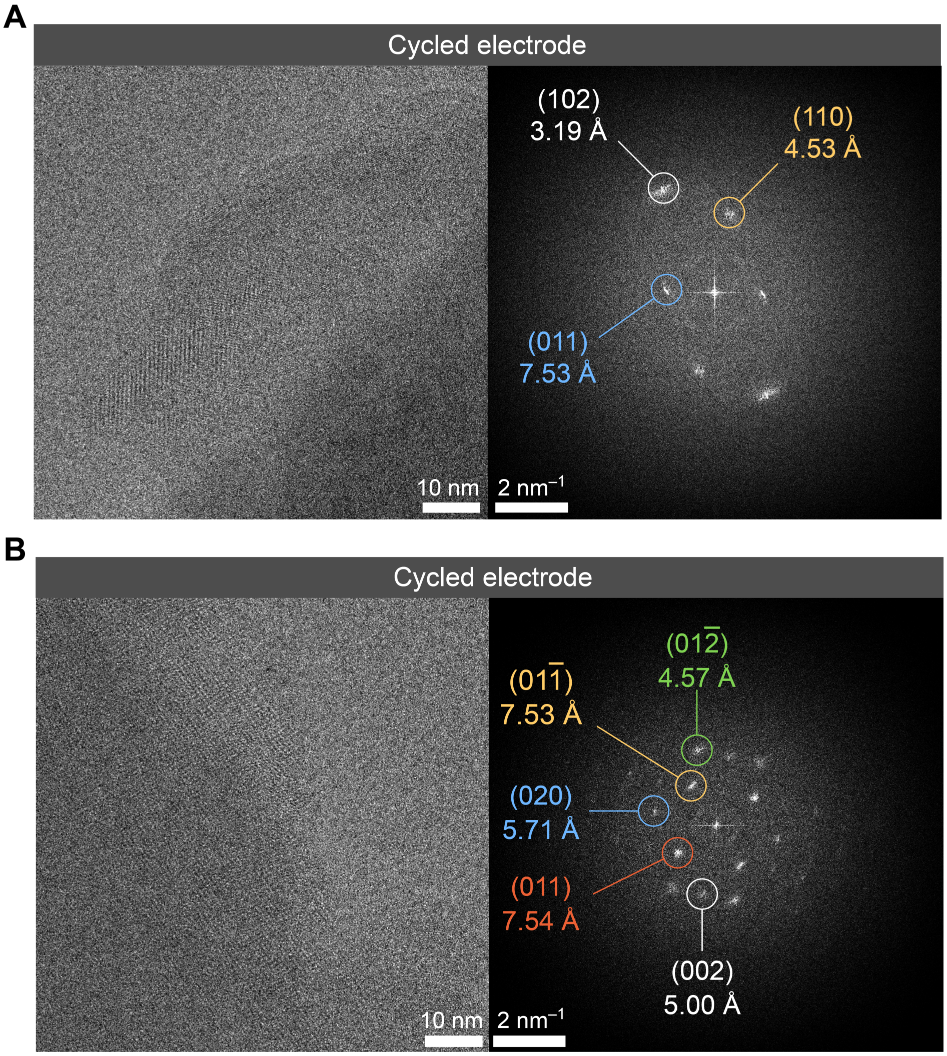

Figure S30.
Ex-situ Cryo-EM images and the corresponding FFT of cycled TAQ with indexed diffraction spots and corresponding *d* spacings. See Supplementary text for detailed analysis.

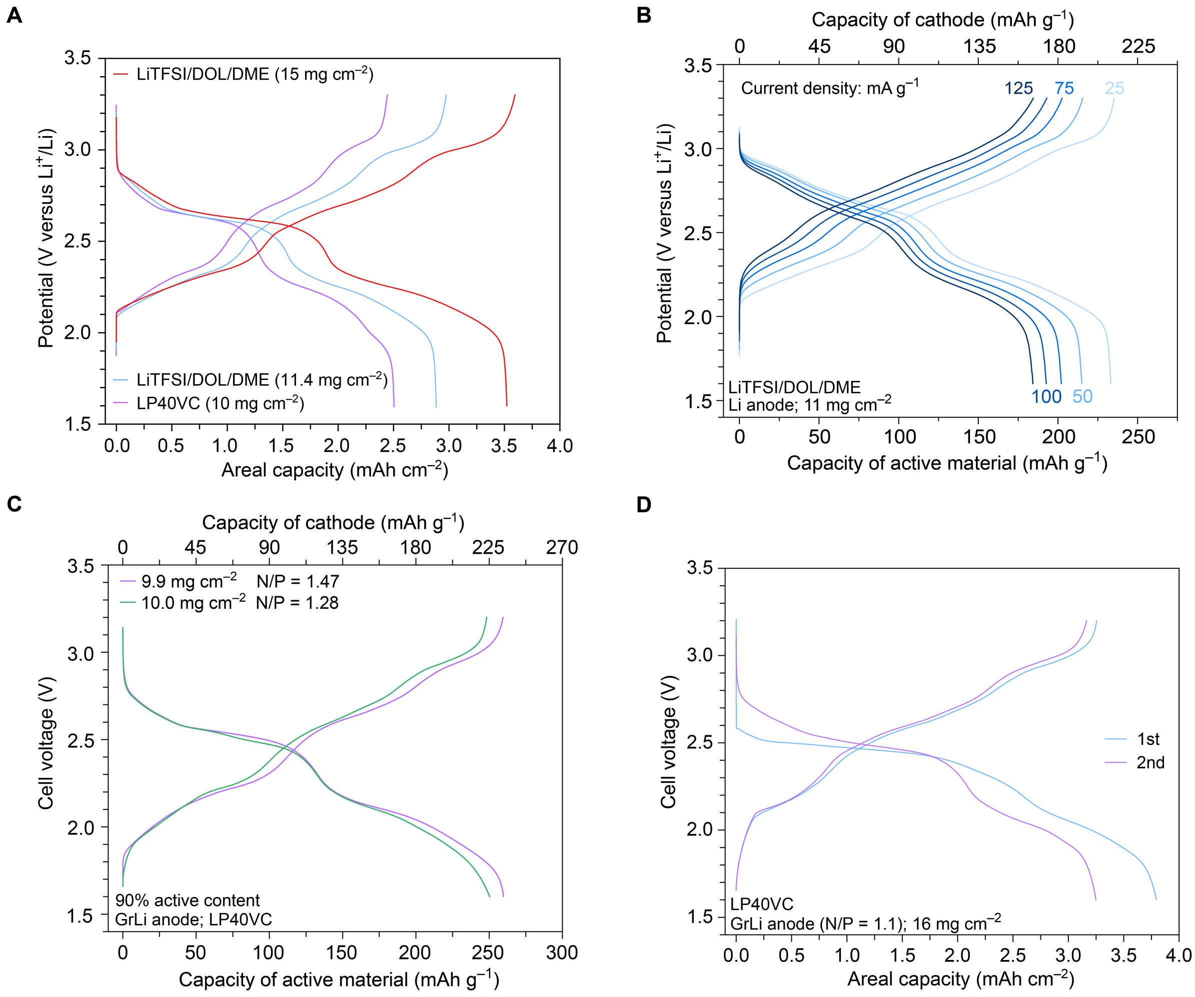

Figure S31. Battery performance of half and full cells based on TAQ/CMC/SBR composite electrodes with 90% active content.

(A) GCD voltage profiles of TAQ/CMC/SBR||Li cells with high active-material mass loadings (> 10 mg cm^{-2}) in LP40VC and LiTFSI/DOL/DME electrolytes. Areal capacities up to 3.52 mAh cm^{-2} are obtained. **(B)** Voltage profiles of a TAQ/CMC/SBR||Li cell with increasing current densities. **(C, D)** Voltage profiles of TAQ/CMC/SBR||GrLi full cells in LP40VC, exhibiting a reversible areal capacity of 3.25 mAh cm^{-2}.

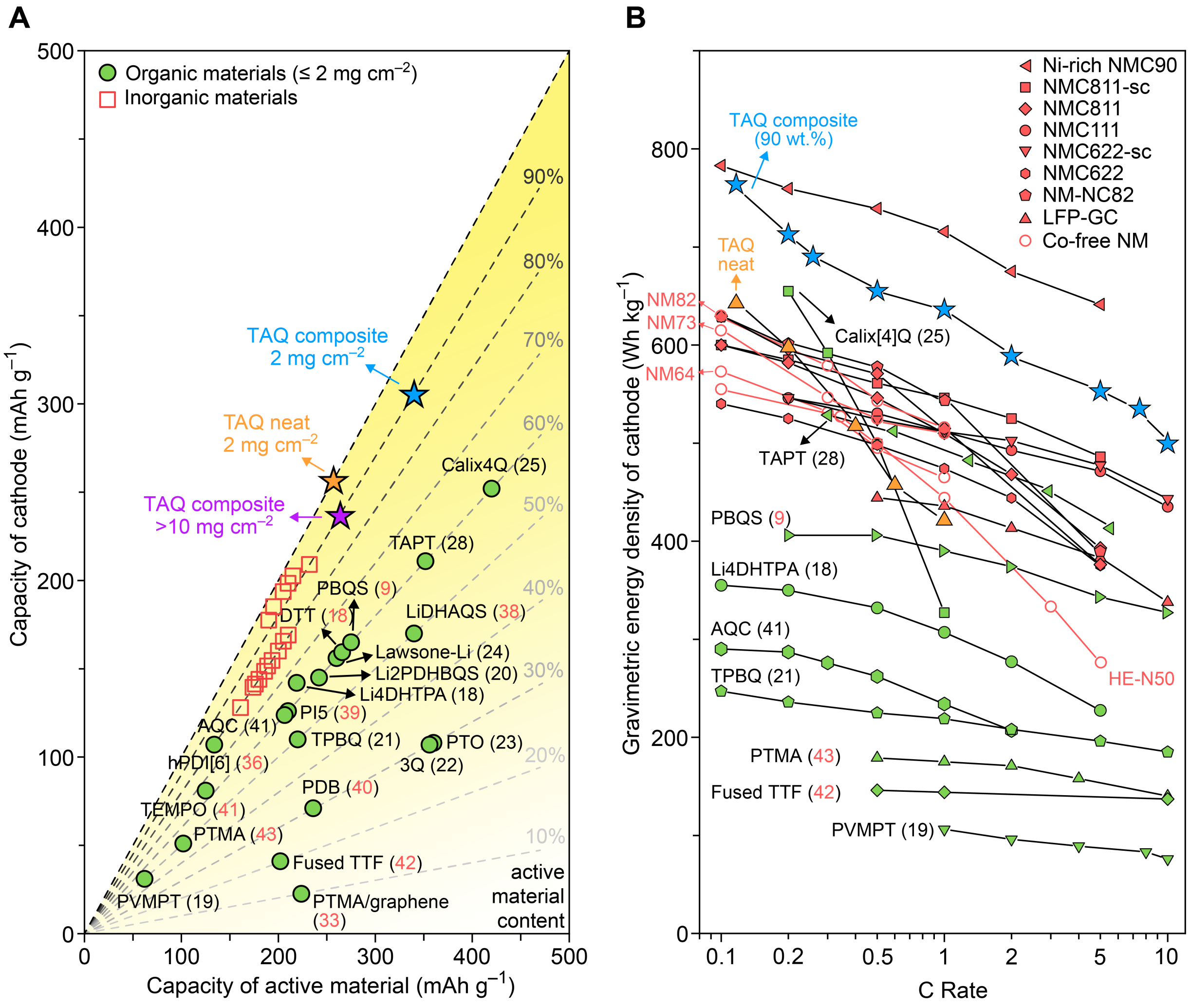

Figure S32.

Benchmarking TAQ performance against state-of-the-art cathodes. Reference numbers are presented in parentheses. The reference numbers in red indicate these references are cited in the Supporting Information.

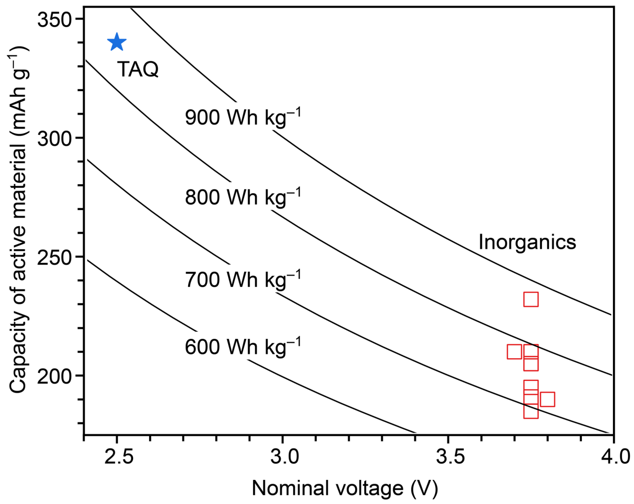

Figure S33.

Comparison of the nominal voltage, gravimetric specific capacity, and energy density of TAQ and inorganic cathode materials cited in Table S6.

Table S5.
Electrochemical performance of TAQ versus reported state-of-the-art organic cathode materials.

Active Material	Electrode composition (active material: conductive carbon: binder)	Highest specific capacity of active material (mAh/g)	Highest specific capacity of the electrode (mAh/g)	Average discharge voltage (V vs. Li^+/Li)	Electrode based energy density (Wh/kg)	References (Italic numbers indicate references in SI)
**Used in Figure 5C, S34:**						
LiDHAQS	50:40:10	330	**165**	2.5	412 at 0.1C	*38*
Lawsone-Li	60:30:10	275	**165**	2.25	371 at 0.2C	24
Li_2PDHBQS	60:30:10	247	**148**	2.0	296 at 0.2C	20
PTO	30:60:10	360	**108**	2.74	295 at 0.1C	23
PI5	60:30:10	210	**126**	2.4	303 at 1C	*39*
PDB	30:60:10	236	**71**	2.5	177 at 0.1C	*40*
3Q	30:60:10	356	**108**	1.7	182 at 1C	22
TEMPO	65:30:5	125	**81**	3.4	275 at 0.5C	*41*
**Used in Figure 5C, 5D, S34:**						
Li_4DHTPA	65:30:5	219	**142**	2.5	355 at 0.1C<br>345 at 0.2C<br>327 at 0.5C<br>315 at 1C<br>227 at 5C	18
Calix[4]quinone	60:30:10	420	**252**	2.6	655 at 0.2C<br>592 at 0.3 C<br>499 at 0.5 C<br>327 at 1C	25
TPBQ	50:40:10	220	**110**	2.25	247 at 0.1C<br>225 at 0.5 C<br>219 at 1 C<br>208 at 2 C<br>196 at 5C	21
AQC	60:30:10	210	**126**	2.3	290 at 0.1C<br>287 at 0.2 C<br>262 at 0.5C<br>234 at 1C	41

PBQS	60:30:10	260	**156**	2.6	406 at 0.1C<br>405 at 0.2 C<br>390 at 1 C<br>374 at 2 C<br>343 at 5C	*9*
Pentakis-fused tetrathiafulvalene	20:70:10	202	**41**	3.6	146 at 0.5C<br>144 at 1 C<br>137 at 10C	*42*
PVMPT	50:40:10	62	**31**	3.45	106 at 1C<br>96 at 2C<br>89 at 4C<br>83 at 8C<br>76 at 10C	19
PTMA	50:40:10	110	**55**	3.5	192 at 0.1C<br>178 at 0.5 C<br>175 at 1C<br>171 at 2 C<br>158 at 5C	*43*
TAPT	60:30:10	353	**212**	2.5	531 at 0.3C<br>520 at 0.6C<br>484 at 1.5C<br>456 at 3C<br>432 at 5.6C	28
**TAQ**	**90:5:5**	**340**	**306 (0.125C)**<br>**223 (10C)**	**2.5**	**765 at 0.125C**<br>**714 at 0.2C**<br>**691 at 0.25C**<br>**656 at 0.5C**<br>**637 at 1C**<br>**590 at 2C**<br>**570 at 5C**<br>**540 at 7.5C**<br>**506 at 10C**	**This work**

Table S6.

Electrochemical performance of TAQ versus reported state-of-the-art inorganic cathode materials.

Active Material	Electrode composition (active material: carbon: Binder)	Highest specific capacity of active material (mAh/g)	Highest specific capacity of the electrode (mAh/g)	Average discharge voltage (V vs. Li^+/Li)	Electrode based energy density (Wh/kg)	References (main text)
**Used in Figure 5C, 5D, S34:**						
NMC622-SC	80:10:10	180	**144**	3.75	540 at 0.1C<br>525 at 0.2C<br>498 at 0.5C<br>474 at 1C<br>375 at 5C	47
NMC622	80:10:10	180	**144**	3.75	540 at 0.1C<br>393 at 5C	47
NMC811-SC	80:10:10	200	**160**	3.75	600 at 0.1C<br>585 at 0.2C<br>561 at 0.5C<br>546 at 1C<br>486 at 5C	47
NMC811	80:10:10	200	**160**	3.75	600 at 0.1C<br>582 at 0.2C<br>546 at 0.5C<br>510 at 1C<br>393 at 5C	47
NMC811	80:10:10	210	**168**	3.75	630 at 0.1C<br>600 at 0.2C<br>570 at 0.5C<br>514 at 1C<br>375 at 5C	51
NM-NC82 [1]	80:10:10	210	**168**	3.75	630 at 0.1C<br>609 at 0.2C<br>585 at 0.5C<br>544 at 1C<br>390 at 5C	51
H-NMC111	80:10:10	175	**140**	3.9	546 at 0.1C<br>530 at 0.5C<br>511 at 1C<br>492 at 2C<br>471 at 5C	50
LFP-GC	80:10:10	161	**129**	3.45	555 at 0.5C<br>545 at 1C<br>516 at 2C<br>479 at 5C	48

					422 at 10 C	
Ni-Rich<br>NMC90	90:5.5:4.5	232	**209**	3.75	784 at 0.1C<br>760 at 0.2C<br>739 at 0.5C<br>716 at 1C<br>675 at 2C<br>641 at 5 C	49
NMC532	80:10:10	189	**151.2**	3.75	567 at 0.1C<br>538.7 at<br>0.33C<br>510.3 at 0.5C<br>442.3 at 1C<br>328.9 at 3C<br>198.4 at 5C	52
HE-N50 [1]	80:10:10	185	**148**	3.75	555 at 0.1C<br>527.2 at<br>0.33C<br>499.5 at 0.5C<br>444 at 1C<br>333 at 3C<br>276 at 5C	52
NM64	80:10:10	191	**152.8**	3.75	573 at 0.1C<br>531 at 0.3C<br>495 at 0.5C<br>465 at 1C	53
NM73	80:10:10	205	**164**	3.75	615 at 0.1C<br>546 at 0.3C<br>525 at 0.5C<br>510 at 1C	53
NM82	80:10:10	210	**168**	3.75	630 at 0.1C<br>579 at 0.3C<br>543 at 0.5C<br>516 at 1C	53
NCM811 [3]	94:3:3	215	**202**	3.7	748 at 0.2C	54
NCM721 [3]	94:3:3	210	**197**	3.7	730 at 0.2C	54
NCM622 [3]	94:3:3	205	**193**	3.75	723 at 0.2C	54
NCM523 [3]	93:4:3	195	**181**	3.8	689 at 0.2C	54
NCM111 [3]	93:4:3	190	**177**	3.8	671 at 0.2C	54
**TAQ**	**90:5:5**	**340**	**306**	**2.5**	**765 at 0.125C**<br>**714 at 0.2C**<br>**691 at 0.25C**<br>**656 at 0.5C**<br>**637 at 1C**<br>**590 at 2C**<br>**570 at 5C**<br>**540 at 7.5C**	**This work**

					**506 at 10C**	
**TAQ**	**100:0:0**	**258**	**258**	**2.5**	**645 at 0.125C**<br>**600 at 0.2C**<br>**460 at 0.625C**<br>**418 at 1C**	**This work**

[1] $0.5(LiNi_{0.8}Mn_{0.2}O_2)_{bulk}0.5(LiNi_{0.8}Co_{0.2}O_2)_{surface}$ [2] $LiNi_{0.5}Mn_{0.43}Ti_{0.02}Mg_{0.02}Nb_{0.01}Mo_{0.02}O_2$. [3] No rate data available, only used for Figure 5C.

Table S7. Comparison of essential energy storage parameters between commercial inorganic cathode materials and TAQ. High specific capacity values of TAQ coupled with its modest nominal voltage lead to higher gravimetric energy density than both NMC811 and $LiFePO_4$. Moreover, despite a relatively low material density, TAQ can offer higher volumetric energy density than $LiFePO_4$. *Calendaring TAQ electrodes is expected to increase the electrode density by 1.5-fold. Typical increases in density before and after calendaring are in the range of 1.3 – 1.6-fold for various published cathode and anode materials.[44–46]

Material	Specific capacity of active material (mAh/g)	Nominal voltage (V vs. Li^+/Li)	Energy density (Wh/kg)	Coated electrode density (g/cm^3)	Calendared electrode density (g/cm^3)	Volumetric Energy density (Wh/l)
**TAQ**	**340**	**2.50**	**850**	**1.1**	**1.7***	**935 – 1445**
NMC811	220	3.75	825	2.6	3.3	2125 – 2722
$LiFePO_4$	160	3.40	550	1.6	2.4	880 – 1320

REFERENCES

1. T. Chen, J.-H. Dou, L. Yang, C. Sun, J. J. Oppenheim, J. Li, M. Dincă, Dimensionality modulates electrical conductivity in compositionally constant one-, two-, and three-dimensional frameworks. *J. Am. Chem. Soc.* **144**, 5583–5593 (2022).

2. L. Sun, S. S. Park, D. Sheberla, M. Dincă, Measuring and reporting electrical conductivity in metal-organic frameworks: Cd_2(TTFTB) as a case study. *J. Am. Chem. Soc.* **138**, 14772–14782 (2016).

3. T. Chen, J.-H. Dou, L. Yang, C. Sun, N. J. Libretto, G. Skorupskii, J. T. Miller, M. Dincă, Continuous electrical conductivity variation in M_3(hexaiminotriphenylene)$_2$ (M = Co, Ni, Cu) MOF alloys. *J. Am. Chem. Soc.***142**, 12367–12373 (2020).

4. S. Stoll, A. Schweiger, EasySpin, a comprehensive software package for spectral simulation and analysis in EPR. *J. Magn. Reson.* **178**, 42–55 (2006).

5. F. J. Dyson, Electron spin resonance absorption in metals. II. Theory of electron diffusion and the skin effect. *Phys. Rev.* **98**, 349–359 (1955).

6. Y. Yoon, B. Yan, Y. Surendranath, Suppressing ion transfer enables versatile measurements of electrochemical surface area for intrinsic activity comparisons. *J. Am. Chem. Soc.***140**, 2397–2400 (2018).

7. W. Weppner, R. A. Huggins, *J. Electrochem. Soc.* **124**, 1569–1578 (1977).

8. H. Tanaka, M. Hirate, S. Watanabe, S.-I. Kuroda, Microscopic signature of metallic state in semicrystalline conjugated polymers doped with fluoroalkylsilane molecules. *Adv. Mater.* **26**, 2376–2383 (2014).

9. Z. Song, Y. Qian, T. Zhang, M. Otani, H. Zhou, Poly(benzoquinonyl sulfide) as a high-energy organic cathode for rechargeable Li and Na batteries. *Adv. Sci.* **2**, 1500124 (2015).

10. Z. Song, H. Zhan, Y. Zhou, Anthraquinone based polymer as high performance cathode material for rechargeable lithium batteries. *Chem. Commun.* 448–450 (2009).

11. Y. Hanyu, Y. Ganbe, I. Honma, Application of quinonic cathode compounds for quasi-solid lithium batteries. *J. Power Sources* **221**, 186–190 (2013).

12. M. Yao, H. Senoh, S.-I. Yamazaki, Z. Siroma, T. Sakai, K. Yasuda, High-capacity organic positive-electrode material based on a benzoquinone derivative for use in rechargeable lithium batteries. *J. Power Sources* **195**, 8336–8340 (2010).

13. H. Kim, J. E. Kwon, B. Lee, J. Hong, M. Lee, S. Y. Park, K. Kang, High energy organic cathode for sodium rechargeable batteries. *Chem. Mater.* **27**, 7258–7264 (2015).

14. H. Kim, D.-H. Seo, G. Yoon, W. A. Goddard III, Y. S. Lee, W.-S. Yoon, K. Kang, The reaction mechanism and capacity degradation model in lithium insertion organic cathodes, $Li_2C_6O_6$, using combined experimental and first principle studies. *J. Phys. Chem. Lett.* 5, 3086–3092 (2014).

15. H. Chen, M. Armand, G. Demailly, F. Dolhem, P. Poizot, J. M. Tarascon, From biomass to a renewable $Li_xC_6O_6$ organic electrode for sustainable Li-ion batteries. *ChemSusChem* **1**, 348–355 (2008).

16. M. Lee, J. Hong, J. Lopez, Y. Sun, D. Feng, K. Lim, W. C. Chueh, M. F. Toney, Y. Cui, Z. Bao. High-performance sodium–organic battery by realizing four-sodium storage in disodium rhodizonate. *Nat. Energy* **2**, 861–868 (2017).

17. M. Yao, H. Senoh, T. Sakai, T. Kiyobayashi, 5,7,12,14-Pentacenetetrone as a high-capacity organic positive-electrode material for use in rechargeable lithium batteries. *Int. J. Electrochem. Sci.* **6**, 2905–2911 (2011).

18. T. Ma, Q. Zhao, J. Wang, Z. Pan, J. Chen, A sulfur heterocyclic quinone cathode and a multifunctional binder for a high-performance rechargeable lithium-ion battery. *Angew. Chem. Int. Ed.* **55**, 6428–6432 (2016).

19. X. Y. Han, C. X. Chang, L. J. Yuan, T. L. Sun, J. Sun, Aromatic carbonyl derivative polymers as high-performance Li-ion storage materials. *Adv. Mater.* **19**, 1616–1621 (2007).

20. L. Tao, J. Zhao, J. Chen, C. Ou, W. Lv, S. Zhong, 1,4,5,8-Naphthalenetetracarboxylic dianhydride grafted phthalocyanine macromolecules as an anode material for lithium ion batteries. *Nanoscale Adv.* **3**, 3199–3215 (2021).

21. W. Luo, M. Allen, V. Raju, X. Ji, An organic pigment as a high-performance cathode for sodium-ion batteries. *Adv. Energy Mater.* **4**, 1400554 (2014).

22. G. S. Vadehra, R. P. Maloney, M. A. Garcia-Garibay, B. Dunn, Naphthalene diimide based materials with adjustable redox potentials: Evaluation for organic lithium-ion batteries. *Chem. Mater.* **26**, 7151–7157 (2014).

23. W. Deng, Y. Shen, J. Qian, Y. Cao, H. Yang, A perylene diimide crystal with high capacity and stable cyclability for Na-ion batteries. *ACS Appl. Mater. Interfaces* **7**, 21095–21099 (2015).

24. Y. Hanyu, I. Honma, Rechargeable quasi-solid state lithium battery with organic crystalline cathode. *Sci. Rep.* **2**, 453 (2012).

25. Y. Lu, X. Hou, L. Miao, L. Li, R. Shi, L. Liu, J. Chen, Cyclohexanehexone with ultrahigh capacity as cathode materials for lithium-ion batteries. *Angew. Chem. Int. Ed.* 58, 7020–7024 (2019).

26. Y. Liang, P. Zhang, S. Yang, Z. Tao, J. Chen, Fused heteroaromatic organic compounds for high-power electrodes of rechargeable lithium batteries. *Adv. Energy Mater.* **3**, 600–605 (2013).

27. J. Hong, M. Lee, B. Lee, D. H. Seo, C. B. Park, K. Kang, Biologically inspired pteridine redox centres for rechargeable batteries. *Nat. Commun.* **5**, 5335 (2014).

28. J. Wang, A. E. Lakraychi, X. Liu, L. Sieuw, C. Morari, P. Poizot, A. Vlad, Conjugated sulfonamides as a class of organic lithium-ion positive electrodes. *Nat. Mater.* **20**, 665–673 (2021).

29. J. Li, H. Zhan, Y. Zhou, Synthesis and electrochemical properties of polypyrrole-coated poly (2,5-dimercapto-1,3,4-thiadiazole). *Electrochem. Commun.* **5**, 555–560 (2003).

30. K. Nakahara, S. Iwasa, M. Satoh, Y. Morioka, J. Iriyama, M. Suguro, E. Hasegawa, Rechargeable batteries with organic radical cathodes. *Chem. Phys. Lett.* **359**, 351–354 (2002).

31. K. Nakahara, J. Iriyama, S. Iwasa, M. Suguro, M. Satoh, E. J. Cairns, Al-laminated film packaged organic radical battery for high-power applications. *J. Power Sources* **163**, 1110–1113 (2007).

32. Y. Zhang, A. Park, A. Cintora, S. R. McMillan, N. J. Harmon, A. Moehle, M. E. Flatté, G. D. Fuchs, C. K. Ober, Impact of the synthesis method on the solid-state charge transport of radical polymers. *J. Mater. Chem. C* **6**, 111–118 (2018).

33. W. Guo, Y. X. Yin, S. Xin, Y. G. Guo, L. J. Wan, Superior radical polymer cathode material with a two-electron process redox reaction promoted by graphene. *Energy Environ. Sci.* **5**, 5221–5225 (2012).

34. Y. Liang, Z. Chen, Y. Jing, Y. Rong, A. Facchetti, Y. Yao, Heavily n-dopable π-conjugated redox polymers with ultrafast energy storage capability. *J. Am. Chem. Soc.* **137**, 4956–4959 (2015).

35. J. Kim, H. S. Park, T. H. Kim, S. Y. Kim, H. K. Song, An inter-tangled network of redox-active and conducting polymers as a cathode for ultrafast rechargeable batteries. *Phys. Chem. Chem. Phys.* **16**, 5295–5300 (2014).

36. Z. Jin, Q. Cheng, S. T. Bao, R. Zhang, A. M. Evans, F. Ng, Y. Xu, M. L. Steigerwald, A. E. McDermott, Y. Yang, C. Nuckolls, Iterative synthesis of contorted macromolecular ladders for fast-charging and long-life lithium batteries. *J. Am. Chem. Soc.* **144**, 13973–13980 (2022).

37. T. Le Gall, K. H. Reiman, M. C. Grossel, J. R. Owen, Poly (2,5-dihydroxy-1,4-benzoquinone-3,6-methylene): a new organic polymer as positive electrode material for rechargeable lithium batteries. *J. Power Sources* **119**, 316–320 (2003).

38. A. Petronico, K. L. Bassett, B. G. Nicolau, A. A. Gewirth, R. G. Nuzzo, Toward a four-electron redox quinone polymer for high capacity lithium ion storage. *Adv. Energy Mater.* **8**, 1700960 (2018).

39. J. Qin, Q. Lan, N. Liu, F. Men, X. Wang, Z. Song, H. Zhan, A metal-free battery with pure ionic liquid electrolyte. *iScience* **15**, 16–27 (2019).

40. J. Xie, Z. Wang, Z. J. Xu, Q. Zhang, Toward a high-performance all-plastic full battery with a single organic polymer as both cathode and anode. *Adv. Energy Mater.* **8**, 1703509 (2018).

41. H. W. Kim, H. J. Kim, H. Byeon, J. Kim, J. W. Yang, Y. Kim, J. K. Kim, Binder-free organic cathode based on nitroxide radical polymer-functionalized carbon nanotubes and gel polymer electrolyte for high-performance sodium organic polymer batteries. *J. Mater. Chem. A* **8**, 17980–17986 (2020).

42. M. Kato, K.-I. Senoo, M. Yao, Y. Misaki, A pentakis-fused tetrathiafulvalene system extended by cyclohexene-1,4-diylidenes: a new positive electrode material for rechargeable batteries utilizing ten electron redox. *J. Mater. Chem. A* **2**, 6747–6754 (2014).

43. K. Nakahara, J. Iriyama, S. Iwasa, M. Suguro, M. Satoh, E. J. Cairns, Cell properties for modified PTMA cathodes of organic radical batteries. *J. Power Sources* **165**, 398–402 (2007).

44. W. Li, E. M. Erickson, A. Manthiram, High-nickel layered oxide cathodes for lithium-based automotive batteries. *Nat. Energy* **5**, 26–34 (2020).

45. X. Ren, Z. Li, Y. Zheng, W. Tian, K. Zhang, J. Cao, S. Tian, J. Guo, L. Wen, G. Liang. High Volumetric Energy Density of $LiFePO_4$ Battery Based on Ultrasonic Vibration Combined with Thermal Drying Process. *J. Electrochem. Soc.* **167**,130523 (2020).

46. M. Abdollahifar, H. Cavers, S. Scheffler, A. Diener, M. Lippke, A. Kwade, A. Insights into Influencing Electrode Calendering on the Battery Performance. *Adv. Energy Mater.* **13**, 2300973 (2023).
